# Circulating serum metabolites as predictors of dementia: a machine learning approach in a 21-year follow-up of the Whitehall II cohort study

**DOI:** 10.1186/s12916-022-02519-6

**Published:** 2022-09-27

**Authors:** Marcos D. Machado-Fragua, Benjamin Landré, Mathilde Chen, Aurore Fayosse, Aline Dugravot, Mika Kivimaki, Séverine Sabia, Archana Singh-Manoux

**Affiliations:** 1 Université de Paris, Inserm U1153, Epidemiology of Ageing and Neurodegenerative Diseases, 10 Avenue de Verdun, 75010 Paris, France; 2 Department of Epidemiology and Public Health, University College London, London, UK

**Keywords:** Biomarkers, Metabolites, Dementia, Risk score, Predictive accuracy, C-statistic, Longitudinal study

## Abstract

**Background:**

Age is the strongest risk factor for dementia and there is considerable interest in identifying scalable, blood-based biomarkers in predicting dementia. We examined the role of midlife serum metabolites using a machine learning approach and determined whether the selected metabolites improved prediction accuracy beyond the effect of age.

**Methods:**

Five thousand three hundred seventy-four participants from the Whitehall II study, mean age 55.8 (standard deviation (SD) 6.0) years in 1997–1999 when 233 metabolites were quantified using nuclear magnetic resonance metabolomics. Participants were followed for a median 21.0 (IQR 20.4, 21.7) years for clinically-diagnosed dementia (*N*=329). Elastic net penalized Cox regression with 100 repetitions of nested cross-validation was used to select models that improved prediction accuracy for incident dementia compared to an age-only model. Risk scores reflecting the frequency with which predictors appeared in the selected models were constructed, and their predictive accuracy was examined using Royston’s *R*^2^, Akaike’s information criterion, sensitivity, specificity, C-statistic and calibration.

**Results:**

Sixteen of the 100 models had a better c-statistic compared to an age-only model and 15 metabolites were selected at least once in all 16 models with glucose present in all models. Five risk scores, reflecting the frequency of selection of metabolites, and a 1-SD increment in all five risk scores was associated with higher dementia risk (HR between 3.13 and 3.26). Three of these, constituted of 4, 5 and 15 metabolites, had better prediction accuracy (c-statistic from 0.788 to 0.796) compared to an age-only model (c-statistic 0.780), all *p*<0.05.

**Conclusions:**

Although there was robust evidence for the role of glucose in dementia, metabolites measured in midlife made only a modest contribution to dementia prediction once age was taken into account.

**Supplementary Information:**

The online version contains supplementary material available at 10.1186/s12916-022-02519-6.

## Background

Dementia is a complex disease and is the seventh leading cause of death worldwide [1]. Although the causes of dementia remain elusive, previous research suggests alterations in several pathways, suggesting that it is a multi-systemic disease [2–4]. Pathophysiological changes underlying dementia unfold over a long period, perhaps as long as 15 to 20 years [5]. Along with failure of therapeutic trials in this domain, the long preclinical phase of dementia has increased interest in prevention. It is within this framework that there is emerging research on risk factors and biomarkers measured in mid-life, before the onset of pathophysiological processes underlying dementia.

Cerebrospinal fluid (CSF) and imaging biomarkers are widely used in the diagnosis of Alzheimer’s disease, a major subtype of dementia, and there is increasing interest in blood-based diagnostic biomarkers as they are less invasive, and can readily be used in healthcare and research settings [6]. Whether biomarkers can be used for identifying prevention targets remains unclear. Metabolites are small molecules present in cells, tissues and biofluids, including blood. They reflect physiological and pathological processes and gene-environment interactions involving multiple body systems [7], making them potential biomarkers. However, much of the existing research typically assess metabolites late in life, not allowing the results to be meaningful for prevention [8, 9].

Studies that examined the associations between metabolite panels and the risk of dementia [10–12], have two important limitations. One, the identification of pertinent metabolites was based on correction for multiple testing. When the number of multiple comparisons is large this method leads to several false negatives, and only metabolites with a very large effect size are identified. Two, most studies included age in the predictive model but did not consider whether the predictive accuracy was primarily due to age [10–15], which is the strongest albeit non-modifiable risk factor for dementia [16]. Inclusion of age as a predictor along with putative biomarkers in the predictive model is not optimal as this approach cannot distinguish the part of the prediction due to age and that due to the biomarkers being considered in the analyses, and the results could be driven by age rather than the biomarkers [17]. Our strategy that consists of comparing the predictive accuracy of a model composed of age and putative biomarkers and one composed of age alone allows this limitation to be addressed.

Our aim was to identify metabolites associated with incident dementia independently of age over a 21-year follow-up, using machine-learning for survival analysis, namely elastic net penalized Cox regression. This method allows efficient selection of relevant predictors by simultaneously combining variable selection and shrinkage of coefficients; stability of the results was ensured using repeated resampling, and recalculation of effect estimates to select predictors with the most consistent association with the outcome [18, 19]. Explicit consideration of age in our algorithm to identify putative biomarkers was ensured by selecting sets of metabolites that improved predictive accuracy compared to an age-only model. This was achieved by constructing risk scores, constituted first using age alone and subsequently using age along with selected metabolites in order to test whether metabolites improved dementia prediction over and above the effect of age.

## Methods

### Study population

The Whitehall II study is an ongoing cohort study established in 1985–1988 among 10,308 persons (6895 men and 3413 women, aged 35–55 years) employed in London-based government departments [20]. Written informed consent from participants and research ethics approvals were renewed at each contact; the most recent approval was from the University College London Hospital Committee on the Ethics of Human Research, reference number 85/0938. Since baseline, follow-up clinical examinations have taken place approximately every 4 to 5 years (1991–1993, 1997–1999, 2002–2004, 2007–2009, 2012–2013, and 2015–2016). Data over the follow-up were also available using linkage to electronic health records of the UK National Health Service (NHS) for all but ten of the 10,308 participants recruited to the study. The NHS provides most of the health care in the country, and record linkage is undertaken using a unique NHS identifier held by all UK residents. Data from linked records were updated on an annual basis, until 31st of March 2019.

### Measures

#### Serum sample collection and metabolite panel (1997–1999)

Fasting serum was collected at each clinical examination in the study and stored at −80°C. For the present study, samples were taken from 1997 to 1999, and 233 metabolic biomarkers were analysed as part of the Consortium of Metabolomics Studies in 2014 using a high throughput Nuclear Magnetic Resonance (NMR) metabolomics platform, the Nightingale platform (Helsinki, Finland) [21]. All metabolites were measured in a single experimental set-up that allows simultaneous quantification of (a) total lipid concentrations of lipoprotein subclasses (very low-density lipoproteins (VLDL), intermediate-density lipoproteins (IDL), low-density lipoproteins (LDL), and high-density lipoproteins (HDL)); (b) lipoprotein ratios; (c) cholesterol related metabolites; (d) lipid-related metabolites; (e) fatty acids related metabolites; (f) apolipoproteins related metabolites; (g) glycolysis-related metabolites; (h) amino acids; (i) ketone bodies; (j) fluid balance metabolites; and (k) inflammation related metabolites. A full list of the 233 metabolites included in the study can be found in eTable 1. The platform processes data automatically and executes quality procedures reporting degradation and contamination issues. The metabolite value was set at 0 when its concentration was above the limit of detection but below the limit of quantification due to biological reasons or external compounds interfering with quantification. Every metabolite that has been reported in the results file has passed this strict quality control procedure; outlier values in metabolite concentrations (≥±9 SD) were excluded. Please note that incomplete data on metabolites may be due to metabolite concentrations under the limit of detection as well as non-participation in the clinical examination at the 1997–1999 wave.

#### Dementia

Ascertainment of dementia was undertaken using linkage to Hospital Episode Statistics (HES), the Mental Health Services Data Set (MHSDS), and the mortality register using ICD-10 codes F00-F03, F05.1, G30, and G31. HES contains clinical diagnoses from inpatient and outpatient clinical encounters in English acute generals hospitals and has sensitivity and specificity of 78.0% and 92.0%, respectively [22]. MHSDS contains dementia diagnoses from inpatient, outpatient and community mental health services, including memory clinics, and the British national mortality register collects information about cause-specific mortality. Record linkage was available until 31st of March 2019, and the date of dementia was set at the first record of dementia diagnosis in any of these three databases.

#### Sociodemographic variables

Sociodemographic variables included age, sex, ethnicity (white and non-white) and education, measured as the highest qualification on leaving full-time education and categorized as high (university or higher degree), intermediate (higher secondary school), or low (lower secondary school or less).

### Statistical analysis

Participants’ characteristics and metabolites concentrations in 1997–1999 were examined as a function of dementia status at the end of follow-up using *χ*^2^ test and Student’s *t*-test, as appropriate. All metabolite concentrations were first log-transformed to obtain approximately normal distribution and then standardized to *z*-scores (mean=0, standard deviation (SD)=1). Two types of analyses were undertaken, the first using Cox regression with Bonferroni correction for multiple testing and the second using a machine learning approach.

The association between 1 SD increment in each metabolite, analysed individually, and incident dementia was examined using Cox regression. The start of follow-up was the date of the 1997–1999 clinical examination, and participants were censored at date of dementia diagnosis, death, or 31st of March 2019, whichever came first. The analyses were adjusted for sociodemographic variables (age, sex, education and ethnicity); Bonferroni correction [23] implied use of a *p*-value of 0.0002 (0.05/233).

**Fig. 1:**
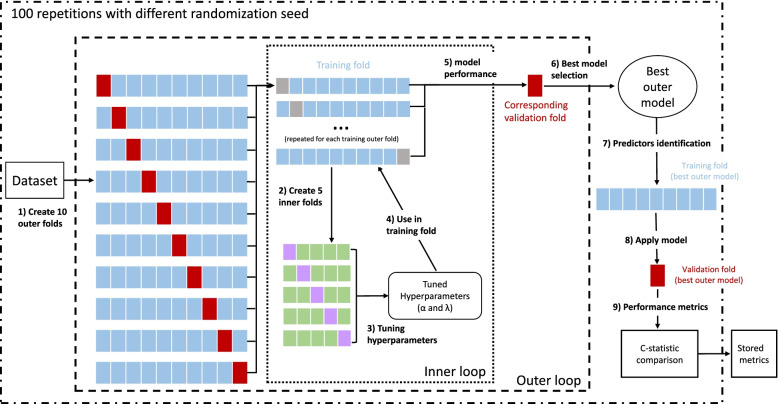
Scheme of the repeated nested cross-validation procedure. The following procedure was repeated 100 times to account for variation in results due to random partitioning of the cross-validation folds. The steps in the analyses are (1) Partition dataset into 10 outer folds with the same dementia rate in each fold. (2) Further partition each training outer fold (blue boxes) into 5 inner folds (same dementia rate) to build the inner loop. Grey boxes represent the validation folds (outer loop) which are not involved in the inner loop. 3) Use inner folds to tune the hyperparameters, select best combination of α and λ (model with the lowest partial likelihood deviance in the inner loop). (4) Apply selected hyperparameters to the corresponding training outer fold. (5) Evaluate model performance in the corresponding outer validation fold (red box). (6) Choose the best of 10 outer models (lowest partial likelihood deviance). (7) Identify predictors (variables with non-zero beta-coefficients) in the training fold of the best model in the outer fold. (8) Apply the best outer model hyperparameters to the corresponding validation outer fold. (9) Compare the c-statistic of the prediction model to the c-statistic of an age-specific model in the same validation outer fold

The second method was elastic net penalized Cox regression, a regularization technique that allows simultaneous selection of predictors and shrinkage of the effect size. The steps in the analyses are shown in Fig. 1. A total of 237 predictors (233 metabolites and 4 sociodemographic variables—age, sex, education, and ethnicity) were used and repeated nested cross-validation [18, 24, 25] was used to separate parameter tuning and model selection and to address the problem of overfitting. A 5-fold inner loop was used to identify the best-tuned hyperparameters (α and λ) and a 10-fold outer loop for identifying the best set of predictors (steps 1 and 2), using the lowest cross-validation error (partial likelihood deviance) to define both optimal selections. Folds were stratified so that dementia rates were similar in each fold, and age was forced in all models. The hyperparameters α and λ were used to choose the number of predictors and for the optimal shrinkage of the beta-coefficients of the predictors, respectively. The inner loop was used to select the best α and λ and was performed on the training folds of the outer loop (step 3). Then the tuned hyperparameters from the previous step were used in the training data (outer loop; step 4) and the model performance was evaluated in the corresponding validation fold (step 5), selecting the best-performed model of the outer loop (step 6). Subsequently, predictors with non-zero coefficients were identified (step 7), and its C-statistic was compared to that from an age-only model in the same validation outer-fold (steps 8 and 9). The entire procedure was repeated 100 times to obtain stable results. Then, only the models that improved the c-statistic compared to an age-only model for predicting dementia (*p*-value for difference in c-statistic <0.05) were retained; the metabolites identified in these models were organized in five non-mutually exclusive groups: metabolites present in 100%, ≥90%, ≥60%, ≥50%, or at least once in the selected models. Then these groups were used to construct five risk scores using sum of the weighted (by frequency of occurrence in selected models) coefficients from Cox regression. Age on its own was also considered a risk score. The construction of risk scores with the metabolites allows the combination of several predictors into a single predictor so that when comparisons are made, in our case with age, there is a single predictor in each case, irrespective of the number of metabolites in the risk score.

All six risk scores were standardized to z-scores (mean=0, SD=1) and associations between 1 SD increment in risk scores and incident dementia were examined using Cox regression. The predictive accuracy of the risk scores was assessed using (a) Royston’s modified *R*^2^ to measure overall performance of the prediction model with confidence intervals calculated using 2000 bootstrap replications, with higher values indicating greater explained variance [26]; (b) Akaike information criterion (AIC), a measure of the relative goodness of fit of a statistical model; lower values indicate better model fit and a difference of 10 or more considered meaningful; (c) sensitivity and specificity for survival models as measures of classification accuracy using optimal threshold established by maximizing the Youden index [27]; and (d) Harrell’s C-statistic for survival models to measure discrimination, with the age-alone risk score as the reference [28]. In addition, the Greenwood-Nam-D’Agostino (GND) test was used to test calibration [29] to evaluate the agreement between observed and predicted risk, *p* < 0.05 indicating lack of fit, and calibration-in-the-large shown in plots of observed and predicted dementia rate per 1000 person/years in deciles of the risk scores (first and second decile were collapsed due to a small number of events). The C-statistic of these risk scores was formally compared using a nonparametric approach with the age-alone risk score as the reference [30].

We performed four sensitivity analyses. One, to examine the effect of excluding metabolite concentrations that were below the limit of quantification (value set a 0 for the Nightingale Health metabolomics platform) or outliers (≥±9 SD), Cox regression analyses with Bonferroni correction were repeated without these exclusions. Two, to examine the individual contribution of metabolites and rank them by their importance we examined change in the predictive accuracy of the score excluding one metabolite at a time from the risk score with the largest number of metabolites. Three, the role of the Apolipoprotein genotype was examined by adding ApoE e4 (yes/no) status to the risk scores in participants with data on this measure and predictive accuracy was examined as in the main analyses. Four, we compared the predictive accuracy of the best-performance risk score in our analyses with two sets of metabolites previously identified in conventional rather than a machine learning approach in meta-analyses that included the Whitehall II cohort study [11, 12].

Elastic net regression and GND test were performed using R software (version 4.1.0); all other analyses were undertaken using Stata (version 16). Two-sided *p*<0.05 was considered to be statistically significant.

## Results

Of the 10,308 participants at study inception in 1985–1988, 7870 (76.4%) participated in the 1997–1999 wave of data collection, the baseline of our analyses. Of these, we excluded 1333 (16.9%) participants who did not participate in the clinical examination at the 1997–1999 wave, 1093 (13,9%) participants with metabolite values under the limit of detection, and 70 (0.9%) participants with outlier values on metabolites, leading to analyses on 5374 (68.3%) participants (Additional file 1: Fig. S1). The mean (SD) age of participants at baseline was 55.8 (6.0) years, and 27.7% were women. Over a median follow-up 21.0 (IQR 20.4, 21.7) years, 329 (6.1%) participants were diagnosed with dementia and 953 (17.8%) died. Participants diagnosed with dementia were older, more likely to be women, and non-white and had lower education (Table 1). The mean (SD) of metabolite concentrations overall and as a function of dementia status at the end of follow-up are shown in Additional file 1: Table S1.

**Table 1:** Sample characteristics in 1997–1999 as a function of dementia status at the end of follow-up (31st March 2019)

	Dementia^*^		
	No	Yes	***p***-value^**†**^
N	5045 (93.9)	329 (6.1)	
Age at baseline, M (SD)	55.4 (5.9)	61.2 (4.7)	<0.001
Sex			
Men	3662 (72.6)	220 (66.9)	0.03
Women	1383 (27.4)	109 (33.1)	
Education			
Low	2198 (43.6)	181 (55.0)	
Medium	1335 (26.4)	66 (20.1)	<0.001
High	1512 (30.0)	82 (24.9)	
Ethnicity			
White	4656 (92.3)	288 (87.5)	0.002
Non-white	389 (7.7)	41 (12.5)	

Data are n (%), unless otherwise specified

^*^Data on dementia subtype was as follows: Alzheimer’s disease (*N*=137), vascular dementia (N=47), Parkinson’s dementia (*N*=17), mixed Alzheimer’s and vascular dementia (*N*=21), mixed vascular and Parkinson’s dementia (*N*=1), mixed Alzheimer’s and Parkinson’s dementia (*N*=5), other/missing subtype (*N*=101)

^†^*p*-value for difference in *χ*^2^ test (categorical data) or Student’s *t* test (continuous data)

### Associations between metabolites and dementia using Bonferroni correction

The hazard ratio (HR) and associated 95% confidence interval (CI) for 1-SD increment in metabolite concentrations and incident dementia, adjusted for sociodemographic variables are shown in Additional file 1: Table S2. At *p*<0.05, five metabolites were associated with risk of dementia (total cholesterol to total lipids ratio in chylomicrons and extremely large VLDL, HR (95% CI): 0.84 (0.73, 0.96); free cholesterol to total lipids ratio in chylomicrons and extremely large VLDL, HR (95% CI): 0.86 (0.74, 0.99); triglycerides to total lipids ratio in chylomicrons and extremely large VLDL, HR (95% CI): 1.17 (1.04, 1.33); phospholipids to total lipids ratio in medium HDL, HR (95% CI): 1.15 (1.03, 1.29); and glucose (mmol/l), HR (95% CI): 1.24 (1.13, 1.36)). However, glucose (*p*=0.00001) was the only metabolite associated with dementia using Bonferroni correction for multiple testing at *p*<0.0002. Further analyses excluding 229 (4.3%) participants with metabolite concentrations below the limit of quantification (Additional file 1: Table S3) and including 70 participants with outlier values (≥±9 SD; Additional file 1: Table S4) yielded results similar to those in the main analyses, glucose being the only metabolite associated with dementia after Bonferroni correction.

### Elastic net penalized Cox regression

Results of the 100 repetitions are shown in Additional file 1: Table S5; sixteen of these models had significantly better c-statistic than an age-only model, ranging from 0.703 to 0.779, Table 2. These models identified between 2 (repetition number 22) and 12 (repetition number 96) predictors. A total of 15 metabolites were identified at least once across the sixteen models; their frequency of selection is shown in Table 3. Glucose was the only metabolite identified in all 16 models. Besides age, which was forced in all models, no other sociodemographic variable (sex, ethnicity, or education) was selected by these models.

**Table 2:** Elastic net penalized Cox regression with repeated nested cross-validation: models (out of 100 repetitions) that improved prediction accuracy for incident dementia compared to an age-only model

Repetition number	α^*^	λ^*^	c-statistic of the best model^**†**^	c-statistic age-only model^**‡**^	***p***-value^**§**^	Number of predictors in the selected model^¶^
2	0.9	0.00617437	0.760	0.749	0.01	4
4	1	0.00617437	0.724	0.715	0.007	4
10	1	0.00677636	0.747	0.741	0.04	4
16	1	0.00512607	0.775	0.765	0.02	7
18	0.7	0.01299645	0.742	0.738	0.007	3
22	1	0.00816215	0.703	0.696	0.02	2
23	1	0.00425575	0.779	0.763	0.001	8
30	1	0.00617437	0.746	0.736	0.009	5
38	1	0.00617437	0.718	0.711	0.04	4
50	1	0.00562585	0.745	0.734	0.01	5
57	1	0.00467068	0.747	0.731	0.0006	9
67	0.5	0.00983134	0.755	0.743	0.004	6
74	0.8	0.00816215	0.735	0.726	0.02	3
91	1	0.00677636	0.724	0.714	0.008	3
94	0.7	0.00677636	0.762	0.751	0.03	9
96	0.5	0.00743705	0.735	0.722	0.04	12

^*^These are hyperparameters, allowing selection of the model with the lowest partial likelihood deviance in the inner loop; α ranges from 0 to 1 and when it is 0 all predictors are retained in the model, λ controls the coefficient shrinkage

^†^c-statistic, in the validation fold of the outer loop, of the best model (lowest partial likelihood deviance in the training folds of the outer loop)

^‡^c-statistic of the age-only model in the validation fold of the best outer loop model

^§^*p*-value for difference in C-statistic between the best model and the age-only model

^¶^Age was forced to be selected in all models.

**Table 3:** Frequency of metabolites identified by elastic net penalized Cox regression in the sixteen selected models

Name of metabolite	***N*** (%)
Glucose (mmol/l)	16/16 (100)
Phospholipids to total lipids ratio in medium HDL (%)	15/16 (93.8)
Creatinine (mmol/l)	10/16 (62.5)
Triglycerides to total lipids ratio in very large VLDL (%)	8/16 (50.0)
Phospholipids to total lipids ratio in medium VLDL (%)	5/16 (31.3)
Alanine (mmol/l)	5/16 (31.3)
β-hydroxybutyrate (mmol/l)	3/16 (18.8)
Free cholesterol to total lipids ratio in small HDL (%)	2/16 (12.5)
Citrate (mmol/l)	2/16 (12.5)
Free cholesterol to total lipids ratio in very large VLDL (%)	1/16 (6.3)
Free cholesterol to total lipids ratio in large HDL (%)	1/16 (6.3)
Triglycerides to total lipids ratio in medium HDL (%)	1/16 (6.3)
Phospholipids to total lipids ratio in small HDL (%)	1/16 (6.3)
Sphingomyelins (mmol/l)	1/16 (6.3)
Albumin (signal area)	1/16 (6.3)

*VLDL* very low-density lipoproteins, *HDL* high-density lipoprotein

The beta-coefficients associated with each predictor used in the calculation of risk scores are shown in Additional file 1: Table S6, organized as risk score 1 to 5 to reflect metabolites selected in 100%, ≥90%, ≥60%, ≥50%, or at least once in elastic net regression. Note that risk score 1, which included age and glucose (identified in 100% of the selected models) reflected the results obtained in the Cox regression with Bonferroni correction. The prediction statistics of the age-only model and the 5 risk scores are shown in Table 4. A 1-SD increment in all risk scores was associated with a higher risk of dementia (HR between 3.04 and 3.26). Three risk scores (3, 4, and 5) had a better c-statistic (*p*- <0.05) compared to the age-only model, with sensitivity from 72.4 to 77.0% and specificity from 69.1 to 72.7%. Risk score 5, which included age and 15 metabolites identified at least once in the elastic net models, had the highest HR (95% CI) 3.26 (2.87, 3.71), the best model fit (AIC 5147.7), the highest *R*^2^ at 0.582 (0.511, 0.649), and the highest c-statistic (0.796 (0.774, 0.819). Risk score 5 also had a better c-statistic when compared to all other risk scores (all *p*<0.05).

**Table 4:** Predictive performance of risk scores for incident dementia (*N*=5374)

Risk scores	HR (95% confidence interval)	***R***^**2**^ (95% confidence interval)	AIC	Δ AIC	Sensitivity %	Specificity %	c-statistic (95% confidence interval)	***p***-value^*^
Age-only model	3.04 (2.66, 3.47)	0.525 (0.450, 0.593)	5198.2	Ref.	75.5	70.3	0.780 (0.757, 0.802)	Ref.
Risk score 1^†^	3.13 (2.75, 3.57)	0.545 (0.468, 0.636)	5181.2	− 17.0	80.9	64.7	0.786 (0.763, 0.808)	0.05
Risk score 2^‡^	3.16 (2.77, 3.60)	0.551 (0.475, 0.620)	5176.1	− 22.1	81.5	64.2	0.787 (0.764, 0.809)	0.05
Risk score 3^§^	3.17 (2.78, 3.61)	0.557 (0.482, 0.630)	5170.8	− 27.4	77.0	69.1	0.788 (0.766, 0.811)	0.03
Risk score 4^¶^	3.19 (2.80, 3.63)	0.565 (0.492, 0.636)	5163.6	− 34.6	72.4	72.7	0.790 (0.767, 0.813)	0.02
Risk score 5^#^	3.26 (2.87, 3.71)	0.582 (0.511, 0.649)	5147.7	− 50.5	74.0	72.0	0.796 (0.774, 0.819)	<0.001

*R*^*2*^ Royston’s R^2^, *AIC* Akaike information criterion, *c-statistic* Harrell’s C-index, *VLDL* very low-density lipoproteins, *HDL* high-density lipoprotein

^*^p-value for difference in c-statistic using age-only model as reference

^†^Risk score 1 includes age and glucose

^‡^Risk score 2 includes age, glucose and phospholipids to total lipids ratio in medium HDL (%)

^§^Risk score 3 includes age, glucose, phospholipids to total lipids ratio in medium HDL (%) and creatinine (mmol/l)

^¶^Risk score 4 includes age, glucose, phospholipids to total lipids ratio in medium HDL (%), creatinine (mmol/l) and triglycerides to total lipids ratio in very large VLDL (%)

^#^Risk score 5 includes age, glucose, phospholipids to total lipids ratio in medium HDL (%), creatinine (mmol/l), triglycerides to total lipids ratio in very large VLDL (%), phospholipids to total lipids ratio in medium VLDL (%), alanine (mmol/l), 3-hydroxybutyrate (mmol/l), free cholesterol to total lipids ratio in small HDL (%), citrate (mmol/l), free cholesterol to total lipids ratio in very large VLDL (%), free cholesterol to total lipids ratio in large HDL (%), triglycerides to total lipids ratio in medium HDL (%), phospholipids to total lipids ratio in small HDL (%), sphingomyelins (mmol/l) and albumin (signal area)

Youden index cutoff points for the calculation of the sensitivity and specificity are as follows: 0.541 for the age-only model; 0.310 for risk score 1; 0.296 for risk score 2; 0.468 for risk score 3; 0.604 for risk score 4; and 0.564 for risk score 5

**Fig. 2:**
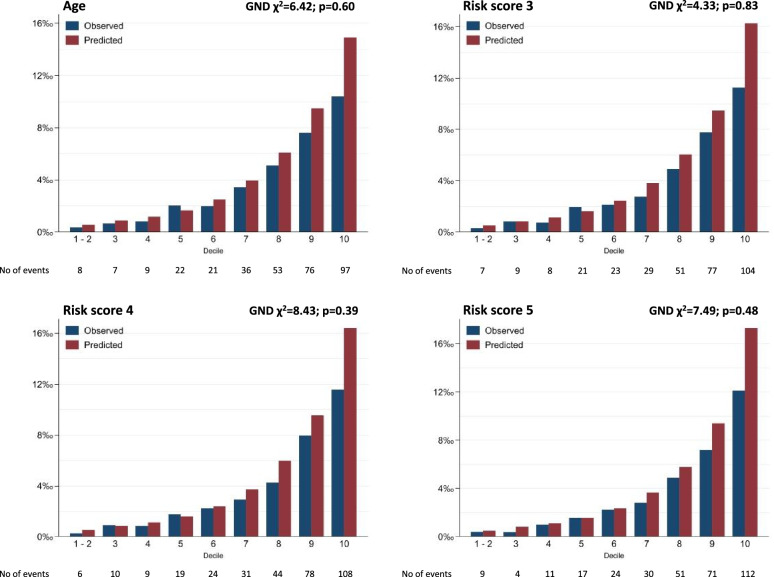
Observed and predicted rate of dementia per 1000 person-years (calibration-in-the-large) as a function of deciles of predictors (age, risk score 3, risk score 4 and risk score 5). VLDL, very low-density lipoproteins; HDL, high-density lipoprotein. The first and second decile were collapsed due to a small number of events in these deciles. Risk score 3 includes age, glucose, phospholipids to total lipids ratio in medium HDL (%) and creatinine (mmol/l). Risk score 4 includes age, glucose, phospholipids to total lipids ratio in medium HDL (%), creatinine (mmol/l), and triglycerides to total lipids ratio in very large VLDL (%). Risk score 5 includes age, glucose, phospholipids to total lipids ratio in medium HDL (%), creatinine (mmol/l), triglycerides to total lipids ratio in very large VLDL (%), phospholipids to total lipids ratio in medium VLDL (%), alanine (mmol/l), 3-hydroxybutyrate (mmol/l), free cholesterol to total lipids ratio in small HDL (%), citrate (mmol/l), free cholesterol to total lipids ratio in very large VLDL (%), free cholesterol to total lipids ratio in large HDL (%), triglycerides to total lipids ratio in medium HDL (%), phospholipids to total lipids ratio in small HDL (%), sphingomyelins (mmol/l) and albumin (signal area)

Calibration-in-the-large for the age-only risk score and risk scores 3, 4, and 5 (risk scores that performed better than age) is shown in Fig. 2. These results show the agreement between observed and predicted dementia rates to be similar for the four scores. The GND test suggested good calibration (all *p* > 0.05) for all scores but a poorer agreement between observed and predicted dementia rates was found in the 10th decile, suggesting poor prediction.

#### Additional analyses

Further analyses to evaluate the role of each metabolite in risk score 5 (the risk score with the best performance) suggested glucose and phospholipids to total lipids ratio in medium HDL (metabolites selected in the 100% and ≥90% in the selected models, respectively) to be important for the predictive accuracy of risk score 5 (Additional file 1: Table S7) as their exclusion had the greatest impact on all tests of predictive accuracy.

Adding ApoE e4 did not modify the pattern of results seen in the main analyses; risk scores 3, 4 and 5 had better c-statistic than the age and APOE only risk score (Additional file 1: Table S8); note that the c-statistic was higher when APOE was added to the risk score.

Further analyses to compare the predictive accuracy of our best-performing risk score (risk score 5) with set of metabolites identified in previous studies (Additional file 1: Table S9) showed risk score 5 to perform better, with or without age in the model (all *p*-values for difference in c-statistic using risk score 5 as reference <0.01).

## Discussion

We examined longitudinal associations between midlife serum metabolites and incident dementia over a follow-up of over 20 years in a large cohort of adults. The primary finding highlights the role of age; there was only a modest increase in predictive accuracy when metabolites selected using a machine learning approach were added to the prediction model containing age. Of the 233 metabolites examined, only glucose was associated with dementia after Bonferroni correction and in all models selected using the machine-learning approach. A further 14 metabolites were also identified by machine learning models. The contribution of these metabolites to dementia prediction was small, but it is worth noting that our approach required metabolites to improve predictive accuracy of a model containing age.

Pathophysiological hallmarks of Alzheimer’s disease are evident 15–20 years before the onset of clinical symptoms [31], making it important for studies on prevention to target risk factors in midlife. Accordingly, scalable biomarkers that allow early identification of persons at risk of dementia may allow therapeutic or lifestyle interventions to reduce future risk. They might also suggest the multiple mechanisms that underlie dementia. Our study on middle-aged adults (mean age at metabolite assessment of 55.8 years), followed for 21 years cannot address issues of causality but provides meaningful information on putative risk factors for dementia. Blood-based biomarkers have received considerable attention in recent years due to their minimally invasive nature. Previous studies have showed the usefulness of blood biomarkers such as tau phosphorylated at threonine 181 (p-tau181), neurofilament light (NfL) and glial fibrillary acidic protein (GFAP), in the diagnosis and prognosis of dementia, with comparable or even better performance than positron emission tomography (PET) and CSF biomarkers [32–34]. However, much of this research is on diagnostic rather than predictive biomarkers and does not use explicit criteria to select putative biomarkers.

The present study adds to current knowledge on predictors of dementia [17, 35] due to two novel features. One, we show the importance of explicit consideration of age in examination the predictive accuracy of risk scores for dementia. Age is both non-modifiable and an important risk factor for dementia, making it important for prediction risk scores of dementia to take age into account explicitly. A recent study based on 37 Alzheimer’s disease participants adopted the alternative approach by first entering metabolites in the prediction (area under the curve (AUC) 0.77) and then adding age (AUC improved to 0.81) [36]. Two, use of a machine learning approach, in our case elastic net regression, to identify relevant metabolites. The advantage of this method in comparison with correction for multiple testing lies in the efficient selection of highly correlated variables by regularization of both the number of selected metabolites and the effect size associated with the metabolites, [18] reducing the likelihood of overfitting [25]. In addition, the use of a repeated nested cross-validation procedure conferred a noteworthy element of stability to our results.

The lack of a widely accepted method for identifying metabolites relevant for dementia prediction, or for the construction of risk scores has led to inconsistent results in replication studies [37]. When cross-validation is used some authors have highlighted issues arising from the random partitioning of the dataset as results are inconsistent across the random samples [19, 38, 39]. Previous studies on metabolites have not considered this source of inconsistency [13, 40, 41]. We adopted an approach that allows circumvention of this limitation by repeating the cross-validation 100 times. Other studies using similar approaches, but are characterized by small sample sizes, cross-sectional design, or short follow-up—the AUC in these studies, without explicit consideration of age, was from 0.77 to 0.88 [36, 42].

A recent study on 38 metabolites in 1440 Chinese participants, mean age 70.7 years at baseline, used LASSO regression to identify 5 metabolites that predicted dementia (AUC 0.72) over a 5-year follow-up [13]. The authors used a cross-validation procedure for the estimation of AUC but the variation arising out of partitioning of the dataset was not considered. This was also the case for another study that found 10 plasma metabolites to predict a combined outcome of amnestic mild cognitive impairment or Alzheimer's disease with an AUC of 0.827 in the discovery sample and 0.77 in the validation sample [41]. However, these findings were not replicated in three subsequent studies which reported AUC between 0.395 and 0.642 [14, 15, 38]. These inconsistencies highlight the need to address the variation due to partitioning the dataset in cross-validated machine learning models.

Two previous meta-analyses, also including data from the Whitehall study, identified several metabolites to be associated with dementia after correction for multiple testing [11, 12], although the metabolites were not combined to examine their predictive performance nor was the role of age examined. The machine learning approach allowed us to identify 15 metabolites with higher predictive accuracy for incident dementia than that obtained using metabolites identified in the aforementioned studies (eTable 9). It is worth noting that although glucose was the only metabolite associated with dementia and selected in all the final models of our study, it was not identified in either of the previous studies.

Eleveated glucose is associated with increased risk of dementia, even among persons without diabetes [43]. The precise mechanisms underlying this association remain unclear but glucose neurotoxicity, hyperglycemia, insulin resistance and vascular injury are likely to be involved [44–46]. Higher creatinine signals poor kidney function, a risk factor for dementia [47, 48]. However, in our analyses and in those by Tynkkynen et al. [11] creatinine had an inverse association with dementia. The explanation for this unexpected association remains unclear. The results for albumin, an antioxidant, was similar to that in previous studies [49, 50] with higher serum albumin associated with lower dementia risk. As expected, [51, 52] higher concentrations of the amino acid alanine were associated with a lower risk of dementia, possibly due to antioxidant and anti-inflammatory pathways.

The results for lipids in our study varied depending on their fractions and combinations. VLDL is thought to increase dementia risk and HDL are associated with lower risk [53, 54]. We found ratios of triglycerides, phospholipids, and free cholesterol to total lipids in HDL and VLDL to be associated with dementia. While associations for free cholesterol and triglycerides ratios were in the expected direction, the unexpected finding was for phospholipids ratios where increments in HDL and VLDL were associated with higher and lower dementia risk, respectively. Phospholipids are main constituents of neuronal membrane structures and are the dominant HDL lipid component [54, 55], previous studies have also documented alterations in brain phospholipid concentrations of dementia patients [56, 57]. It is also thought that differing HDLs composition may exert distinct functions, possibly due to pathological and physiological processes [37, 54].

Our data show higher serum sphingomyelins to be associated with a lower risk of dementia. Previous studies have documented altered sphingomyelin metabolism in Alzheimer’s disease, with lower blood sphingomyelin levels in AD patients [36, 58]. Evidence for the remaining two metabolites, beta-hydroxybutyrate and citrate, is lacking and could not be compared to other studies.

The main strengths of the present study were the longitudinal design with a follow-up spanning a median 21 years, allowing a long separation between metabolite measurement and diagnosis of dementia to allow reverse causation bias to be minimized, the large sample size compared to previous studies and the use of a broadly validated platform for metabolite quantification. Our study also has several limitations. Lack of validation in an external cohort is an important limitation of machine learning studies. However, the methodological design of the present study made it possible to reduce overfitting due to use of repeated cross-validation. Absence of repeated measurement of metabolites did not allow us to examine how change in metabolites are associated with the risk of dementia. Cognitive status other than dementia diagnosis was not considered in the analyses as the focus of our analyses was dementia. It is possible that some participants had a level of cognitive impairment at baseline but this is unlikely to play a central role in our results on dementia. Sample storage might modify the lipoprotein composition, but these changes are minor compared to interindividual differences, and previous studies observed consistent results with differing duration of sample storage [59]. Although plasma was stored at −80 °C sample degradation is possible, but a recent publication suggests that even serum samples stored at −20 °C can be used in biomarker studies [60]. Although 233 metabolites were included in our study, the capture by NMR is still sparse compared to the entire serum metabolome, not allowing the identification of several metabolite subspecies (for example, subspecies of sphingomyelins) [21]. Ascertainment of dementia via linkage to electronic health records rather than clinical evaluation is likely to miss milder cases of dementia. However, this approach has the advantage of being able to include all participants in the analyses rather than only those who are seen during in-person in the ascertainment of dementia. The disadvantage is the lack of accurate data or missing data on dementia subtypes, not allowing us to examine whether the results are valid specifically for major types of dementia such as Alzheimer’s disease or vascular dementia. Furthermore, although there is emerging consensus on the biomarker-based definition of Alzheimer’s disease [5], biomarkers for other dementia subtypes remain to be identified. Given the uncertainty in the classification of dementia subtypes and the presence of vascular and metabolic dysfunctions in Alzheimer’s disease [3], our preference was to use all-cause dementia as the outcome.

## Conclusions

Given the increasing global burden of dementia and lack of effective treatment, it is important to identify individuals at higher risk of developing dementia to allow early interventions to prevent or delay its onset. Given the role of age for dementia, it is important that research on the identification of risk factors and biomarkers in the construction of risk scores explicitly consider age in the analyses. The evidence for glucose is robust in our results; further replication studies would allow conclusions to be drawn on other metabolites identified in our analyses. The improvement in predictive accuracy when metabolites were added to an age-only model was modest, making it urgent to identify other biomarkers for better prediction.

## Supplementary Information

**Additional file 1: Table S1**. Mean metabolite concentrations in 1997-1999 overall and as a function of dementia status at the end of follow-up (31^st^ March 2019). **Table S2**. The association between 1-SD increment in metabolite concentrations (separate models) and risk of dementia. **Table S3**. Association between 1-SD increment in metabolite concentrations and risk of dementia in analyses excluding participants with incomplete data on metabolites due to concentrations below the limit of quantification (*N*=5145). **Table S4**. Association between 1-SD increment in metabolite concentrations and risk of dementia including participants with incomplete data on metabolites due to outlier values (≥±9 SD) in metabolite concentrations (*N*=5446). **Table S5**. Elastic net penalized Cox regression with repeated nested cross-validation for incident dementia: results of 100 repetitions. **Table S6**. The beta coefficients from Cox regression used in the calculation of risk scores. **Table S7**. The contribution of metabolites, considered separately, to the predictive accuracy of risk score 5 (*N*=5374). **Table S8**. Predictive performance of risk scores for incident dementia; all models included ApoE (*N*=4494). **Table S9**. Comparison of the best risk score with metabolites previously identified using data from the Whitehall II cohort study (*N*=5374). **Figure S1**. Flow chart of sample selection.

## Data Availability

Data, protocols, and other metadata of the Whitehall II study are available to the scientific community either via the Whitehall II study data sharing portal (www.ucl.ac.uk/whitehallII/ data-sharing) or the DPUK platform (https://www.dementiasplatform.uk/).

## Abbreviations

CSF: Cerebrospinal fluid
NHS: National Health Service
NMR: Nuclear Magnetic Resonance
VLDL: Very low-density lipoproteins
IDL: Intermediate-density lipoproteins
LDL: Low-density lipoproteins
HDL: High-density lipoproteins
HES: Hospital Episode Statistics
MHSDS: Mental Health Services Data Set
SD: Standard deviation
AIC: Akaike information criterion
GND: Greenwood-Nam-D’Agostino
HR: Hazard ratio
p-tau181: Phosphorylated at threonine 181
NfL: Neurofilament light
GFAP: Glial fibrillary acidic protein
PET: Positron emission tomography
AUC: Area under the curve
