# Supplementary material for: Circulating serum metabolites as predictors of dementia: a machine learning approach in a 21-year follow-up of the Whitehall II cohort study
Source: BMC Med. 2022 Sep 27;20:334. doi: 10.1186/s12916-022-02519-6 (PMC9513883; doi:10.1186/s12916-022-02519-6)
Supplement: Supplementary file 1 — Additional file 1: Table S1. Mean metabolite concentrations in 1997-1999 overall and as a function of dementia status at the end of follow-up (31st March 2019). Table S2. The association between 1-SD increment in metabolite concentrations (separate models) and risk of dementia. Table S3. Association between 1-SD increment in metabolite concentrations and risk of dementia in analyses excluding participants with incomplete data on metabolites due to concentrations below the limit of quantification (N=5145). Table S4. Association between 1-SD increment in metabolite concentrations and risk of dementia including participants with incomplete data on metabolites due to outlier values (≥±9 SD) in metabolite concentrations (N=5446). Table S5. Elastic net penalized Cox regression with repeated nested cross-validation for incident dementia: results of 100 repetitions. Table S6. The beta coefficients from Cox regression used in the calculation of risk scores. Table S7. The contribution of metabolites, considered separately, to the predictive accuracy of risk score 5 (N=5374). Table S8. Predictive performance of risk scores for incident dementia; all models included ApoE (N=4494). Table S9. Comparison of the best risk score with metabolites previously identified using data from the Whitehall II cohort study (N=5374). Figure S1. Flow chart of sample selection. [file 12916_2022_2519_MOESM1_ESM.docx]

**Circulating serum metabolites as predictors of dementia: a machine learning approach in a 21-year follow-up of the Whitehall II cohort study**

Marcos D. Machado-Fragua^1*^

Benjamin Landré^1^

Mathilde Chen^1^

Aurore Fayosse^1^

Aline Dugravot^1^

Mika Kivimaki^2^

Séverine Sabia^1,2^

Archana Singh-Manoux^1,2^

**Supplementary data**

Table S1. Mean metabolite concentrations in 1997-1999 overall and as a function of dementia status at the end of follow-up (31^st^ March 2019).

Table S2. The association between 1-SD increment in metabolite concentrations (separate models) and risk of dementia.

Table S3. Association between 1-SD increment in metabolite concentrations and risk of dementia in analyses excluding participants with incomplete data on metabolites due to concentrations below the limit of quantification (N=5145).

Table S4. Association between 1-SD increment in metabolite concentrations and risk of dementia including participants with incomplete data on metabolites due to outlier values (≥±9 SD) in metabolite concentrations (N=5446).

Table S5. Elastic net penalized Cox regression with repeated nested cross-validation for incident dementia: results of 100 repetitions.

Table S6. The beta coefficients from Cox regression used in the calculation of risk scores.

Table S7. The contribution of metabolites, considered separately, to the predictive accuracy of risk score 5 (N=5374).

Table S8. Predictive performance of risk scores for incident dementia; all models included ApoE (N=4494).

Table S9. Comparison of the best risk score with metabolites previously identified using data from the Whitehall II cohort study (N=5374).

Figure S1. Flow chart of sample selection.

**Table S1. Mean metabolite concentrations in 1997-1999 overall, and as a function of dementia status at the end of follow-up (31^st^ March 2019).**

| **METABOLITE** | | | | | | | **Total population** | **Dementia** | |  |
| --- | --- | --- | --- | --- | --- | --- | --- | --- | --- | --- |
|  |  |  |  |  |  |  |  | **No** | **Yes** | **p-value^*^** |
| **Chylomicrons and extremely large VLDL** | | | | | | |  |  |  |  |
|  | | Concentration of chylomicrons and extremely large VLDL particles (mol/l) | | | | | 1x10^-10^ (7x10^-11^) | 1x10^-10^ (7x10^-11^) | 1x10^-10^ (7x10^-11^) | 0.48 |
|  | | Total lipids in chylomicrons and extremely large VLDL (mmol/l) | | | | | 0.025 (0.014) | 0.025 (0.014) | 0.025 (0.016) | 0.46 |
|  | | Phospholipids in chylomicrons and extremely large VLDL (mmol/l) | | | | | 0.003 (0.002) | 0.003 (0.002) | 0.003 (0.002) | 0.28 |
|  | | Total cholesterol in chylomicrons and extremely large VLDL (mmol/l) | | | | | 0.004 (0.002) | 0.004 (0.002) | 0.004 (0.003) | 0.25 |
|  | | Cholesterol esters in chylomicrons and extremely large VLDL (mmol/l) | | | | | 0.003 (0.001) | 0.003 (0.001) | 0.003 (0.001) | 0.28 |
|  | | Free cholesterol in chylomicrons and extremely large VLDL (mmol/l) | | | | | 0.002 (0.001) | 0.002 (0.001) | 0.002 (0.001) | 0.22 |
|  | | Triglycerides in chylomicrons and extremely large VLDL (mmol/l) | | | | | 0.018 (0.010) | 0.018 (0.010) | 0.018 (0.011) | 0.57 |
| **Very large VLDL** | | | | | | |  |  |  |  |
|  | | | | Concentration of very large VLDL particles (mol/l) | | | 5x10^-10^ (3x10^-10^) | 5x10^-10^ (4x10^-10^) | 5x10^-10^ (4x10^-10^) | 0.24 |
|  | | | | Total lipids in very large VLDL (mmol/l) | | | 0.046 (0.036) | 0.046 (0.036) | 0.046 (0.039) | 0.23 |
|  | | | | Phospholipids in very large VLDL (mmol/l) | | | 0.008 (0.006) | 0.008 (0.006) | 0.008 (0.007) | 0.31 |
|  | | | | Total cholesterol in very large VLDL (mmol/l) | | | 0.011 (0.007) | 0.011 (0.007) | 0.011 (0.008) | 0.15 |
|  | | | | Cholesterol esters in very large VLDL (mmol/l) | | | 0.006 (0.004) | 0.006 (0.004) | 0.006 (0.004) | 0.07 |
|  | | | | Free cholesterol in very large VLDL (mmol/l) | | | 0.005 (0.003) | 0.005 (0.003) | 0.005 (0.004) | 0.34 |
|  | | | | Triglycerides in very large VLDL (mmol/l) | | | 0.027 (0.023) | 0.027 (0.023) | 0.027 (0.025) | 0.26 |
| **Large VLDL** | | | | | | |  |  |  |  |
|  | | | Concentration of large VLDL particles (mol/l) | | | | 4X10^-9^ (2x10^-9^) | 3X10^-9^ (2x10^-9^) | 4X10^-9^ (2x10^-9^) | 0.50 |
|  | | | Total lipids in large VLDL (mmol/l) | | | | 0.202 (0.132) | 0.202 (0.132) | 0.204 (0.145) | 0.48 |
|  | | | Phospholipids in large VLDL (mmol/l) | | | | 0.037 (0.024) | 0.037 (0.024) | 0.037 (0.026) | 0.62 |
|  | | | Total cholesterol in large VLDL (mmol/l) | | | | 0.052 (0.030) | 0.052 (0.030) | 0.051 (0.033) | 0.26 |
|  | | | Cholesterol esters in large VLDL (mmol/l) | | | | 0.031 (0.015) | 0.031 (0.015) | 0.030 (0.016) | 0.18 |
|  | | | Free cholesterol in large VLDL (mmol/l) | | | | 0.021 (0.015) | 0.021 (0.015) | 0.021 (0.017) | 0.39 |
|  | | | Triglycerides in large VLDL (mmol/l) | | | | 0.114 (0.079) | 0.114 (0.078) | 0.115 (0.086) | 0.56 |
| **Medium VLDL** | | | | | | |  |  |  |  |
|  | Concentration of medium VLDL particles (mol/l) | | | | | | 2x10^-8^ (7x10^-9^) | 2x10^-8^ (7x10^-9^) | 2x10^-8^ (8x10^-9^) | 0.73 |
|  | Total lipids in medium VLDL (mmol/l) | | | | | | 0.547 (0.239) | 0.547 (0.238) | 0.550 (0.256) | 0.75 |
|  | Phospholipids in medium VLDL (mmol/l) | | | | | | 0.111 (0.046) | 0.111 (0.046) | 0.112 (0.050) | 0.87 |
|  | Total cholesterol in medium VLDL (mmol/l) | | | | | | 0.174 (0.065) | 0.174 (0.065) | 0.175 (0.070) | 0.92 |
|  | Cholesterol esters in medium VLDL (mmol/l) | | | | | | 0.110 (0.035) | 0.110 (0.035) | 0.111 (0.038) | 0.99 |
|  | Free cholesterol in medium VLDL (mmol/l) | | | | | | 0.064 (0.031) | 0.064 (0.031) | 0.064 (0.033) | 0.81 |
| **METABOLITE** | | | | | | | **Total population** | **Dementia** | |  |
|  |  |  |  |  |  |  |  | **No** | **Yes** | **p-value^*^** |
| **Medium VLDL** | | | | | | |  |  |  |  |
|  | Triglycerides in medium VLDL (mmol/l) | | | | | | 0.261 (0.129) | 0.261 (0.128) | 0.262 (0.138) | 0.65 |
| **Small VLDL** | | | | | | |  |  |  |  |
|  | | | | Concentration of small VLDL particles (mol/l) | | | 3x10^-8^ (1x10^-8^) | 3x10^-8^ (1x10^-8^) | 3x10^-8^ (1x10^-8^) | 0.58 |
|  | | | | Total lipids in small VLDL (mmol/l) | | | 0.676 (0.196) | 0.676 (0.196) | 0.686 (0.206) | 0.50 |
|  | | | | Phospholipids in small VLDL (mmol/l) | | | 0.157 (0.042) | 0.157 (0.042) | 0.159 (0.045) | 0.59 |
|  | | | | Total cholesterol in small VLDL (mmol/l) | | | 0.276 (0.070) | 0.275 (0.070) | 0.281 (0.072) | 0.11 |
|  | | | | Cholesterol esters in small VLDL (mmol/l) | | | 0.179 (0.045) | 0.179 (0.045) | 0.184 (0.045) | 0.06 |
|  | | | | Free cholesterol in small VLDL (mmol/l) | | | 0.096 (0.028) | 0.096 (0.028) | 0.098 (0.029) | 0.45 |
|  | | | | Triglycerides in small VLDL (mmol/l) | | | 0.244 (0.091) | 0.244 (0.091) | 0.246 (0.097) | 0.93 |
| **Very small VLDL** | | | | | | |  |  |  |  |
|  | | | | | | Concentration of very small VLDL particles (mol/l) | 5x10^-8^ (1x10^-8^) | 5x10^-8^ (1x10^-8^) | 5x10^-8^ (1x10^-8^) | **0.01** |
|  | | | | | | Total lipids in very small VLDL (mmol/l) | 0.651 (0.133) | 0.650 (0.133) | 0.668 (0.135) | **0.01** |
|  | | | | | | Phospholipids in very small VLDL (mmol/l) | 0.192 (0.041) | 0.192 (0.040) | 0.197 (0.042) | **0.01** |
|  | | | | | | Total cholesterol in very small VLDL (mmol/l) | 0.335 (0.067) | 0.335 (0.067) | 0.345 (0.066) | **0.008** |
|  | | | | | | Cholesterol esters in very small VLDL (mmol/l) | 0.231 (0.045) | 0.230 (0.045) | 0.237 (0.044) | **0.004** |
|  | | | | | | Free cholesterol in very small VLDL (mmol/l) | 0.105 (0.022) | 0.105 (0.022) | 0.107 (0.026) | **0.04** |
|  | | | | | | Triglycerides in very small VLDL (mmol/l) | 0.123 (0.039) | 0.123 (0.038) | 0.126 (0.042) | 0.36 |
| **IDL** | | | | | | |  |  |  |  |
|  | | | | Concentration of IDL particles (mol/l) | | | 1x10^-7^ (3x10^-8^) | 1x10^-7^ (3x10^-8^) | 1x10^-7^ (3x10^-8^) | **0.01** |
|  | | | | Total lipids in IDL (mmol/l) | | | 1.35 (0.27) | 1.34 (0.27) | 1.38 (0.28) | **0.01** |
|  | | | | Phospholipids in IDL (mmol/l) | | | 0.354 (0.066) | 0.354 (0.066) | 0.363 (0.070) | **0.02** |
|  | | | | Total cholesterol in IDL (mmol/l) | | | 0.862 (0.189) | 0.860 (0.188) | 0.884 (0.198) | **0.03** |
|  | | | | Cholesterol esters in IDL (mmol/l) | | | 0.615 (0.136) | 0.614 (0.136) | 0.632 (0.143) | **0.03** |
|  | | | | Free cholesterol in IDL (mmol/l) | | | 0.246 (0.053) | 0.246 (0.053) | 0.253 (0.056) | **0.02** |
|  | | | | Triglycerides in IDL (mmol/l) | | | 0.131 (0.034) | 0.131 (0.034) | 0.135 (0.036) | **0.01** |
| **Large LDL** | | | | | | |  |  |  |  |
|  | | | Concentration of large LDL particles (mol/l) | | | | 2x10^-7^ (4x10^-8^) | 2x10^-7^ (4x10^-8^) | 2x10^-7^ (5x10^-8^) | **0.01** |
|  | | | Total lipids in large LDL (mmol/l) | | | | 1.49 (0.31) | 1.48 (0.31) | 1.53 (0.33) | **0.02** |
|  | | | Phospholipids in large LDL (mmol/l) | | | | 0.368 (0.065) | 0.368 (0.065) | 0.376 (0.069) | **0.03** |
|  | | | Total cholesterol in large LDL (mmol/l) | | | | 1.01 (0.23) | 1.01 (0.23) | 1.04 (0.25) | **0.03** |
|  | | | Cholesterol esters in large LDL (mmol/l) | | | | 0.725 (0.175) | 0.723 (0.174) | 0.745 (0.185) | **0.03** |
|  | | | Free cholesterol in large LDL (mmol/l) | | | | 0.284 (0.057) | 0.283 (0.057) | 0.291 (0.061) | **0.02** |
| **METABOLITE** | | | | | | | **Total population** | **Dementia** | |  |
|  |  |  |  |  |  |  |  | **No** | **Yes** | **p-value^*^** |
| **Large LDL** | | | | | | |  |  |  |  |
|  | | | Triglycerides in large LDL (mmol/l) | | | | 0.111 (0.027) | 0.110 (0.027) | 0.115 (0.028) | **0.001** |
| **Medium LDL** | | | | | | |  |  |  |  |
|  | Concentration of medium LDL particles (mol/l) | | | | | | 2x10^-7^ (4x10^-8^) | 2x10^-7^ (4x10^-8^) | 2x10^-7^ (4x10^-8^) | **0.02** |
|  | Total lipids in medium LDL (mmol/l) | | | | | | 0.826 (0.183) | 0.824 (0.183) | 0.847 (0.193) | **0.03** |
|  | Phospholipids in medium LDL (mmol/l) | | | | | | 0.221 (0.038) | 0.220 (0.038) | 0.226 (0.040) | **0.02** |
|  | | | | Total cholesterol in medium LDL (mmol/l) | | | 0.555 (0.140) | 0.554 (0.139) | 0.570 (0.148) | 0.06 |
|  | | | | Cholesterol esters in medium LDL (mmol/l) | | | 0.402 (0.114) | 0.401 (0.114) | 0.413 (0.121) | 0.08 |
|  | | | | Free cholesterol in medium LDL (mmol/l) | | | 0.153 (0.026) | 0.153 (0.026) | 0.157 (0.028) | **0.02** |
|  | | | | Triglycerides in medium LDL (mmol/l) | | | 0.050 (0.013) | 0.050 (0.013) | 0.052 (0.013) | **0.001** |
| **Small LDL** | | | | | | |  |  |  |  |
|  | | | | | Concentration of small LDL particles (mol/l) | | 2x10^-7^ (4x10^-8^) | 2x10^-7^ (4x10^-8^) | 2x10^-7^ (4x10^-8^) | **0.03** |
|  | | | | | Total lipids in small LDL (mmol/l) | | 0.521 (0.109) | 0.520 (0.108) | 0.534 (0.115) | **0.03** |
|  | | | | | Phospholipids in small LDL (mmol/l) | | 0.154 (0.023) | 0.154 (0.023) | 0.157 (0.024) | **0.03** |
|  | | | | | Total cholesterol in small LDL (mmol/l) | | 0.335 (0.083) | 0.335 (0.082) | 0.345 (0.087) | 0.05 |
|  | | | | | Cholesterol esters in small LDL (mmol/l) | | 0.242 (0.068) | 0.242 (0.068) | 0.249 (0.072) | 0.06 |
|  | | | | | Free cholesterol in small LDL (mmol/l) | | 0.093 (0.016) | 0.093 (0.016) | 0.095 (0.016) | **0.02** |
|  | | | | | Triglycerides in small LDL (mmol/l) | | 0.031 (0.009) | 0.031 (0.009) | 0.032 (0.010) | **0.03** |
| **Very large HDL** | | | | | | |  |  |  |  |
|  | | Concentration of very large HDL particles (mol/l) | | | | | 5x10^-7^ (2x10^-7^) | 5x10^-7^ (2x10^-7^) | 5x10^-7^ (2x10^-7^) | **0.002** |
|  | | Total lipids in very large HDL (mmol/l) | | | | | 0.470 (0.183) | 0.468 (0.183) | 0.490 (0.171) | **0.002** |
|  | | Phospholipids in very large HDL (mmol/l) | | | | | 0.232 (0.098) | 0.231 (0.098) | 0.242 (0.089) | **0.004** |
|  | | Total cholesterol in very large HDL (mmol/l) | | | | | 0.224 (0.085) | 0.223 (0.085) | 0.234 (0.083) | **0.002** |
|  | | Cholesterol esters in very large HDL (mmol/l) | | | | | 0.159 (0.060) | 0.159 (0.061) | 0.166 (0.059) | **0.002** |
|  | | Free cholesterol in very large HDL (mmol/l) | | | | | 0.064 (0.025) | 0.064 (0.025) | 0.068 (0.024) | **0.001** |
|  | | Triglycerides in very large HDL (mmol/l) | | | | | 0.014 (0.006) | 0.014 (0.006) | 0.014 (0.006) | 0.51 |
| **Large HDL** | | | | | | |  |  |  |  |
|  | | Concentration of large HDL particles (mol/l) | | | | | 1x10^-6^ (5x10^-7^) | 1x10^-6^ (5x10^-7^) | 1x10^-6^ (5x10^-7^) | 0.37 |
|  | | Total lipids in large HDL (mmol/l) | | | | | 0.800 (0.290) | 0.800 (0.292) | 0.800 (0.251) | 0.38 |
|  | | Phospholipids in large HDL (mmol/l) | | | | | 0.395 (0.130) | 0.395 (0.131) | 0.395 (0.111) | 0.44 |
|  | | Total cholesterol in large HDL (mmol/l) | | | | | 0.378 (0.152) | 0.378 (0.153) | 0.378 (0.134) | 0.37 |
|  | | Cholesterol esters in large HDL (mmol/l) | | | | | 0.295 (0.115) | 0.295 (0.116) | 0.295 (0.101) | 0.35 |
|  | | Free cholesterol in large HDL (mmol/l) | | | | | 0.083 (0.037) | 0.083 (0.037) | 0.083 (0.032) | 0.41 |
| **METABOLITE** | | | | | | | **Total population** | **Dementia** | |  |
|  |  |  |  |  |  |  |  | **No** | **Yes** | **p-value^*^** |
| **Large HDL** | | | | | | |  |  |  |  |
|  | | Triglycerides in large HDL (mmol/l) | | | | | 0.027 (0.011) | 0.027 (0.012) | 0.027 (0.010) | 0.29 |
| **Medium HDL** | | | | | | |  |  |  |  |
|  | | | Concentration of medium HDL particles (mol/l) | | | | 2x10^-6^ (4x10^-7^) | 2x10^-6^ (4x10^-7^) | 2x10^-6^ (4x10^-7^) | 0.24 |
|  | | | Total lipids in medium HDL (mmol/l) | | | | 0.992 (0.179) | 0.992 (0.181) | 0.977 (0.162) | 0.23 |
|  | | | Phospholipids in medium HDL (mmol/l) | | | | 0.453 (0.081) | 0.453 (0.081) | 0.449 (0.074) | 0.57 |
|  | | | Total cholesterol in medium HDL (mmol/l) | | | | 0.484 (0.098) | 0.485 (0.098) | 0.474 (0.087) | 0.08 |
|  | | | Cholesterol esters in medium HDL (mmol/l) | | | | 0.391 (0.077) | 0.392 (0.078) | 0.382 (0.068) | **0.04** |
|  | | | Free cholesterol in medium HDL (mmol/l) | | | | 0.093 (0.021) | 0.093 (0.021) | 0.092 (0.019) | 0.42 |
|  | | | Triglycerides in medium HDL (mmol/l) | | | | 0.054 (0.012) | 0.054 (0.012) | 0.054 (0.013) | 0.57 |
| **Small HDL** | | | | | | |  |  |  |  |
|  | | | Concentration of small HDL particles (mol/l) | | | | 5x10^-6^ (5x10^-7^) | 5x10^-6^ (5x10^-7^) | 5x10^-6^ (5x10^-7^) | 0.30 |
|  | | | Total lipids in small HDL (mmol/l) | | | | 1.09 (0.12) | 1.09 (0.12) | 1.08 (0.12) | 0.29 |
|  | | | Phospholipids in small HDL (mmol/l) | | | | 0.620 (0.076) | 0.620 (0.076) | 0.612 (0.076) | 0.09 |
|  | | | Total cholesterol in small HDL (mmol/l) | | | | 0.427 (0.056) | 0.427 (0.056) | 0.427 (0.057) | 0.89 |
|  | | | Cholesterol esters in small HDL (mmol/l) | | | | 0.312 (0.048) | 0.312 (0.048) | 0.314 (0.049) | 0.75 |
|  | | | Free cholesterol in small HDL (mmol/l) | | | | 0.114 (0.016) | 0.114 (0.016) | 0.113 (0.016) | 0.17 |
|  | | | Triglycerides in small HDL (mmol/l) | | | | 0.044 (0.011) | 0.044 (0.011) | 0.045 (0.012) | 0.65 |
| **Chylomicrons and extremely large VLDL ratios** | | | | | | |  |  |  |  |
|  | | Phospholipids to total lipids ratio in chylomicrons and extremely large VLDL (%) | | | | | 10.9 (1.1) | 10.9 (1.1) | 10.8 (1.1) | **0.003** |
|  | | Total cholesterol to total lipids ratio in chylomicrons and extremely large VLDL (%) | | | | | 17.4 (1.5) | 17.4 (1.6) | 17.2 (1.5) | **0.005** |
|  | | Cholesterol esters to total lipids ratio in chylomicrons and extremely large VLDL (%) | | | | | 10.8 (1.3) | 10.8 (1.3) | 10.7 (1.3) | 0.24 |
|  | | Free cholesterol to total lipids ratio in chylomicrons and extremely large VLDL (%) | | | | | 6.6 (1.0) | 6.6 (1.0) | 6.5 (1.1) | **0.009** |
|  | | Triglycerides to total lipids ratio in chylomicrons and extremely large VLDL (%) | | | | | 71.6 (2.3) | 71.6 (2.3) | 72.0 (2.2) | **0.001** |
| **Very large VLDL ratios** | | | | | | |  |  |  |  |
|  | | | Phospholipids to total lipids ratio in very large VLDL (%) | | | | 17.7 (3.5) | 17.7 (3.4) | 18.0 (3.8) | 0.11 |
|  | | | Total cholesterol to total lipids ratio in very large VLDL (%) | | | | 26.8 (7.7) | 26.8 (7.6) | 27.1 (8.4) | 0.64 |
|  | | | Cholesterol esters to total lipids ratio in very large VLDL (%) | | | | 14.2 (4.9) | 14.2 (4.9) | 14.0 (5.1) | 0.05 |
|  | | | Free cholesterol to total lipids ratio in very large VLDL (%) | | | | 12.6 (4.3) | 12.6 (4.2) | 13.1 (5.2) | 0.15 |
|  | | | Triglycerides to total lipids ratio in very large VLDL (%) | | | | 55.5 (9.5) | 55.5 (9.5) | 55.0 (10.6) | 0.93 |
| **Large VLDL ratios** | | | | | | |  |  |  |  |
|  | | | | Phospholipids to total lipids ratio in large VLDL (%) | | | 18.3 (1.0) | 18.2 (1.0) | 18.4 (1.2) | **0.001** |
|  | | | | Total cholesterol to total lipids ratio in large VLDL (%) | | | 27.0 (4.0) | 27.0 (4.0) | 26.6 (3.7) | 0.09 |
| **METABOLITE** | | | | | | | **Total population** | **Dementia** | |  |
|  |  |  |  |  |  |  |  | **No** | **Yes** | **p-value^*^** |
| **Large VLDL ratios** | | | | | | |  |  |  |  |
|  | | | | Cholesterol esters to total lipids ratio in large VLDL (%) | | | 17.2 (4.5) | 17.2 (4.5) | 17.0 (4.3) | 0.35 |
|  | | | | Free cholesterol to total lipids ratio in large VLDL (%) | | | 9.7 (1.7) | 9.7 (1.7) | 9.6 (1.7) | 0.22 |
|  | | | | Triglycerides to total lipids ratio in large VLDL (%) | | | 54.8 (4.3) | 54.8 (4.3) | 55.0 (4.1) | 0.42 |
| **Medium VLDL ratios** | | | | | | |  |  |  |  |
|  | | | | | Phospholipids to total lipids ratio in medium VLDL (%) | | 20.6 (0.7) | 20.5 (0.6) | 20.6 (0.7) | **0.03** |
|  | | | | | Total cholesterol to total lipids ratio in medium VLDL (%) | | 32.9 (3.7) | 32.9 (3.7) | 33.0 (3.6) | 0.39 |
|  | | | | | Cholesterol esters to total lipids ratio in medium VLDL (%) | | 21.5 (4.0) | 21.5 (4.0) | 21.5 (4.0) | 0.48 |
|  | | | | | Free cholesterol to total lipids ratio in medium VLDL (%) | | 11.4 (0.8) | 11.4 (0.8) | 11.4 (0.8) | 0.66 |
|  | | | | | Triglycerides to total lipids ratio in medium VLDL (%) | | 46.6 (4.2) | 46.6 (4.2) | 46.4 (4.2) | 0.26 |
| **Small VLDL ratios** | | | | | | |  |  |  |  |
|  | | | Phospholipids to total lipids ratio in small VLDL (%) | | | | 23.3 (1.0) | 23.3 (1.0) | 23.3 (0.9) | 0.29 |
|  | | | Total cholesterol to total lipids ratio in small VLDL (%) | | | | 41.4 (4.1) | 41.3 (4.1) | 41.7 (4.4) | 0.08 |
|  | | | Cholesterol esters to total lipids ratio in small VLDL (%) | | | | 27.1 (4.0) | 27.1 (4.0) | 27.5 (4.3) | 0.09 |
|  | | | | | Free cholesterol to total lipids ratio in small VLDL (%) | | 14.2 (0.5) | 14.2 (0.5) | 14.2 (0.5) | 0.29 |
|  | | | | | Triglycerides to total lipids ratio in small VLDL (%) | | 35.3 (4.3) | 35.4 (4.3) | 35.0 (4.5) | 0.10 |
| **Very small VLDL ratios** | | | | | | |  |  |  |  |
|  | | | Phospholipids to total lipids ratio in very small VLDL (%) | | | | 29.5 (1.5) | 29.5 (1.5) | 29.5 (1.5) | 0.85 |
|  | | | Total cholesterol to total lipids ratio in very small VLDL (%) | | | | 51.7 (3.0) | 51.7 (3.0) | 51.8 (3.2) | 0.31 |
|  | | | Cholesterol esters to total lipids ratio in very small VLDL (%) | | | | 35.6 (2.8) | 35.6 (2.8) | 35.8 (2.9) | 0.14 |
|  | | | Free cholesterol to total lipids ratio in very small VLDL (%) | | | | 16.1 (0.8) | 16.1 (0.8) | 16.0 (0.9) | 0.26 |
|  | | | Triglycerides to total lipids ratio in very small VLDL (%) | | | | 18.8 (3.7) | 18.8 (3.7) | 18.7 (4.0) | 0.24 |
| **IDL ratios** | | | | | | |  |  |  |  |
|  | | | | | Phospholipids to total lipids ratio in IDL (%) | | 26.4 (0.7) | 26.4 (0.7) | 26.3 (0.6) | **0.03** |
|  | | | | | Total cholesterol to total lipids ratio in IDL (%) | | 63.7 (2.3) | 63.7 (2.3) | 63.7 (2.5) | 0.68 |
|  | | | | | Cholesterol esters to total lipids ratio in IDL (%) | | 45.5 (2.0) | 45.5 (2.0) | 45.5 (2.0) | 0.84 |
|  | | | | | Free cholesterol to total lipids ratio in IDL (%) | | 18.2 (0.8) | 18.2 (0.8) | 18.2 (0.8) | 0.60 |
|  | | | | | Triglycerides to total lipids ratio in IDL (%) | | 9.9 (2.3) | 9.8 (2.3) | 10.0 (2.6) | 0.90 |
| **Large LDL ratios** | | | | | | |  |  |  |  |
|  | | | | Phospholipids to total lipids ratio in large LDL (%) | | | 24.9 (1.0) | 24.9 (1.0) | 24.8 (1.0) | **0.002** |
|  | | | | Total cholesterol to total lipids ratio in large LDL (%) | | | 67.5 (2.2) | 67.5 (2.2) | 67.5 (2.3) | 0.65 |
|  | | | | Cholesterol esters to total lipids ratio in large LDL (%) | | | 48.4 (2.1) | 48.4 (2.1) | 48.4 (2.2) | 0.57 |
|  | | | | Free cholesterol to total lipids ratio in large LDL (%) | | | 19.1 (0.6) | 19.1 (0.6) | 19.1 (0.7) | 0.65 |
| **METABOLITE** | | | | | | | **Total population** | **Dementia** | |  |
|  |  |  |  |  |  |  |  | **No** | **Yes** | **p-value^*^** |
| **Large LDL ratios** | | | | | | |  |  |  |  |
|  | | | | Triglycerides to total lipids ratio in large LDL (%) | | | 7.6 (1.7) | 7.6 (1.7) | 7.7 (1.8) | 0.38 |
| **Medium LDL ratios** | | | | | | |  |  |  |  |
|  | | | | | | Phospholipids to total lipids ratio in medium LDL (%) | 27.1 (2.2) | 27.1 (2.2) | 27.0 (2.3) | 0.34 |
|  | | | | | | Total cholesterol to total lipids ratio in medium LDL (%) | 66.7 (3.3) | 66.7 (3.3) | 66.7 (3.4) | 0.97 |
|  | | | | | | Cholesterol esters to total lipids ratio in medium LDL (%) | 47.9 (4.2) | 47.9 (4.2) | 48.0 (4.4) | 0.84 |
|  | | | | | | Free cholesterol to total lipids ratio in medium LDL (%) | 18.8 (1.2) | 18.8 (1.2) | 18.7 (1.4) | 0.20 |
|  | | | | | | Triglycerides to total lipids ratio in medium LDL (%) | 6.2 (1.5) | 6.1 (1.5) | 6.3 (1.5) | 0.18 |
| **Small LDL ratios** | | | | | | |  |  |  |  |
|  | | | | | Phospholipids to total lipids ratio in small LDL (%) | | 30.1 (2.7) | 30.1 (2.7) | 29.9 (2.8) | 0.09 |
|  | | | | | Total cholesterol to total lipids ratio in small LDL (%) | | 63.8 (3.8) | 63.8 (3.8) | 63.9 (3.8) | 0.37 |
|  | | | | | Cholesterol esters to total lipids ratio in small LDL (%) | | 45.7 (4.7) | 45.7 (4.7) | 45.9 (4.8) | 0.34 |
|  | | | | | Free cholesterol to total lipids ratio in small LDL (%) | | 18.1 (1.1) | 18.1 (1.1) | 18.0 (1.2) | 0.18 |
|  | | | | | Triglycerides to total lipids ratio in small LDL (%) | | 6.1 (1.5) | 6.1 (1.5) | 6.2 (1.6) | 0.93 |
| **Very large HDL ratios** | | | | | | |  |  |  |  |
|  | | | | | Phospholipids to total lipids ratio in very large HDL (%) | | 48.9 (4.9) | 48.9 (4.9) | 49.2 (4.4) | 0.90 |
|  | | | | | Total cholesterol to total lipids ratio in very large HDL (%) | | 47.8 (4.4) | 47.9 (4.4) | 47.6 (4.4) | 0.79 |
|  | | | | | Cholesterol esters to total lipids ratio in very large HDL (%) | | 34.1 (4.0) | 34.2 (4.1) | 33.9 (3.9) | 0.74 |
|  | | | | | Free cholesterol to total lipids ratio in very large HDL (%) | | 13.7 (1.3) | 13.7 (1.3) | 13.8 (1.3) | 0.07 |
|  | | | | | Triglycerides to total lipids ratio in very large HDL (%) | | 3.2 (1.4) | 3.2 (1.4) | 3.1 (1.5) | 0.08 |
| **Large HDL ratios** | | | | | | |  |  |  |  |
|  | | | | | Phospholipids to total lipids ratio in large HDL (%) | | 50.0 (2.4) | 50.0 (2.4) | 49.8 (2.4) | 0.16 |
|  | | | | | Total cholesterol to total lipids ratio in large HDL (%) | | 46.6 (2.5) | 46.6 (2.5) | 46.7 (2.5) | 0.17 |
|  | | | | | Cholesterol esters to total lipids ratio in large HDL (%) | | 36.5 (1.7) | 36.5 (1.7) | 36.6 (1.7) | 0.15 |
|  | | | | | Free cholesterol to total lipids ratio in large HDL (%) | | 10.1 (1.0) | 10.1 (1.0) | 10.1 (1.0) | 0.31 |
|  | | | | | Triglycerides to total lipids ratio in large HDL (%) | | 3.4 (1.0) | 3.4 (1.0) | 3.4 (1.0) | 0.64 |
| **Medium HDL ratios** | | | | | | |  |  |  |  |
|  | | | | | | Phospholipids to total lipids ratio in medium HDL (%) | 45.7 (1.2) | 45.7 (1.2) | 46.0 (1.3) | **0.003** |
|  | | | | | | Total cholesterol to total lipids ratio in medium HDL (%) | 48.7 (2.2) | 48.7 (2.2) | 48.4 (2.3) | **0.03** |
|  | | | | | | Cholesterol esters to total lipids ratio in medium HDL (%) | 39.4 (2.1) | 39.4 (2.1) | 39.1 (2.2) | **0.02** |
|  | | | | | | Free cholesterol to total lipids ratio in medium HDL (%) | 9.3 (0.5) | 9.3 (0.5) | 9.3 (0.5) | 0.46 |
|  | | | | | | Triglycerides to total lipids ratio in medium HDL (%) | 5.6 (1.3) | 5.6 (1.3) | 5.6 (1.3) | 0.53 |

| **METABOLITE** | | | | **Total population** | **Dementia** | |  |
| --- | --- | --- | --- | --- | --- | --- | --- |
|  |  |  |  |  | **No** | **Yes** | **p-value^*^** |
| **Small HDL ratios** | | | |  |  |  |  |
|  | | | Phospholipids to total lipids ratio in small HDL (%) | 56.8 (2.8) | 56.8 (2.8) | 56.5 (2.9) | **0.04** |
|  | | | Total cholesterol to total lipids ratio in small HDL (%) | 39.1 (3.2) | 39.1 (3.2) | 39.4 (3.4) | 0.13 |
|  | | | Cholesterol esters to total lipids ratio in small HDL (%) | 28.7 (3.5) | 28.7 (3.5) | 29.0 (3.8) | 0.10 |
|  | | | Free cholesterol to total lipids ratio in small HDL (%) | 10.5 (0.5) | 10.5 (0.5) | 10.4 (0.5) | 0.08 |
|  | | | Triglycerides to total lipids ratio in small HDL (%) | 4.0 (1.0) | 4.0 (1.0) | 4.1 (1.0) | 0.36 |
| **Lipoprotein particle sizes** | | | |  |  |  |  |
|  | | Mean diameter for VLDL particles (nm) | | 33.9 (0.9) | 35.9 (0.9) | 35.8 (0.9) | 0.10 |
|  | | Mean diameter for LDL particles (nm) | | 23.7 (0.1) | 23.7 (0.1) | 23.7 (0.1) | 0.59 |
|  | | Mean diameter for HDL particles (nm) | | 10.0 (0.2) | 10.0 (0.2) | 10.0 (0.2) | 0.05 |
| **Cholesterol related metabolites** | | | |  |  |  |  |
|  | | Serum total cholesterol (mmol/l) | | 5.1 (0.9) | 5.1 (0.9) | 5.2 (0.9) | 0.05 |
|  | | Total cholesterol in VLDL (mmol/l) | | 0.852 (0.217) | 0.851 (0.217) | 0.867 (0.217) | 0.21 |
|  | | Remnant cholesterol (non-HDL, non-LDL -cholesterol) (mmol/l) | | 1.7 (0.4) | 1.7 (0.4) | 1.8 (0.4) | 0.05 |
|  | | Total cholesterol in LDL (mmol/l) | | 1.9 (0.5) | 1.9 (0.5) | 2.0 (0.5) | **0.04** |
|  | | Total cholesterol in HDL (mmol/l) | | 1.5 (0.3) | 1.5 (0.3) | 1.5 (0.3) | 0.47 |
|  | | Total cholesterol in HDL2 (mmol/l) | | 1.0 (0.3) | 1.0 (0.3) | 1.0 (0.3) | 0.60 |
|  | | Total cholesterol in HDL3 (mmol/l) | | 0.481 (0.039) | 0.481 (0.039) | 0.483 (0.037) | 0.09 |
|  | | Esterified cholesterol (mmol/l) | | 3.7 (0.6) | 3.7 (0.6) | 3.8 (0.7) | 0.07 |
|  | | Free cholesterol (mmol/l) | | 1.4 (0.2) | 1.4 (0.2) | 1.5 (0.3) | **0.02** |
| **Lipids related metabolites** | | | |  |  |  |  |
|  | Serum total triglycerides (mmol/l) | | | 1.3 (0.4) | 1.2 (0.4) | 1.3 (0.5) | 0.82 |
|  | | | Triglycerides in VLDL (mmol/l) | 0.788 (0.361) | 0.788 (0.359) | 0.794 (0.390) | 0.77 |
|  | | | Triglycerides in LDL (mmol/l) | 0.192 (0.048) | 0.191 (0.048) | 0.199 (0.051) | **0.002** |
|  | | | Triglycerides in HDL (mmol/l) | 0.139 (0.030) | 0.139 (0.030) | 0.140 (0.032) | 0.71 |
|  | | | Diacylglycerol (mmol/l) | 0.020 (0.014) | 0.020 (0.014) | 0.021 (0.015) | 0.14 |
|  | | | Ratio of diacylglycerol to triglycerides | 0.015 (0.008) | 0.015 (0.008) | 0.016 (0.008) | 0.02 |
|  | | | Total phosphoglycerides (mmol/l) | 2.1 (0.3) | 2.1 (0.4) | 2.1 (0.3) | 0.46 |
|  | | | Ratio of triglycerides to phosphoglycerides | 0.588 (0.223) | 0.589 (0.223) | 0.584 (0.224) | 0.49 |
|  | | | Phosphatidylcholine and other cholines (mmol/l) | 2.0 (0.3) | 2.0 (0.3) | 2.0 (0.3) | 0.36 |
|  | | | Sphingomyelins (mmol/l) | 0.501 (0.087) | 0.500 (0.086) | 0.506 (0.091) | 0.32 |
|  | | | Total cholines (mmol/l) | 2.4 (0.4) | 2.4 (0.4) | 2.4 (0.4) | 0.36 |

| **METABOLITE** | | | | | | **Total population** | **Dementia** | |  |
| --- | --- | --- | --- | --- | --- | --- | --- | --- | --- |
|  |  |  |  |  |  |  | **No** | **Yes** | **p-value^*^** |
| **Fatty acids related metabolites** | | | | | |  |  |  |  |
|  | Total fatty acids (mmol/l) | | | | | 12.4 (2.2) | 12.4 (2.1) | 12.5 (2.3) | 0.19 |
|  | Estimated description of fatty acid chain length, not actual carbon number | | | | | 17.4 (0.3) | 17.4 (0.3) | 17.4 (0.3) | 0.65 |
|  | Unsaturation degree | | | | | 1.2 (0.1) | 1.2 (0.1) | 1.2 (0.1) | 0.69 |
|  | 22:6, docosahexaenoic acid (mmol/l) | | | | | 0.194 (0.064) | 0.193 (0.064) | 0.203 (0.667) | **0.01** |
|  | 18:2, linoleic acid (mmol/l) | | | | | 3.4 (0.6) | 3.4 (0.6) | 3.4 (0.7) | 0.66 |
|  | Conjugated linoleic acid (mmol/l) | | | | | 0.052 (0.025) | 0.052 (0.025) | 0.054 (0.029) | 0.36 |
|  | Omega-3 fatty acids (mmol/l) | | | | | 0.531 (0.152) | 0.530 (0.151) | 0.552 (0.162) | **0.03** |
|  | Omega-6 fatty acids (mmol/l) | | | | | 4.2 (0.7) | 4.2 (0.7) | 4.2 (0.7) | 0.43 |
|  | Polyunsaturated fatty acids (mmol/l) | | | | | 4.7 (0.7) | 4.7 (0.7) | 4.8 (0.8) | 0.19 |
|  | Monounsaturated fatty acids; 16:1, 18:1 (mmol/l) | | | | | 3.0 (0.8) | 3.0 (0.8) | 3.1 (0.9) | 0.50 |
|  | Saturated fatty acids (mmol/l) | | | | | 4.6 (0.9) | 4.6 (0.8) | 4.7 (0.9) | 0.28 |
|  | Ratio of 22:6 docosahexaenoic acid to total fatty acids (%) | | | | | 1.6 (0.4) | 1.6 (0.4) | 1.6 (0.5) | **0.03** |
|  | Ratio of 18:2 linoleic acid to total fatty acids (%) | | | | | 27.8 (3.5) | 27.8 (3.5) | 27.6 (3.8) | 0.62 |
|  | Ratio of conjugated linoleic acid to total fatty acids (%) | | | | | 0.411 (0.167) | 0.411 (0.165) | 0.419 (0.193) | 0.80 |
|  | Ratio of omega-3 fatty acids to total fatty acids (%) | | | | | 4.3 (1.0) | 4.3 (1.0) | 4.4 (1.2) | 0.06 |
|  | Ratio of omega-6 fatty acids to total fatty acids (%) | | | | | 34.0 (3.3) | 34.0 (3.3) | 33.8 (3.6) | 0.62 |
|  | Ratio of polyunsaturated fatty acids to total fatty acids (%) | | | | | 38.3 (3.4) | 38.3 (3.4) | 38.2 (3.7) | 0.96 |
|  | Ratio of monounsaturated fatty acids to total fatty acids (%) | | | | | 24.4 (3.1) | 24.4 (3.0) | 24.4 (3.3) | 0.98 |
|  | Ratio of saturated fatty acids to total fatty acids (%) | | | | | 37.3 (1.7) | 37.3 (1.7) | 37.3 (1.7) | 0.85 |
| **Apoliporproteins related metabolites** | | | | | |  |  |  |  |
|  | Apolipoprotein a-i (g/l) | | | | | 1.6 (0.2) | 1.6 (0.2) | 1.6 (0.2) | 0.51 |
|  | Apolipoprotein b (g/l) | | | | | 1.0 (0.2) | 1.0 (0.2) | 1.0 (0.2) | 0.05 |
|  | Ratio of apolipoprotein b to apolipoprotein a-i | | | | | 0.629 (0.124) | 0.628 (0.125) | 0.637 (0.114) | 0.13 |
| **Glycolisis related metabolites** | | | | | |  |  |  |  |
|  | Glucose (mmol/l) | | | | | 5.2 (1.2) | 5.2 (1.1) | 5.4 (1.6) | **0.002** |
|  | | Lactate (mmol/l) | | | | 1.9 (0.6) | 1.9 (0.6) | 1.9 (0.5) | 0.22 |
|  | | Pyruvate (mmol/l) | | | | 0.091 (0.031) | 0.091 (0.031) | 0.090 (0.031) | 0.56 |
|  | | Citrate (mmol/l) | | | | 0.129 (0.022) | 0.128 (0.022) | 0.133 (0.023) | **0.002** |
|  | | Glycerol (mmol/l) | | | | 0.100 (0.035) | 0.099 (0.035) | 0.106 (0.038) | **0.001** |
| **Amino acids** | | | | | |  |  |  |  |
|  | | | Alanine (mmol/l) | | | 0.424 (0.061) | 0.424 (0.061) | 0.421 (0.064) | 0.30 |
|  | | | Glutamine (mmol/l) | | | 0.609 (0.064) | 0.609 (0.063) | 0.614 (0.071) | 0.22 |
| **METABOLITE** | | | | | | **Total population** | **Dementia** | |  |
|  |  |  |  |  |  |  | **No** | **Yes** | **p-value^*^** |
| **Amino acids** | | | | | |  |  |  |  |
|  | | | Glycine (mmol/l) | | | 0.288 (0.064) | 0.288 (0.064) | 0.286 (0.065) | 0.44 |
|  | | | Histidine (mmol/l) | | | 0.075 (0.009) | 0.075 (0.009) | 0.074 (0.009) | **0.03** |
|  | | | Isoleucine (mmol/l) | | | 0.060 (0.015) | 0.060 (0.015) | 0.060 (0.017) | 0.37 |
|  | | | Leucine (mmol/l) | | | 0.088 (0.017) | 0.088 (0.017) | 0.087 (0.018) | **0.04** |
|  | | | Valine (mmol/l) | | | 0.197 (0.034) | 0.197 (0.034) | 0.197 (0.037) | 0.72 |
|  | | | Phenylalanine (mmol/l) | | | 0.083 (0.010) | 0.083 (0.010) | 0.084 (0.011) | 0.61 |
|  | | | Tyrosine (mmol/l) | | | 0.057 (0.010) | 0.057 (0.010) | 0.057 (0.011) | 0.82 |
| **Ketone bodies** | | | | | |  |  |  |  |
|  | | | Acetate (mmol/l) | | | 0.069 (0.023) | 0.069 (0.023) | 0.069 (0.022) | 0.79 |
|  | | | Acetoacetate (mmol/l) | | | 0.059 (0.043) | 0.058 (0.043) | 0.067 (0.045) | **< 0.001** |
|  | | | β-hydroxybutyrate (mmol/l) | | | 0.151 (0.106) | 0.149 (0.105) | 0.173 (0.118) | **< 0.001** |
| **Fluid balance** | | | | | |  |  |  |  |
|  | | | | | Creatinine (mmol/l) | 0.078 (0.012) | 0.078 (0.011) | 0.078 (0.015) | 0.31 |
|  | | | | | Albumin (signal area) | 0.101 (0.006) | 0.101 (0.006) | 0.100 (0.006) | **0.01** |
| **Inflammation** | | | | | |  |  |  |  |
|  | | | | Glycoprotein acetyls, mainly a1-acid glycoprotein (mmol/l) | | 1.5 (0.2) | 1.5 (0.2) | 1.5 (0.2) | 0.80 |

VLDL: very low density lipoproteins, IDL: intermediate-density lipoproteins, LDL: low density lipoproteins, HDL: high density lipoprotein.

Data are M (SD).

^*^p values for differences in student’s *t* test.

**Table S2. The association between 1-SD increment in metabolite concentrations (separate models) and risk of dementia.**

| **METABOLITE** | | | | | | | **HR (95% CI)** | **p-value** |
| --- | --- | --- | --- | --- | --- | --- | --- | --- |
| **Chylomicrons and extremely large VLDL** | | | | | | |  |  |
|  | | Concentration of chylomicrons and extremely large VLDL particles (mol/l) | | | | | 0.93 (0.77, 1.13) | 0.46 |
|  | | Total lipids in chylomicrons and extremely large VLDL (mmol/l) | | | | | 0.92 (0.76, 1.13) | 0.44 |
|  | | Phospholipids in chylomicrons and extremely large VLDL (mmol/l) | | | | | 0.92 (0.78, 1.09) | 0.36 |
|  | | Total cholesterol in chylomicrons and extremely large VLDL (mmol/l) | | | | | 0.90 (0.76, 1.08) | 0.26 |
|  | | Cholesterol esters in chylomicrons and extremely large VLDL (mmol/l) | | | | | 0.90 (0.75, 1.08) | 0.25 |
|  | | Free cholesterol in chylomicrons and extremely large VLDL (mmol/l) | | | | | 0.91 (0.78, 1.07) | 0.25 |
|  | | Triglycerides in chylomicrons and extremely large VLDL (mmol/l) | | | | | 0.94 (0.77, 1.14) | 0.52 |
| **Very large VLDL** | | | | | | |  |  |
|  | | | | Concentration of very large VLDL particles (mol/l) | | | 0.89 (0.71, 1.11) | 0.29 |
|  | | | | Total lipids in very large VLDL (mmol/l) | | | 0.86 (0.65, 1.14) | 0.30 |
|  | | | | Phospholipids in very large VLDL (mmol/l) | | | 0.86 (0.67, 1.11) | 0.25 |
|  | | | | Total cholesterol in very large VLDL (mmol/l) | | | 0.85 (0.63, 1.14) | 0.27 |
|  | | | | Cholesterol esters in very large VLDL (mmol/l) | | | 0.88 (0.69, 1.12) | 0.30 |
|  | | | | Free cholesterol in very large VLDL (mmol/l) | | | 0.87 (0.66, 1.15) | 0.32 |
|  | | | | Triglycerides in very large VLDL (mmol/l) | | | 0.88 (0.70, 1.11) | 0.28 |
| **Large VLDL** | | | | | | |  |  |
|  | | | Concentration of large VLDL particles (mol/l) | | | | 0.94 (0.78, 1.13) | 0.50 |
|  | | | Total lipids in large VLDL (mmol/l) | | | | 0.90 (0.66, 1.21) | 0.48 |
|  | | | Phospholipids in large VLDL (mmol/l) | | | | 0.91 (0.69, 1.20) | 0.50 |
|  | | | Total cholesterol in large VLDL (mmol/l) | | | | 0.85 (0.63, 1.15) | 0.30 |
|  | | | Cholesterol esters in large VLDL (mmol/l) | | | | 0.82 (0.59, 1.15) | 0.25 |
|  | | | Free cholesterol in large VLDL (mmol/l) | | | | 0.90 (0.72, 1.11) | 0.33 |
|  | | | Triglycerides in large VLDL (mmol/l) | | | | 0.92 (0.70, 1.21) | 0.56 |
| **Medium VLDL** | | | | | | |  |  |
|  | Concentration of medium VLDL particles (mol/l) | | | | | | 0.96 (0.85, 1.09) | 0.57 |
|  | Total lipids in medium VLDL (mmol/l) | | | | | | 0.95 (0.82, 1.11) | 0.55 |
| **METABOLITE** | | | | | | | **HR (95% CI)** | **p-value** |
| **Medium VLDL** | | | | | | |  |  |
|  | Phospholipids in medium VLDL (mmol/l) | | | | | | 0.96 (0.83, 1.11) | 0.58 |
|  | Total cholesterol in medium VLDL (mmol/l) | | | | | | 0.93 (0.80, 1.09) | 0.39 |
|  | Cholesterol esters in medium VLDL (mmol/l) | | | | | | 0.92 (0.78, 1.08) | 0.31 |
|  | Free cholesterol in medium VLDL (mmol/l) | | | | | | 0.95 (0.83, 1.10) | 0.49 |
|  | Triglycerides in medium VLDL (mmol/l) | | | | | | 0.97 (0.84, 1.12) | 0.65 |
| **Small VLDL** | | | | | | |  |  |
|  | | | | Concentration of small VLDL particles (mol/l) | | | 0.97 (0.86, 1.10) | 0.65 |
|  | | | | Total lipids in small VLDL (mmol/l) | | | 0.96 (0.81, 1.14) | 0.65 |
|  | | | | Phospholipids in small VLDL (mmol/l) | | | 0.96 (0.81, 1.13) | 0.62 |
|  | | | | Total cholesterol in small VLDL (mmol/l) | | | 0.96 (0.80, 1.15) | 0.66 |
|  | | | | Cholesterol esters in small VLDL (mmol/l) | | | 0.96 (0.80, 1.15) | 0.67 |
|  | | | | Free cholesterol in small VLDL (mmol/l) | | | 0.96 (0.81, 1.13) | 0.61 |
|  | | | | Triglycerides in small VLDL (mmol/l) | | | 0.96 (0.83, 1.12) | 0.62 |
| **Very small VLDL** | | | | | | |  |  |
|  | | | | | | Concentration of very small VLDL particles (mol/l) | 1.00 (0.88, 1.14) | 0.94 |
|  | | | | | | Total lipids in very small VLDL (mmol/l) | 1.01 (0.81, 1.26) | 0.95 |
|  | | | | | | Phospholipids in very small VLDL (mmol/l) | 1.00 (0.82, 1.23) | 0.99 |
|  | | | | | | Total cholesterol in very small VLDL (mmol/l) | 1.01 (0.81, 1.26) | 0.92 |
|  | | | | | | Cholesterol esters in very small VLDL (mmol/l) | 1.03 (0.83, 1.28) | 0.90 |
|  | | | | | | Free cholesterol in very small VLDL (mmol/l) | 0.98 (0.81, 1.19) | 0.84 |
|  | | | | | | Triglycerides in very small VLDL (mmol/l) | 0.98 (0.83, 1.15) | 0.79 |
| **IDL** | | | | | | |  |  |
|  | | | | Concentration of IDL particles (mol/l) | | | 1.00 (0.89, 1.13) | 0.99 |
|  | | | | Total lipids in IDL (mmol/l) | | | 1.00 (0.88, 1.12) | 0.97 |
|  | | | | Phospholipids in IDL (mmol/l) | | | 0.98 (0.76, 1.25) | 0.85 |
|  | | | | Total cholesterol in IDL (mmol/l) | | | 0.98 (0.78, 1.24) | 0.89 |
|  | | | | Cholesterol esters in IDL (mmol/l) | | | 0.99 (0.79, 1.23) | 0.91 |
|  | | | | Free cholesterol in IDL (mmol/l) | | | 0.98 (0.79, 1.21) | 0.83 |
|  | | | | Triglycerides in IDL (mmol/l) | | | 1.04 (0.85, 1.26) | 0.71 |
| **METABOLITE** | | | | | | | **HR (95% CI)** | **p-value** |
| **Large LDL** | | | | | | |  |  |
|  | | | Concentration of large LDL particles (mol/l) | | | | 1.00 (0.88, 1.13) | 0.98 |
|  | | | Total lipids in large LDL (mmol/l) | | | | 0.99 (0.78, 1.27) | 0.96 |
|  | | | Phospholipids in large LDL (mmol/l) | | | | 0.98 (0.76, 1.27) | 0.90 |
|  | | | Total cholesterol in large LDL (mmol/l) | | | | 0.98 (0.79, 1.23) | 0.88 |
|  | | | Cholesterol esters in large LDL (mmol/l) | | | | 0.98 (0.80, 1.21) | 0.86 |
|  | | | Free cholesterol in large LDL (mmol/l) | | | | 0.99 (0.79, 1.24) | 0.93 |
|  | | | Triglycerides in large LDL (mmol/l) | | | | 1.06 (0.87, 1.30) | 0.56 |
| **Medium LDL** | | | | | | |  |  |
|  | Concentration of medium LDL particles (mol/l) | | | | | | 1.00 (0.88, 1.13) | 0.99 |
|  | Total lipids in medium LDL (mmol/l) | | | | | | 1.00 (0.79, 1.27) | 0.99 |
|  | Phospholipids in medium LDL (mmol/l) | | | | | | 1.02 (0.78, 1.35) | 0.87 |
|  | | | | Total cholesterol in medium LDL (mmol/l) | | | 0.99 (0.80, 1.22) | 0.90 |
|  | | | | Cholesterol esters in medium LDL (mmol/l) | | | 0.98 (0.81, 1.18) | 0.80 |
|  | | | | Free cholesterol in medium LDL (mmol/l) | | | 1.04 (0.80, 1.35) | 0.79 |
|  | | | | Triglycerides in medium LDL (mmol/l) | | | 1.05 (0.86, 1.29) | 0.61 |
| **Small LDL** | | | | | | |  |  |
|  | | | | | Concentration of small LDL particles (mol/l) | | 1.00 (0.89, 1.13) | 0.95 |
|  | | | | | Total lipids in small LDL (mmol/l) | | 1.01 (0.80, 1.27) | 0.94 |
|  | | | | | Phospholipids in small LDL (mmol/l) | | 1.03 (0.78, 1.37) | 0.82 |
|  | | | | | Total cholesterol in small LDL (mmol/l) | | 1.00 (0.82, 1.21) | 0.98 |
|  | | | | | Cholesterol esters in small LDL (mmol/l) | | 0.99 (0.83, 1.18) | 0.89 |
|  | | | | | Free cholesterol in small LDL (mmol/l) | | 1.05 (0.82, 1.33) | 0.72 |
|  | | | | | Triglycerides in small LDL (mmol/l) | | 0.99 (0.84, 1.18) | 0.95 |
| **Very large HDL** | | | | | | |  |  |
|  | | Concentration of very large HDL particles (mol/l) | | | | | 1.04 (0.92, 1.18) | 0.51 |
|  | | Total lipids in very large HDL (mmol/l) | | | | | 1.08 (0.86, 1.36) | 0.50 |
|  | | Phospholipids in very large HDL (mmol/l) | | | | | 1.07 (0.87, 1.32) | 0.52 |
|  | | Total cholesterol in very large HDL (mmol/l) | | | | | 1.08 (0.87, 1.34) | 0.48 |
|  | | Cholesterol esters in very large HDL (mmol/l) | | | | | 1.08 (0.88, 1.33) | 0.48 |
| **METABOLITE** | | | | | | | **HR (95% CI)** | **p-value** |
| **Very large HDL** | | | | | | |  |  |
|  | | Free cholesterol in very large HDL (mmol/l) | | | | | 1.06 (0.87, 1.30) | 0.55 |
|  | | Triglycerides in very large HDL (mmol/l) | | | | | 0.94 (0.80, 1.11) | 0.50 |
| **Large HDL** | | | | | | |  |  |
|  | | Concentration of large HDL particles (mol/l) | | | | | 1.00 (0.88, 1.13) | 0.97 |
|  | | Total lipids in large HDL (mmol/l) | | | | | 1.00 (0.83, 1.20) | 0.99 |
|  | | Phospholipids in large HDL (mmol/l) | | | | | 0.99 (0.82, 1.20) | 0.92 |
|  | | Total cholesterol in large HDL (mmol/l) | | | | | 1.01 (0.85, 1.19) | 0.92 |
|  | | Cholesterol esters in large HDL (mmol/l) | | | | | 1.01 (0.85, 1.19) | 0.93 |
|  | | Free cholesterol in large HDL (mmol/l) | | | | | 1.01 (0.87, 1.18) | 0.89 |
|  | | Triglycerides in large HDL (mmol/l) | | | | | 0.96 (0.82, 1.11) | 0.58 |
| **Medium HDL** | | | | | | |  |  |
|  | | | Concentration of medium HDL particles (mol/l) | | | | 0.94 (0.83, 1.05) | 0.27 |
|  | | | Total lipids in medium HDL (mmol/l) | | | | 0.85 (0.64, 1.14) | 0.28 |
|  | | | Phospholipids in medium HDL (mmol/l) | | | | 0.90 (0.68, 1.19) | 0.46 |
|  | | | Total cholesterol in medium HDL (mmol/l) | | | | 0.86 (0.67, 1.11) | 0.25 |
|  | | | Cholesterol esters in medium HDL (mmol/l) | | | | 0.85 (0.66, 1.10) | 0.22 |
|  | | | Free cholesterol in medium HDL (mmol/l) | | | | 0.91 (0.74, 1.12) | 0.37 |
|  | | | Triglycerides in medium HDL (mmol/l) | | | | 0.84 (0.69, 1.03) | 0.09 |
| **Small HDL** | | | | | | |  |  |
|  | | | Concentration of small HDL particles (mol/l) | | | | 0.97 (0.86, 1.09) | 0.63 |
|  | | | Total lipids in small HDL (mmol/l) | | | | 0.91 (0.63, 1.32) | 0.63 |
|  | | | Phospholipids in small HDL (mmol/l) | | | | 0.87 (0.63, 1.20) | 0.39 |
|  | | | Total cholesterol in small HDL (mmol/l) | | | | 1.01 (0.75, 1.35) | 0.96 |
|  | | | Cholesterol esters in small HDL (mmol/l) | | | | 1.03 (0.81, 1.33) | 0.79 |
|  | | | Free cholesterol in small HDL (mmol/l) | | | | 0.91 (0.71, 1.17) | 0.46 |
|  | | | Triglycerides in small HDL (mmol/l) | | | | 1.01 (0.85, 1.18) | 0.95 |
| **Chylomicrons and extremely large VLDL ratios** | | | | | | |  |  |
|  | | Phospholipids to total lipids ratio in chylomicrons and extremely large VLDL (%) | | | | | 0.88 (0.74, 1.04) | 0.14 |
|  | | Total cholesterol to total lipids ratio in chylomicrons and extremely large VLDL (%) | | | | | 0.84 (0.73, 0.96) | **0.01** |
| **METABOLITE** | | | | | | | **HR (95% CI)** | **p-value** |
| **Chylomicrons and extremely large VLDL ratios** | | | | | | |  |  |
|  | | Cholesterol esters to total lipids ratio in chylomicrons and extremely large VLDL (%) | | | | | 0.90 (0.79, 1.03) | 0.14 |
|  | | Free cholesterol to total lipids ratio in chylomicrons and extremely large VLDL (%) | | | | | 0.86 (0.74, 0.99) | **0.04** |
|  | | Triglycerides to total lipids ratio in chylomicrons and extremely large VLDL (%) | | | | | 1.17 (1.04, 1.33) | **0.01** |
| **Very large VLDL ratios** | | | | | | |  |  |
|  | | | Phospholipids to total lipids ratio in very large VLDL (%) | | | | 0.98 (0.85, 1.12) | 0.74 |
|  | | | Total cholesterol to total lipids ratio in very large VLDL (%) | | | | 1.03 (0.91, 1.15) | 0.67 |
|  | | | Cholesterol esters to total lipids ratio in very large VLDL (%) | | | | 1.01 (0.89, 1.14) | 0.90 |
|  | | | Free cholesterol to total lipids ratio in very large VLDL (%) | | | | 1.04 (0.93, 1.17) | 0.46 |
|  | | | Triglycerides to total lipids ratio in very large VLDL (%) | | | | 0.95 (0.84, 1.08) | 0.41 |
| **Large VLDL ratios** | | | | | | |  |  |
|  | | | | Phospholipids to total lipids ratio in large VLDL (%) | | | 1.05 (0.92, 1.20) | 0.48 |
|  | | | | Total cholesterol to total lipids ratio in large VLDL (%) | | | 0.92 (0.80, 1.05) | 0.21 |
|  | | | | Cholesterol esters to total lipids ratio in large VLDL (% | | | 0.97 (0.85, 1.10) | 0.63 |
|  | | | | Free cholesterol to total lipids ratio in large VLDL (%) | | | 0.90 (0.79, 1.03) | 0.12 |
|  | | | | Triglycerides to total lipids ratio in large VLDL (%) | | | 1.06 (0.90, 1.25) | 0.46 |
| **Medium VLDL ratios** | | | | | | |  |  |
|  | | | | | Phospholipids to total lipids ratio in medium VLDL (%) | | 1.06 (0.94, 1.20) | 0.33 |
|  | | | | | Total cholesterol to total lipids ratio in medium VLDL (%) | | 0.97 (0.85, 1.09) | 0.58 |
|  | | | | | Cholesterol esters to total lipids ratio in medium VLDL (%) | | 0.98 (0.86, 1.11) | 0.71 |
|  | | | | | Free cholesterol to total lipids ratio in medium VLDL (%) | | 0.92 (0.80, 1.06) | 0.26 |
|  | | | | | Triglycerides to total lipids ratio in medium VLDL (%) | | 1.03 (0.88, 1.20) | 0.75 |
| **Small VLDL ratios** | | | | | | |  |  |
|  | | | Phospholipids to total lipids ratio in small VLDL (%) | | | | 0.99 (0.88, 1.13) | 0.93 |
|  | | | Total cholesterol to total lipids ratio in small VLDL (%) | | | | 1.01 (0.90, 1.14) | 0.84 |
|  | | | Cholesterol esters to total lipids ratio in small VLDL (%) | | | | 1.01 (0.90, 1.14) | 0.85 |
|  | | | | | Free cholesterol to total lipids ratio in small VLDL (%) | | 0.97 (0.86, 1.10) | 0.61 |
|  | | | | | Triglycerides to total lipids ratio in small VLDL (%) | | 0.97 (0.86, 1.10) | 0.65 |
| **Very small VLDL ratios** | | | | | | |  |  |
|  | | | Phospholipids to total lipids ratio in very small VLDL (%) | | | | 0.99 (0.87, 1.12) | 0.86 |
| **METABOLITE** | | | | | | | **HR (95% CI)** | **p-value** |
| **Very small VLDL ratios** | | | | | | |  |  |
|  | | | Total cholesterol to total lipids ratio in very small VLDL (%) | | | | 1.01 (0.89, 1.14) | 0.91 |
|  | | | Cholesterol esters to total lipids ratio in very small VLDL (%) | | | | 1.03 (0.91, 1.17) | 0.65 |
|  | | | Free cholesterol to total lipids ratio in very small VLDL (%) | | | | 0.93 (0.83, 1.05) | 0.27 |
|  | | | Triglycerides to total lipids ratio in very small VLDL (%) | | | | 0.97 (0.86, 1.09) | 0.62 |
| **IDL ratios** | | | | | | |  |  |
|  | | | | | Phospholipids to total lipids ratio in IDL (%) | | 0.94 (0.83, 1.06) | 0.29 |
|  | | | | | Total cholesterol to total lipids ratio in IDL (%) | | 0.96 (0.85, 1.08) | 0.51 |
|  | | | | | Cholesterol esters to total lipids ratio in IDL (%) | | 0.98 (0.86, 1.10) | 0.69 |
|  | | | | | Free cholesterol to total lipids ratio in IDL (%) | | 0.95 (0.84, 1.07) | 0.35 |
|  | | | | | Triglycerides to total lipids ratio in IDL (%) | | 1.03 (0.92, 1.15) | 0.65 |
| **Large LDL ratios** | | | | | | |  |  |
|  | | | | Phospholipids to total lipids ratio in large LDL (%) | | | 0.98 (0.86, 1.11) | 0.75 |
|  | | | | Total cholesterol to total lipids ratio in large LDL (%) | | | 0.95 (0.83, 1.09) | 0.46 |
|  | | | | Cholesterol esters to total lipids ratio in large LDL (%) | | | 0.94 (0.80, 1.11) | 0.46 |
|  | | | | Free cholesterol to total lipids ratio in large LDL (%) | | | 0.99 (0.87, 1.11) | 0.83 |
|  | | | | Triglycerides to total lipids ratio in large LDL (%) | | | 1.04 (0.93, 1.17) | 0.48 |
| **Medium LDL ratios** | | | | | | |  |  |
|  | | | | | | Phospholipids to total lipids ratio in medium LDL (%) | 1.02 (0.91, 1.15) | 0.70 |
|  | | | | | | Total cholesterol to total lipids ratio in medium LDL (%) | 0.96 (0.85, 1.09) | 0.52 |
|  | | | | | | Cholesterol esters to total lipids ratio in medium LDL (%) | 0.94 (0.80, 1.09) | 0.42 |
|  | | | | | | Free cholesterol to total lipids ratio in medium LDL (%) | 1.05 (0.93, 1.19) | 0.43 |
|  | | | | | | Triglycerides to total lipids ratio in medium LDL (%) | 1.03 (0.92, 1.16) | 0.59 |
| **Small LDL ratios** | | | | | | |  |  |
|  | | | | | Phospholipids to total lipids ratio in small LDL (%) | | 1.01 (0.90, 1.14) | 0.84 |
|  | | | | | Total cholesterol to total lipids ratio in small LDL (%) | | 0.98 (0.86, 1.11) | 0.72 |
|  | | | | | Cholesterol esters to total lipids ratio in small LDL (%) | | 0.96 (0.83, 1.11) | 0.61 |
|  | | | | | Free cholesterol to total lipids ratio in small LDL (%) | | 1.05 (0.93, 1.19) | 0.44 |
|  | | | | | Triglycerides to total lipids ratio in small LDL (%) | | 0.99 (0.88, 1.11) | 0.89 |
| **METABOLITE** | | | | | | | **HR (95% CI)** | **p-value** |
| **Very large HDL ratios** | | | | | | |  |  |
|  | | | | | Phospholipids to total lipids ratio in very large HDL (%) | | 1.01 (0.89, 1.14) | 0.91 |
|  | | | | | Total cholesterol to total lipids ratio in very large HDL (%) | | 1.01 (0.89, 1.15) | 0.86 |
|  | | | | | Cholesterol esters to total lipids ratio in very large HDL (%) | | 1.02 (0.89, 1.16) | 0.83 |
|  | | | | | Free cholesterol to total lipids ratio in very large HDL (%) | | 0.99 (0.87, 1.11) | 0.83 |
|  | | | | | Triglycerides to total lipids ratio in very large HDL (%) | | 0.93 (0.82, 1.05) | 0.21 |
| **Large HDL ratios** | | | | | | |  |  |
|  | | | | | Phospholipids to total lipids ratio in large HDL (%) | | 0.97 (0.86, 1.09) | 0.59 |
|  | | | | | Total cholesterol to total lipids ratio in large HDL (%) | | 1.05 (0.93, 1.18) | 0.44 |
|  | | | | | Cholesterol esters to total lipids ratio in large HDL (%) | | 1.05 (0.93, 1.18) | 0.43 |
|  | | | | | Free cholesterol to total lipids ratio in large HDL (%) | | 1.04 (0.92, 1.17) | 0.55 |
|  | | | | | Triglycerides to total lipids ratio in large HDL (%) | | 0.96 (0.85, 1.08) | 0.46 |
| **Medium HDL ratios** | | | | | | |  |  |
|  | | | | | | Phospholipids to total lipids ratio in medium HDL (%) | 1.15 (1.03, 1.29) | **0.01** |
|  | | | | | | Total cholesterol to total lipids ratio in medium HDL (%) | 0.95 (0.84, 1.06) | 0.36 |
|  | | | | | | Cholesterol esters to total lipids ratio in medium HDL (%) | 0.95 (0.84, 1.06) | 0.34 |
|  | | | | | | Free cholesterol to total lipids ratio in medium HDL (%) | 0.99 (0.88, 1.11) | 0.86 |
|  | | | | | | Triglycerides to total lipids ratio in medium HDL (%) | 0.95 (0.84, 1.07) | 0.40 |
| **Small HDL ratios** | | | | | | |  |  |
|  | | | | | Phospholipids to total lipids ratio in small HDL (%) | | 0.94 (0.84, 1.05) | 0.29 |
|  | | | | | Total cholesterol to total lipids ratio in small HDL (%) | | 1.04 (0.93, 1.17) | 0.48 |
|  | | | | | Cholesterol esters to total lipids ratio in small HDL (%) | | 1.04 (0.93, 1.17) | 0.47 |
|  | | | | | Free cholesterol to total lipids ratio in small HDL (%) | | 0.94 (0.85, 1.05) | 0.29 |
|  | | | | | Triglycerides to total lipids ratio in small HDL (%) | | 1.02 (0.90, 1.15) | 0.78 |
| **Lipoprotein particle sizes** | | | | | | |  |  |
|  | | | | Mean diameter for VLDL particles (nm) | | | 0.96 (0.85, 1.09) | 0.53 |
|  | | | | Mean diameter for LDL particles (nm) | | | 0.99 (0.87, 1.11) | 0.83 |
|  | | | | Mean diameter for HDL particles (nm) | | | 1.01 (0.89, 1.14) | 0.88 |

| **METABOLITE** | | | | | | | | **HR (95% CI)** | **p-value** |
| --- | --- | --- | --- | --- | --- | --- | --- | --- | --- |
| **Cholesterol related metabolites** | | | | | | | |  |  |
|  | | Serum total cholesterol (mmol/l) | | | | | | 0.98 (0.87, 1.10) | 0.69 |
|  | | Total cholesterol in VLDL (mmol/l) | | | | | | 0.98 (0.86, 1.10) | 0.69 |
|  | | Remnant cholesterol (non-HDL, non-LDL -cholesterol) (mmol/l) | | | | | | 0.98 (0.87, 1.11) | 0.80 |
|  | | Total cholesterol in LDL (mmol/l) | | | | | | 0.99 (0.88, 1.12) | 0.90 |
|  | | Total cholesterol in HDL (mmol/l) | | | | | | 0.99 (0.87, 1.11) | 0.81 |
|  | | Total cholesterol in HDL2 (mmol/l) | | | | | | 0.98 (0.87, 1.10) | 0.74 |
|  | | Total cholesterol in HDL3 (mmol/l) | | | | | | 1.06 (0.94, 1.19) | 0.33 |
|  | | Esterified cholesterol (mmol/l) | | | | | | 0.98 (0.87, 1.11) | 0.78 |
|  | | Free cholesterol (mmol/l) | | | | | | 0.96 (0.85, 1.08) | 0.49 |
| **Lipids related metabolites** | | | | | | | |  |  |
|  | Serum total triglycerides (mmol/l) | | | | | | | 0.97 (0.86, 1.10) | 0.65 |
|  | | | Triglycerides in VLDL (mmol/l) | | | | | 0.97 (0.86, 1.10) | 0.63 |
|  | | | Triglycerides in LDL (mmol/l) | | | | | 1.03 (0.91, 1.16) | 0.65 |
|  | | | Triglycerides in HDL (mmol/l) | | | | | 0.93 (0.83, 1.05) | 0.26 |
|  | | | Diacylglycerol (mmol/l) | | | | | 0.98 (0.87, 1.11) | 0.80 |
|  | | | Ratio of diacylglycerol to triglycerides | | | | | 0.99 (0.87, 1.12) | 0.85 |
|  | | | Total phosphoglycerides (mmol/l) | | | | | 0.95 (0.84, 1.07) | 0.41 |
|  | | | Ratio of triglycerides to phosphoglycerides | | | | | 0.97 (0.86, 1.09) | 0.62 |
|  | | | Phosphatidylcholine and other cholines (mmol/l) | | | | | 0.95 (0.84, 1.07) | 0.40 |
|  | | | Sphingomyelins (mmol/l) | | | | | 0.92 (0.82, 1.03) | 0.13 |
|  | | | Total cholines (mmol/l) | | | | | 0.94 (0.84, 1.07) | 0.35 |
| **Fatty acids related metabolites** | | | | | | | |  |  |
|  | | Total fatty acids (mmol/l) | | | | | | 0.97 (0.86, 1.10) | 0.65 |
|  | | Estimated description of fatty acid chain length, not actual carbon number | | | | | | 0.98 (0.87, 1.10) | 0.71 |
|  | | Unsaturation degree | | | | | | 0.94 (0.84, 1.06) | 0.30 |
|  | | 22:6, docosahexaenoic acid (mmol/l) | | | | | | 0.96 (0.86, 1.08) | 0.51 |
|  | | 18:2, linoleic acid (mmol/l) | | | | | | 0.92 (0.82, 1.04) | 0.18 |
|  | | Conjugated linoleic acid (mmol/l) | | | | | | 0.92 (0.76, 1.10) | 0.35 |
|  | | Omega-3 fatty acids (mmol/l) | | | | | | 0.98 (0.87, 1.10) | 0.71 |
| **METABOLITE** | | | | | | | | **HR (95% CI)** | **p-value** |
| **Fatty acids related metabolites** | | | | | | | |  |  |
|  | | Omega-6 fatty acids (mmol/l) | | | | | | 0.92 (0.82, 1.03) | 0.15 |
|  | | Polyunsaturated fatty acids (mmol/l) | | | | | | 0.92 (0.82, 1.04) | 0.19 |
|  | | Monounsaturated fatty acids; 16:1, 18:1 (mmol/l) | | | | | | 1.00 (0.88, 1.12) | 0.95 |
|  | | Saturated fatty acids (mmol/l) | | | | | | 0.99 (0.88, 1.12) | 0.91 |
|  | | Ratio of 22:6 docosahexaenoic acid to total fatty acids (%) | | | | | | 0.97 (0.87, 1.09) | 0.63 |
|  | | Ratio of 18:2 linoleic acid to total fatty acids (%) | | | | | | 0.93 (0.83, 1.04) | 0.21 |
|  | | Ratio of conjugated linoleic acid to total fatty acids (%) | | | | | | 0.90 (0.72, 1.13) | 0.37 |
|  | | Ratio of omega-3 fatty acids to total fatty acids (%) | | | | | | 0.99 (0.89, 1.11) | 0.91 |
|  | | Ratio of omega-6 fatty acids to total fatty acids (%) | | | | | | 0.91 (0.82, 1.02) | 0.11 |
|  | | Ratio of polyunsaturated fatty acids to total fatty acids (%) | | | | | | 0.92 (0.82, 1.03) | 0.14 |
|  | | Ratio of monounsaturated fatty acids to total fatty acids (%) | | | | | | 1.03 (0.92, 1.16) | 0.62 |
|  | | Ratio of saturated fatty acids to total fatty acids (%) | | | | | | 1.07 (0.96, 1.20) | 0.24 |
| **Apoliporproteins related metabolites** | | | | | | | |  |  |
|  | | Apolipoprotein a-i (g/l) | | | | | | 0.96 (0.85, 1.08) | 0.50 |
|  | | Apolipoprotein b (g/l) | | | | | | 0.99 (0.88, 1.12) | 0.89 |
|  | | Ratio of apolipoprotein b to apolipoprotein a-i | | | | | | 1.02 (0.90, 1.15) | 0.79 |
| **Glycolisis related metabolites** | | | | | | | |  |  |
|  | | Glucose (mmol/l) | | | | | | 1.24 (1.13, 1.36) | **0.00001*** |
|  | | | | Lactate (mmol/l) | | | | 0.98 (0.88, 1.09) | 0.65 |
|  | | | | Pyruvate (mmol/l) | | | | 1.04 (0.94, 1.16) | 0.42 |
|  | | | | Citrate (mmol/l) | | | | 1.08 (0.96, 1.20) | 0.20 |
|  | | | | Glycerol (mmol/l) | | | | 1.03 (0.91, 1.16) | 0.68 |
| **Amino acids** | | | | | | | |  |  |
|  | | | | | Alanine (mmol/l) | | | 0.92 (0.83, 1.03) | 0.17 |
|  | | | | | Glutamine (mmol/l) | | | 0.99 (0.88, 1.12) | 0.90 |
|  | | | | | Glycine (mmol/l) | | | 0.94 (0.84, 1.05) | 0.25 |
|  | | | | | Histidine (mmol/l) | | | 0.99 (0.88, 1.11) | 0.89 |
|  | | | | | Isoleucine (mmol/l) | | | 1.01 (0.90, 1.14) | 0.84 |
|  | | | | | Leucine (mmol/l) | | | 0.97 (0.86, 1.10) | 0.62 |
| **METABOLITE** | | | | | | | | **HR (95% CI)** | **p-value** |
| **Amino acids** | | | | | | | |  |  |
|  | | | | | Valine (mmol/l) | | | 1.02 (0.90, 1.15) | 0.75 |
|  | | | | | Phenylalanine (mmol/l) | | | 1.01 (0.90, 1.14) | 0.84 |
|  | | | | | Tyrosine (mmol/l) | | | 0.98 (0.87, 1.09) | 0.69 |
| **Ketone bodies** | | | | | | | |  |  |
|  | | | | | Acetate (mmol/l) | | | 0.97 (0.86, 1.09) | 0.57 |
|  | | | | | Acetoacetate (mmol/l) | | | 1.11 (1.00, 1.23) | 0.06 |
|  | | | | | β-hydroxybutyrate (mmol/l) | | | 1.12 (1.00, 1.24) | 0.05 |
| **Fluid balance** | | | | | | | |  |  |
|  | | | | | | | Creatinine (mmol/l) | 0.91 (0.80, 1.05) | 0.21 |
|  | | | | | | | Albumin (signal area) | 0.94 (0.84, 1.05) | 0.27 |
| **Inflammation** | | | | | | | |  |  |
|  | | | | | | Glycoprotein acetyls, mainly a1-acid glycoprotein (mmol/l) | | 0.94 (0.84, 1.06) | 0.33 |

VLDL: very low density lipoproteins, IDL: intermediate-density lipoproteins, LDL: low density lipoproteins, HDL: high density lipoprotein.

Data are M (SD).

Cox proportional hazard regression adjusted for age, sex, education and ethnicity.

* Metabolites significantly associated at p<0.0002 (Bonferroni correction for multiple testing).

**Table S3. Association between 1-SD increment in metabolite concentrations and risk of dementia in analyses excluding participants with incomplete data on metabolites due to concentrations below the limit of quantification (N=5145).**

| **METABOLITE** | | | | | | | **HR (95% CI)** | **p-value** |
| --- | --- | --- | --- | --- | --- | --- | --- | --- |
| **Chylomicrons and extremely large VLDL** | | | | | | |  |  |
|  | | Concentration of chylomicrons and extremely large VLDL particles (mol/l) | | | | | 0.99 (0.87, 1.12) | 0.86 |
|  | | Total lipids in chylomicrons and extremely large VLDL (mmol/l) | | | | | 0.99 (0.87, 1.12) | 0.84 |
|  | | Phospholipids in chylomicrons and extremely large VLDL (mmol/l) | | | | | 0.98 (0.86, 1.12) | 0.78 |
|  | | Total cholesterol in chylomicrons and extremely large VLDL (mmol/l) | | | | | 0.97 (0.86, 1.10) | 0.67 |
|  | | Cholesterol esters in chylomicrons and extremely large VLDL (mmol/l) | | | | | 0.97 (0.86, 1.10) | 0.66 |
|  | | Free cholesterol in chylomicrons and extremely large VLDL (mmol/l) | | | | | 0.97 (0.85, 1.11) | 0.64 |
|  | | Triglycerides in chylomicrons and extremely large VLDL (mmol/l) | | | | | 0.99 (0.88, 1.12) | 0.91 |
| **Very large VLDL** | | | | | | |  |  |
|  | | | | Concentration of very large VLDL particles (mol/l) | | | 0.97 (0.86, 1.09) | 0.59 |
|  | | | | Total lipids in very large VLDL (mmol/l) | | | 0.97 (0.86, 1.09) | 0.62 |
|  | | | | Phospholipids in very large VLDL (mmol/l) | | | 0.97 (0.85, 1.09) | 0.59 |
|  | | | | Total cholesterol in very large VLDL (mmol/l) | | | 0.98 (0.87, 1.10) | 0.69 |
|  | | | | Cholesterol esters in very large VLDL (mmol/l) | | | 0.98 (0.87, 1.10) | 0.75 |
|  | | | | Free cholesterol in very large VLDL (mmol/l) | | | 0.98 (0.87, 1.11) | 0.74 |
|  | | | | Triglycerides in very large VLDL (mmol/l) | | | 0.96 (0.85, 1.08) | 0.49 |
| **Large VLDL** | | | | | | |  |  |
|  | | | Concentration of large VLDL particles (mol/l) | | | | 0.99 (0.87, 1.12) | 0.82 |
|  | | | Total lipids in large VLDL (mmol/l) | | | | 0.98 (0.87, 1.11) | 0.80 |
|  | | | Phospholipids in large VLDL (mmol/l) | | | | 0.99 (0.87, 1.12) | 0.85 |
|  | | | Total cholesterol in large VLDL (mmol/l) | | | | 0.97 (0.86, 1.10) | 0.62 |
|  | | | Cholesterol esters in large VLDL (mmol/l) | | | | 0.96 (0.85, 1.09) | 0.54 |
|  | | | Free cholesterol in large VLDL (mmol/l) | | | | 0.97 (0.86, 1.11) | 0.68 |
|  | | | Triglycerides in large VLDL (mmol/l) | | | | 0.99 (0.87, 1.12) | 0.86 |
| **Medium VLDL** | | | | | | |  |  |
|  | Concentration of medium VLDL particles (mol/l) | | | | | | 1 (0.88, 1.13) | 0.86 |
|  | Total lipids in medium VLDL (mmol/l) | | | | | | 1 (0.88, 1.13) | 0.96 |
| **METABOLITE** | | | | | | | **HR (95% CI)** | **p-value** |
| **Medium VLDL** | | | | | | |  |  |
|  | Phospholipids in medium VLDL (mmol/l) | | | | | | 1.00 (0.88, 1.14) | 0.99 |
|  | Total cholesterol in medium VLDL (mmol/l) | | | | | | 0.98 (0.87, 1.12) | 0.81 |
|  | Cholesterol esters in medium VLDL (mmol/l) | | | | | | 0.97 (0.86, 1.11) | 0.68 |
|  | Free cholesterol in medium VLDL (mmol/l) | | | | | | 0.99 (0.87, 1.13) | 0.92 |
|  | Triglycerides in medium VLDL (mmol/l) | | | | | | 1.00 (0.88, 1.14) | 0.99 |
| **Small VLDL** | | | | | | |  |  |
|  | | | | Concentration of small VLDL particles (mol/l) | | | 1.00 (0.88, 1.14) | 0.95 |
|  | | | | Total lipids in small VLDL (mmol/l) | | | 1.00 (0.88, 1.14) | 0.95 |
|  | | | | Phospholipids in small VLDL (mmol/l) | | | 1.00 (0.88, 1.13) | 0.98 |
|  | | | | Total cholesterol in small VLDL (mmol/l) | | | 1.01 (0.89, 1.14) | 0.92 |
|  | | | | Cholesterol esters in small VLDL (mmol/l) | | | 1.01 (0.89, 1.14) | 0.93 |
|  | | | | Free cholesterol in small VLDL (mmol/l) | | | 1.00 (0.88, 1.14) | 0.98 |
|  | | | | Triglycerides in small VLDL (mmol/l) | | | 1.00 (0.88, 1.13) | 0.98 |
| **Very small VLDL** | | | | | | |  |  |
|  | | | | | | Concentration of very small VLDL particles (mol/l) | 1.04 (0.92, 1.18) | 0.54 |
|  | | | | | | Total lipids in very small VLDL (mmol/l) | 1.04 (0.92, 1.18) | 0.55 |
|  | | | | | | Phospholipids in very small VLDL (mmol/l) | 1.04 (0.91, 1.17) | 0.57 |
|  | | | | | | Total cholesterol in very small VLDL (mmol/l) | 1.03 (0.91, 1.17) | 0.62 |
|  | | | | | | Cholesterol esters in very small VLDL (mmol/l) | 1.04 (0.92, 1.18) | 0.56 |
|  | | | | | | Free cholesterol in very small VLDL (mmol/l) | 1.02 (0.90, 1.16) | 0.76 |
|  | | | | | | Triglycerides in very small VLDL (mmol/l) | 1.02 (0.90, 1.15) | 0.77 |
| **IDL** | | | | | | |  |  |
|  | | | | Concentration of IDL particles (mol/l) | | | 1.03 (0.91, 1.17) | 0.64 |
|  | | | | Total lipids in IDL (mmol/l) | | | 1.03 (0.91, 1.16) | 0.69 |
|  | | | | Phospholipids in IDL (mmol/l) | | | 1.01 (0.90, 1.15) | 0.82 |
|  | | | | Total cholesterol in IDL (mmol/l) | | | 1.01 (0.90, 1.15) | 0.82 |
|  | | | | Cholesterol esters in IDL (mmol/l) | | | 1.02 (0.90, 1.15) | 0.79 |
|  | | | | Free cholesterol in IDL (mmol/l) | | | 1.01 (0.89, 1.14) | 0.87 |
|  | | | | Triglycerides in IDL (mmol/l) | | | 1.06 (0.94, 1.20) | 0.33 |
| **METABOLITE** | | | | | | | **HR (95% CI)** | **p-value** |
| **Large LDL** | | | | | | |  |  |
|  | | | Concentration of large LDL particles (mol/l) | | | | 1.03 (0.91, 1.16) | 0.67 |
|  | | | Total lipids in large LDL (mmol/l) | | | | 1.02 (0.90, 1.16) | 0.70 |
|  | | | Phospholipids in large LDL (mmol/l) | | | | 1.02 (0.90, 1.15) | 0.77 |
|  | | | Total cholesterol in large LDL (mmol/l) | | | | 1.02 (0.90, 1.15) | 0.81 |
|  | | | Cholesterol esters in large LDL (mmol/l) | | | | 1.02 (0.89, 1.15) | 0.82 |
|  | | | Free cholesterol in large LDL (mmol/l) | | | | 1.02 (0.90, 1.15) | 0.78 |
|  | | | Triglycerides in large LDL (mmol/l) | | | | 1.08 (0.95, 1.22) | 0.24 |
| **Medium LDL** | | | | | | |  |  |
|  | Concentration of medium LDL particles (mol/l) | | | | | | 1.03 (0.91, 1.16) | 0.66 |
|  | Total lipids in medium LDL (mmol/l) | | | | | | 1.03 (0.91, 1.16) | 0.67 |
|  | Phospholipids in medium LDL (mmol/l) | | | | | | 1.04 (0.92, 1.18) | 0.55 |
|  | | | | Total cholesterol in medium LDL (mmol/l) | | | 1.02 (0.90, 1.15) | 0.80 |
|  | | | | Cholesterol esters in medium LDL (mmol/l) | | | 1.01 (0.88, 1.15) | 0.89 |
|  | | | | Free cholesterol in medium LDL (mmol/l) | | | 1.04 (0.92, 1.17) | 0.56 |
|  | | | | Triglycerides in medium LDL (mmol/l) | | | 1.07 (0.94, 1.21) | 0.29 |
| **Small LDL** | | | | | | |  |  |
|  | | | | | Concentration of small LDL particles (mol/l) | | 1.03 (0.91, 1.17) | 0.62 |
|  | | | | | Total lipids in small LDL (mmol/l) | | 1.03 (0.91, 1.17) | 0.63 |
|  | | | | | Phospholipids in small LDL (mmol/l) | | 1.04 (0.92, 1.17) | 0.55 |
|  | | | | | Total cholesterol in small LDL (mmol/l) | | 1.02 (0.90, 1.16) | 0.73 |
|  | | | | | Cholesterol esters in small LDL (mmol/l) | | 1.02 (0.89, 1.16) | 0.82 |
|  | | | | | Free cholesterol in small LDL (mmol/l) | | 1.04 (0.92, 1.18) | 0.51 |
|  | | | | | Triglycerides in small LDL (mmol/l) | | 1.04 (0.91, 1.17) | 0.57 |
| **Very large HDL** | | | | | | |  |  |
|  | | Concentration of very large HDL particles (mol/l) | | | | | 1.05 (0.93, 1.19) | 0.44 |
|  | | Total lipids in very large HDL (mmol/l) | | | | | 1.05 (0.93, 1.19) | 0.44 |
|  | | Phospholipids in very large HDL (mmol/l) | | | | | 1.05 (0.92, 1.19) | 0.48 |
|  | | Total cholesterol in very large HDL (mmol/l) | | | | | 1.05 (0.93, 1.19) | 0.45 |
|  | | Cholesterol esters in very large HDL (mmol/l) | | | | | 1.05 (0.93, 1.19) | 0.45 |
| **METABOLITE** | | | | | | | **HR (95% CI)** | **p-value** |
| **Very large HDL** | | | | | | |  |  |
|  | | Free cholesterol in very large HDL (mmol/l) | | | | | 1.05 (0.92, 1.19) | 0.49 |
|  | | Triglycerides in very large HDL (mmol/l) | | | | | 1.02 (0.89, 1.16) | 0.79 |
| **Large HDL** | | | | | | |  |  |
|  | | Concentration of large HDL particles (mol/l) | | | | | 1.00 (0.88, 1.13) | 0.97 |
|  | | Total lipids in large HDL (mmol/l) | | | | | 1.00 (0.89, 1.14) | 0.97 |
|  | | Phospholipids in large HDL (mmol/l) | | | | | 1.00 (0.88, 1.13) | 0.97 |
|  | | Total cholesterol in large HDL (mmol/l) | | | | | 1.01 (0.89, 1.14) | 0.91 |
|  | | Cholesterol esters in large HDL (mmol/l) | | | | | 1.01 (0.89, 1.14) | 0.92 |
|  | | Free cholesterol in large HDL (mmol/l) | | | | | 1.01 (0.89, 1.14) | 0.89 |
|  | | Triglycerides in large HDL (mmol/l) | | | | | 1.00 (0.88, 1.14) | 0.99 |
| **Medium HDL** | | | | | | |  |  |
|  | | | Concentration of medium HDL particles (mol/l) | | | | 0.94 (0.84, 1.06) | 0.34 |
|  | | | Total lipids in medium HDL (mmol/l) | | | | 0.94 (0.84, 1.06) | 0.34 |
|  | | | Phospholipids in medium HDL (mmol/l) | | | | 0.97 (0.86, 1.09) | 0.56 |
|  | | | Total cholesterol in medium HDL (mmol/l) | | | | 0.93 (0.83, 1.05) | 0.25 |
|  | | | Cholesterol esters in medium HDL (mmol/l) | | | | 0.93 (0.83, 1.04) | 0.21 |
|  | | | Free cholesterol in medium HDL (mmol/l) | | | | 0.95 (0.85, 1.07) | 0.44 |
|  | | | Triglycerides in medium HDL (mmol/l) | | | | 0.93 (0.82, 1.05) | 0.27 |
| **Small HDL** | | | | | | |  |  |
|  | | | Concentration of small HDL particles (mol/l) | | | | 0.97 (0.87, 1.09) | 0.60 |
|  | | | Total lipids in small HDL (mmol/l) | | | | 0.97 (0.87, 1.09) | 0.60 |
|  | | | Phospholipids in small HDL (mmol/l) | | | | 0.95 (0.85, 1.06) | 0.39 |
|  | | | Total cholesterol in small HDL (mmol/l) | | | | 0.99 (0.89, 1.12) | 0.93 |
|  | | | Cholesterol esters in small HDL (mmol/l) | | | | 1.01 (0.90, 1.13) | 0.92 |
|  | | | Free cholesterol in small HDL (mmol/l) | | | | 0.96 (0.86, 1.07) | 0.46 |
|  | | | Triglycerides in small HDL (mmol/l) | | | | 1.02 (0.90, 1.16) | 0.73 |
| **Chylomicrons and extremely large VLDL ratios** | | | | | | |  |  |
|  | | Phospholipids to total lipids ratio in chylomicrons and extremely large VLDL (%) | | | | | 0.94 (0.78, 1.13) | 0.50 |
|  | | Total cholesterol to total lipids ratio in chylomicrons and extremely large VLDL (%) | | | | | 0.89 (0.77, 1.03) | 0.12 |
| **METABOLITE** | | | | | | | **HR (95% CI)** | **p-value** |
| **Chylomicrons and extremely large VLDL ratios** | | | | | | |  |  |
|  | | Cholesterol esters to total lipids ratio in chylomicrons and extremely large VLDL (%) | | | | | 0.93 (0.80, 1.07) | 0.29 |
|  | | Free cholesterol to total lipids ratio in chylomicrons and extremely large VLDL (%) | | | | | 0.91 (0.77, 1.07) | 0.24 |
|  | | Triglycerides to total lipids ratio in chylomicrons and extremely large VLDL (%) | | | | | 1.11 (0.97, 1.27) | 0.12 |
| **Very large VLDL ratios** | | | | | | |  |  |
|  | | | Phospholipids to total lipids ratio in very large VLDL (%) | | | | 1.00 (0.86, 1.16) | 0.97 |
|  | | | Total cholesterol to total lipids ratio in very large VLDL (%) | | | | 1.04 (0.92, 1.17) | 0.55 |
|  | | | Cholesterol esters to total lipids ratio in very large VLDL (%) | | | | 1.03 (0.91, 1.18) | 0.62 |
|  | | | Free cholesterol to total lipids ratio in very large VLDL (%) | | | | 1.05 (0.93, 1.18) | 0.45 |
|  | | | Triglycerides to total lipids ratio in very large VLDL (%) | | | | 0.93 (0.82, 1.06) | 0.29 |
| **Large VLDL ratios** | | | | | | |  |  |
|  | | | | Phospholipids to total lipids ratio in large VLDL (%) | | | 1.07 (0.93, 1.24) | 0.33 |
|  | | | | Total cholesterol to total lipids ratio in large VLDL (%) | | | 0.93 (0.80, 1.07) | 0.29 |
|  | | | | Cholesterol esters to total lipids ratio in large VLDL (% | | | 0.96 (0.84, 1.09) | 0.51 |
|  | | | | Free cholesterol to total lipids ratio in large VLDL (%) | | | 0.94 (0.81, 1.09) | 0.42 |
|  | | | | Triglycerides to total lipids ratio in large VLDL (%) | | | 1.05 (0.88, 1.24) | 0.61 |
| **Medium VLDL ratios** | | | | | | |  |  |
|  | | | | | Phospholipids to total lipids ratio in medium VLDL (%) | | 1.06 (0.93, 1.20) | 0.37 |
|  | | | | | Total cholesterol to total lipids ratio in medium VLDL (%) | | 0.96 (0.84, 1.10) | 0.56 |
|  | | | | | Cholesterol esters to total lipids ratio in medium VLDL (%) | | 0.96 (0.85, 1.09) | 0.56 |
|  | | | | | Free cholesterol to total lipids ratio in medium VLDL (%) | | 0.98 (0.84, 1.14) | 0.75 |
|  | | | | | Triglycerides to total lipids ratio in medium VLDL (%) | | 1.02 (0.87, 1.21) | 0.78 |
| **Small VLDL ratios** | | | | | | |  |  |
|  | | | Phospholipids to total lipids ratio in small VLDL (%) | | | | 0.96 (0.84, 1.09) | 0.49 |
|  | | | Total cholesterol to total lipids ratio in small VLDL (%) | | | | 1.00 (0.89, 1.13) | 0.96 |
|  | | | Cholesterol esters to total lipids ratio in small VLDL (%) | | | | 1.00 (0.88, 1.13) | 0.99 |
|  | | | | | Free cholesterol to total lipids ratio in small VLDL (%) | | 0.98 (0.86, 1.11) | 0.74 |
|  | | | | | Triglycerides to total lipids ratio in small VLDL (%) | | 0.99 (0.87, 1.12) | 0.82 |
| **Very small VLDL ratios** | | | | | | |  |  |
|  | | | Phospholipids to total lipids ratio in very small VLDL (%) | | | | 1.00 (0.88, 1.13) | 0.94 |
| **METABOLITE** | | | | | | | **HR (95% CI)** | **p-value** |
| **Very small VLDL ratios** | | | | | | |  |  |
|  | | | Total cholesterol to total lipids ratio in very small VLDL (%) | | | | 0.98 (0.86, 1.11) | 0.72 |
|  | | | Cholesterol esters to total lipids ratio in very small VLDL (%) | | | | 1.00 (0.88, 1.13) | 0.95 |
|  | | | Free cholesterol to total lipids ratio in very small VLDL (%) | | | | 0.94 (0.83, 1.06) | 0.31 |
|  | | | Triglycerides to total lipids ratio in very small VLDL (%) | | | | 0.99 (0.88, 1.12) | 0.88 |
| **IDL ratios** | | | | | | |  |  |
|  | | | | | Phospholipids to total lipids ratio in IDL (%) | | 0.91 (0.80, 1.03) | 0.15 |
|  | | | | | Total cholesterol to total lipids ratio in IDL (%) | | 0.95 (0.84, 1.08) | 0.44 |
|  | | | | | Cholesterol esters to total lipids ratio in IDL (%) | | 0.97 (0.86, 1.10) | 0.62 |
|  | | | | | Free cholesterol to total lipids ratio in IDL (%) | | 0.94 (0.83, 1.06) | 0.30 |
|  | | | | | Triglycerides to total lipids ratio in IDL (%) | | 1.04 (0.93, 1.17) | 0.48 |
| **Large LDL ratios** | | | | | | |  |  |
|  | | | | Phospholipids to total lipids ratio in large LDL (%) | | | 0.95 (0.83, 1.08) | 0.44 |
|  | | | | Total cholesterol to total lipids ratio in large LDL (%) | | | 0.95 (0.83, 1.09) | 0.49 |
|  | | | | Cholesterol esters to total lipids ratio in large LDL (%) | | | 0.96 (0.81, 1.13) | 0.60 |
|  | | | | Free cholesterol to total lipids ratio in large LDL (%) | | | 0.95 (0.84, 1.08) | 0.46 |
|  | | | | Triglycerides to total lipids ratio in large LDL (%) | | | 1.05 (0.94, 1.19) | 0.38 |
| **Medium LDL ratios** | | | | | | |  |  |
|  | | | | | | Phospholipids to total lipids ratio in medium LDL (%) | 1.00 (0.89, 1.13) | 0.94 |
|  | | | | | | Total cholesterol to total lipids ratio in medium LDL (%) | 0.97 (0.85, 1.10) | 0.61 |
|  | | | | | | Cholesterol esters to total lipids ratio in medium LDL (%) | 0.96 (0.81, 1.12) | 0.58 |
|  | | | | | | Free cholesterol to total lipids ratio in medium LDL (%) | 1.01 (0.88, 1.15) | 0.91 |
|  | | | | | | Triglycerides to total lipids ratio in medium LDL (%) | 1.04 (0.92, 1.17) | 0.50 |
| **Small LDL ratios** | | | | | | |  |  |
|  | | | | | Phospholipids to total lipids ratio in small LDL (%) | | 0.99 (0.87, 1.12) | 0.85 |
|  | | | | | Total cholesterol to total lipids ratio in small LDL (%) | | 0.99 (0.87, 1.12) | 0.83 |
|  | | | | | Cholesterol esters to total lipids ratio in small LDL (%) | | 0.98 (0.84, 1.14) | 0.78 |
|  | | | | | Free cholesterol to total lipids ratio in small LDL (%) | | 1.01 (0.89, 1.15) | 0.87 |
|  | | | | | Triglycerides to total lipids ratio in small LDL (%) | | 1.01 (0.90, 1.14) | 0.85 |
| **METABOLITE** | | | | | | | **HR (95% CI)** | **p-value** |
| **Very large HDL ratios** | | | | | | |  |  |
|  | | | | | Phospholipids to total lipids ratio in very large HDL (%) | | 1.00 (0.89, 1.14) | 0.96 |
|  | | | | | Total cholesterol to total lipids ratio in very large HDL (%) | | 1.00 (0.88, 1.14) | 0.98 |
|  | | | | | Cholesterol esters to total lipids ratio in very large HDL (%) | | 1.00 (0.88, 1.15) | 0.96 |
|  | | | | | Free cholesterol to total lipids ratio in very large HDL (%) | | 0.99 (0.87, 1.12) | 0.86 |
|  | | | | | Triglycerides to total lipids ratio in very large HDL (%) | | 0.98 (0.86, 1.11) | 0.71 |
| **Large HDL ratios** | | | | | | |  |  |
|  | | | | | Phospholipids to total lipids ratio in large HDL (%) | | 0.97 (0.86, 1.09) | 0.61 |
|  | | | | | Total cholesterol to total lipids ratio in large HDL (%) | | 1.03 (0.92, 1.16) | 0.60 |
|  | | | | | Cholesterol esters to total lipids ratio in large HDL (%) | | 1.03 (0.91, 1.16) | 0.61 |
|  | | | | | Free cholesterol to total lipids ratio in large HDL (%) | | 1.03 (0.91, 1.16) | 0.67 |
|  | | | | | Triglycerides to total lipids ratio in large HDL (%) | | 1.00 (0.88, 1.13) | 0.96 |
| **Medium HDL ratios** | | | | | | |  |  |
|  | | | | | | Phospholipids to total lipids ratio in medium HDL (%) | 1.17 (1.04, 1.32) | **0.01** |
|  | | | | | | Total cholesterol to total lipids ratio in medium HDL (%) | 0.93 (0.82, 1.04) | 0.19 |
|  | | | | | | Cholesterol esters to total lipids ratio in medium HDL (%) | 0.92 (0.82, 1.03) | 0.17 |
|  | | | | | | Free cholesterol to total lipids ratio in medium HDL (%) | 1.00 (0.88, 1.13) | 0.94 |
|  | | | | | | Triglycerides to total lipids ratio in medium HDL (%) | 0.98 (0.87, 1.11) | 0.73 |
| **Small HDL ratios** | | | | | | |  |  |
|  | | | | | Phospholipids to total lipids ratio in small HDL (%) | | 0.94 (0.84, 1.06) | 0.32 |
|  | | | | | Total cholesterol to total lipids ratio in small HDL (%) | | 1.03 (0.92, 1.16) | 0.59 |
|  | | | | | Cholesterol esters to total lipids ratio in small HDL (%) | | 1.03 (0.92, 1.16) | 0.58 |
|  | | | | | Free cholesterol to total lipids ratio in small HDL (%) | | 0.95 (0.85, 1.06) | 0.33 |
|  | | | | | Triglycerides to total lipids ratio in small HDL (%) | | 1.04 (0.92, 1.18) | 0.55 |
| **Lipoprotein particle sizes** | | | | | | |  |  |
|  | | | | Mean diameter for VLDL particles (nm) | | | 0.98 (0.87, 1.11) | 0.79 |
|  | | | | Mean diameter for LDL particles (nm) | | | 0.99 (0.87, 1.12) | 0.82 |
|  | | | | Mean diameter for HDL particles (nm) | | | 1.02 (0.90, 1.15) | 0.78 |

| **METABOLITE** | | | | | | | | **HR (95% CI)** | **p-value** |
| --- | --- | --- | --- | --- | --- | --- | --- | --- | --- |
| **Cholesterol related metabolites** | | | | | | | |  |  |
|  | | Serum total cholesterol (mmol/l) | | | | | | 1.00 (0.89, 1.13) | 0.96 |
|  | | Total cholesterol in VLDL (mmol/l) | | | | | | 1.01 (0.89, 1.15) | 0.86 |
|  | | Remnant cholesterol (non-HDL, non-LDL -cholesterol) (mmol/l) | | | | | | 1.02 (0.90, 1.16) | 0.77 |
|  | | Total cholesterol in LDL (mmol/l) | | | | | | 1.02 (0.90, 1.15) | 0.79 |
|  | | Total cholesterol in HDL (mmol/l) | | | | | | 0.99 (0.87, 1.11) | 0.82 |
|  | | Total cholesterol in HDL2 (mmol/l) | | | | | | 0.98 (0.87, 1.11) | 0.74 |
|  | | Total cholesterol in HDL3 (mmol/l) | | | | | | 1.06 (0.94, 1.20) | 0.31 |
|  | | Esterified cholesterol (mmol/l) | | | | | | 1.01 (0.89, 1.14) | 0.87 |
|  | | Free cholesterol (mmol/l) | | | | | | 0.98 (0.87, 1.11) | 0.81 |
| **Lipids related metabolites** | | | | | | | |  |  |
|  | Serum total triglycerides (mmol/l) | | | | | | | 1.01 (0.89, 1.14) | 0.89 |
|  | | | Triglycerides in VLDL (mmol/l) | | | | | 1.00 (0.88, 1.14) | 0.99 |
|  | | | Triglycerides in LDL (mmol/l) | | | | | 1.07 (0.94, 1.21) | 0.30 |
|  | | | Triglycerides in HDL (mmol/l) | | | | | 0.98 (0.86, 1.11) | 0.72 |
|  | | | Diacylglycerol (mmol/l) | | | | | 0.99 (0.88, 1.11) | 0.88 |
|  | | | Ratio of diacylglycerol to triglycerides | | | | | 0.99 (0.89, 1.11) | 0.93 |
|  | | | Total phosphoglycerides (mmol/l) | | | | | 0.98 (0.87, 1.11) | 0.78 |
|  | | | Ratio of triglycerides to phosphoglycerides | | | | | 1.00 (0.88, 1.12) | 0.94 |
|  | | | Phosphatidylcholine and other cholines (mmol/l) | | | | | 0.98 (0.86, 1.11) | 0.77 |
|  | | | Sphingomyelins (mmol/l) | | | | | 0.92 (0.82, 1.04) | 0.18 |
|  | | | Total cholines (mmol/l) | | | | | 0.97 (0.86, 1.10) | 0.67 |
| **Fatty acids related metabolites** | | | | | | | |  |  |
|  | | Total fatty acids (mmol/l) | | | | | | 1.01 (0.89, 1.14) | 0.88 |
|  | | Estimated description of fatty acid chain length, not actual carbon number | | | | | | 0.99 (0.88, 1.12) | 0.91 |
|  | | Unsaturation degree | | | | | | 0.94 (0.83, 1.06) | 0.30 |
|  | | 22:6, docosahexaenoic acid (mmol/l) | | | | | | 0.98 (0.87, 1.10) | 0.72 |
|  | | 18:2, linoleic acid (mmol/l) | | | | | | 0.95 (0.84, 1.07) | 0.36 |
|  | | Conjugated linoleic acid (mmol/l) | | | | | | 0.98 (0.87, 1.10) | 0.72 |
|  | | Omega-3 fatty acids (mmol/l) | | | | | | 0.99 (0.88, 1.12) | 0.90 |
| **METABOLITE** | | | | | | | | **HR (95% CI)** | **p-value** |
| **Fatty acids related metabolites** | | | | | | | |  |  |
|  | | Omega-6 fatty acids (mmol/l) | | | | | | 0.95 (0.84, 1.07) | 0.37 |
|  | | Polyunsaturated fatty acids (mmol/l) | | | | | | 0.95 (0.84, 1.08) | 0.44 |
|  | | Monounsaturated fatty acids; 16:1, 18:1 (mmol/l) | | | | | | 1.04 (0.92, 1.17) | 0.58 |
|  | | Saturated fatty acids (mmol/l) | | | | | | 1.02 (0.91, 1.16) | 0.69 |
|  | | Ratio of 22:6 docosahexaenoic acid to total fatty acids (%) | | | | | | 0.97 (0.86, 1.09) | 0.62 |
|  | | Ratio of 18:2 linoleic acid to total fatty acids (%) | | | | | | 0.92 (0.82, 1.03) | 0.15 |
|  | | Ratio of conjugated linoleic acid to total fatty acids (%) | | | | | | 0.97 (0.87, 1.09) | 0.64 |
|  | | Ratio of omega-3 fatty acids to total fatty acids (%) | | | | | | 0.98 (0.88, 1.11) | 0.79 |
|  | | Ratio of omega-6 fatty acids to total fatty acids (%) | | | | | | 0.91 (0.81, 1.02) | 0.09 |
|  | | Ratio of polyunsaturated fatty acids to total fatty acids (%) | | | | | | 0.91 (0.81, 1.02) | 0.10 |
|  | | Ratio of monounsaturated fatty acids to total fatty acids (%) | | | | | | 1.06 (0.93, 1.19) | 0.38 |
|  | | Ratio of saturated fatty acids to total fatty acids (%) | | | | | | 1.05 (0.94, 1.18) | 0.37 |
| **Apoliporproteins related metabolites** | | | | | | | |  |  |
|  | | Apolipoprotein a-i (g/l) | | | | | | 0.97 (0.86, 1.10) | 0.64 |
|  | | Apolipoprotein b (g/l) | | | | | | 1.03 (0.91, 1.16) | 0.68 |
|  | | Ratio of apolipoprotein b to apolipoprotein a-i | | | | | | 1.04 (0.92, 1.18) | 0.52 |
| **Glycolisis related metabolites** | | | | | | | |  |  |
|  | | Glucose (mmol/l) | | | | | | 1.24 (1.13, 1.37) | **0.00001*** |
|  | | | | Lactate (mmol/l) | | | | 0.98 (0.88, 1.10) | 0.74 |
|  | | | | Pyruvate (mmol/l) | | | | 1.05 (0.94, 1.17) | 0.35 |
|  | | | | Citrate (mmol/l) | | | | 1.08 (0.96, 1.21) | 0.21 |
|  | | | | Glycerol (mmol/l) | | | | 1.05 (0.92, 1.19) | 0.50 |
| **Amino acids** | | | | | | | |  |  |
|  | | | | | Alanine (mmol/l) | | | 0.94 (0.84, 1.05) | 0.29 |
|  | | | | | Glutamine (mmol/l) | | | 0.98 (0.86, 1.11) | 0.73 |
|  | | | | | Glycine (mmol/l) | | | 0.94 (0.84, 1.06) | 0.33 |
|  | | | | | Histidine (mmol/l) | | | 0.99 (0.88, 1.11) | 0.85 |
|  | | | | | Isoleucine (mmol/l) | | | 1.04 (0.92, 1.17) | 0.57 |
|  | | | | | Leucine (mmol/l) | | | 0.99 (0.88, 1.13) | 0.94 |
| **METABOLITE** | | | | | | | | **HR (95% CI)** | **p-value** |
| **Amino acids** | | | | | | | |  |  |
|  | | | | | Valine (mmol/l) | | | 1.04 (0.92, 1.18) | 0.55 |
|  | | | | | Phenylalanine (mmol/l) | | | 1.02 (0.91, 1.15) | 0.73 |
|  | | | | | Tyrosine (mmol/l) | | | 0.98 (0.87, 1.09) | 0.68 |
| **Ketone bodies** | | | | | | | |  |  |
|  | | | | | Acetate (mmol/l) | | | 0.97 (0.86, 1.09) | 0.61 |
|  | | | | | Acetoacetate (mmol/l) | | | 1.09 (0.98, 1.22) | 0.13 |
|  | | | | | β-hydroxybutyrate (mmol/l) | | | 1.09 (0.97, 1.22) | 0.13 |
| **Fluid balance** | | | | | | | |  |  |
|  | | | | | | | Creatinine (mmol/l) | 0.90 (0.78, 1.04) | 0.15 |
|  | | | | | | | Albumin (signal area) | 0.93 (0.83, 1.05) | 0.24 |
| **Inflammation** | | | | | | | |  |  |
|  | | | | | | Glycoprotein acetyls, mainly a1-acid glycoprotein (mmol/l) | | 0.96 (0.85, 1.08) | 0.47 |

VLDL: very low density lipoproteins, IDL: intermediate-density lipoproteins, LDL: low density lipoproteins, HDL: high density lipoprotein.

Data are M (SD).

Cox proportional hazard regression adjusted for age, sex, education and ethnicity.

* Metabolites significantly associated at p<0.0002 (Bonferroni correction for multiple testing).

**Table S4. Association between 1-SD increment in metabolite concentrations and risk of dementia including participants with incomplete data on metabolites due to outlier values (≥±9 SD) in metabolite concentrations (N=5446).**

| **METABOLITE** | | | | | | | **HR (95% CI)** | **p-value** |
| --- | --- | --- | --- | --- | --- | --- | --- | --- |
| **Chylomicrons and extremely large VLDL** | | | | | | |  |  |
|  | | Concentration of chylomicrons and extremely large VLDL particles (mol/l) | | | | | 0.93 (0.77, 1.13) | 0.47 |
|  | | Total lipids in chylomicrons and extremely large VLDL (mmol/l) | | | | | 0.93 (0.76, 1.13) | 0.45 |
|  | | Phospholipids in chylomicrons and extremely large VLDL (mmol/l) | | | | | 0.92 (0.78, 1.09) | 0.35 |
|  | | Total cholesterol in chylomicrons and extremely large VLDL (mmol/l) | | | | | 0.90 (0.76, 1.07) | 0.23 |
|  | | Cholesterol esters in chylomicrons and extremely large VLDL (mmol/l) | | | | | 0.90 (0.75, 1.07) | 0.23 |
|  | | Free cholesterol in chylomicrons and extremely large VLDL (mmol/l) | | | | | 0.90 (0.77, 1.05) | 0.19 |
|  | | Triglycerides in chylomicrons and extremely large VLDL (mmol/l) | | | | | 0.94 (0.77, 1.15) | 0.54 |
| **Very large VLDL** | | | | | | |  |  |
|  | | | | Concentration of very large VLDL particles (mol/l) | | | 0.89 (0.72, 1.11) | 0.30 |
|  | | | | Total lipids in very large VLDL (mmol/l) | | | 0.86 (0.65, 1.14) | 0.30 |
|  | | | | Phospholipids in very large VLDL (mmol/l) | | | 0.85 (0.67, 1.08) | 0.18 |
|  | | | | Total cholesterol in very large VLDL (mmol/l) | | | 0.82 (0.62, 1.09) | 0.18 |
|  | | | | Cholesterol esters in very large VLDL (mmol/l) | | | 0.86 (0.69, 1.08) | 0.19 |
|  | | | | Free cholesterol in very large VLDL (mmol/l) | | | 0.84 (0.64, 1.10) | 0.20 |
|  | | | | Triglycerides in very large VLDL (mmol/l) | | | 0.88 (0.71, 1.09) | 0.25 |
| **Large VLDL** | | | | | | |  |  |
|  | | | Concentration of large VLDL particles (mol/l) | | | | 0.95 (0.79, 1.13) | 0.55 |
|  | | | Total lipids in large VLDL (mmol/l) | | | | 0.91 (0.67, 1.22) | 0.53 |
|  | | | Phospholipids in large VLDL (mmol/l) | | | | 0.92 (0.70, 1.20) | 0.54 |
|  | | | Total cholesterol in large VLDL (mmol/l) | | | | 0.86 (0.64, 1.16) | 0.32 |
|  | | | Cholesterol esters in large VLDL (mmol/l) | | | | 0.84 (0.60, 1.16) | 0.29 |
|  | | | Free cholesterol in large VLDL (mmol/l) | | | | 0.90 (0.72, 1.11) | 0.31 |
|  | | | Triglycerides in large VLDL (mmol/l) | | | | 0.94 (0.72, 1.22) | 0.62 |
| **Medium VLDL** | | | | | | |  |  |
|  | Concentration of medium VLDL particles (mol/l) | | | | | | 0.97 (0.86, 1.10) | 0.63 |
|  | Total lipids in medium VLDL (mmol/l) | | | | | | 0.96 (0.83, 1.12) | 0.61 |
| **METABOLITE** | | | | | | | **HR (95% CI)** | **p-value** |
| **Medium VLDL** | | | | | | |  |  |
|  | Phospholipids in medium VLDL (mmol/l) | | | | | | 0.96 (0.83, 1.12) | 0.63 |
|  | Total cholesterol in medium VLDL (mmol/l) | | | | | | 0.94 (0.80, 1.10) | 0.42 |
|  | Cholesterol esters in medium VLDL (mmol/l) | | | | | | 0.92 (0.78, 1.08) | 0.31 |
|  | Free cholesterol in medium VLDL (mmol/l) | | | | | | 0.96 (0.83, 1.10) | 0.53 |
|  | Triglycerides in medium VLDL (mmol/l) | | | | | | 0.98 (0.84, 1.13) | 0.73 |
| **Small VLDL** | | | | | | |  |  |
|  | | | | Concentration of small VLDL particles (mol/l) | | | 0.97 (0.86, 1.10) | 0.67 |
|  | | | | Total lipids in small VLDL (mmol/l) | | | 0.96 (0.81, 1.14) | 0.66 |
|  | | | | Phospholipids in small VLDL (mmol/l) | | | 0.96 (0.81, 1.13) | 0.63 |
|  | | | | Total cholesterol in small VLDL (mmol/l) | | | 0.96 (0.80, 1.14) | 0.62 |
|  | | | | Cholesterol esters in small VLDL (mmol/l) | | | 0.96 (0.80, 1.14) | 0.62 |
|  | | | | Free cholesterol in small VLDL (mmol/l) | | | 0.96 (0.82, 1.12) | 0.60 |
|  | | | | Triglycerides in small VLDL (mmol/l) | | | 0.97 (0.84, 1.12) | 0.68 |
| **Very small VLDL** | | | | | | |  |  |
|  | | | | | | Concentration of very small VLDL particles (mol/l) | 1.00 (0.88, 1.13) | 0.99 |
|  | | | | | | Total lipids in very small VLDL (mmol/l) | 1.00 (0.80, 1.25) | 0.99 |
|  | | | | | | Phospholipids in very small VLDL (mmol/l) | 0.99 (0.81, 1.21) | 0.91 |
|  | | | | | | Total cholesterol in very small VLDL (mmol/l) | 1.00 (0.81, 1.25) | 0.98 |
|  | | | | | | Cholesterol esters in very small VLDL (mmol/l) | 1.02 (0.82, 1.27) | 0.85 |
|  | | | | | | Free cholesterol in very small VLDL (mmol/l) | 0.97 (0.80, 1.17) | 0.74 |
|  | | | | | | Triglycerides in very small VLDL (mmol/l) | 0.98 (0.84, 1.15) | 0.81 |
| **IDL** | | | | | | |  |  |
|  | | | | Concentration of IDL particles (mol/l) | | | 0.99 (0.88, 1.11) | 0.87 |
|  | | | | Total lipids in IDL (mmol/l) | | | 0.99 (0.88, 1.11) | 0.83 |
|  | | | | Phospholipids in IDL (mmol/l) | | | 0.96 (0.75, 1.21) | 0.71 |
|  | | | | Total cholesterol in IDL (mmol/l) | | | 0.97 (0.77, 1.21) | 0.76 |
|  | | | | Cholesterol esters in IDL (mmol/l) | | | 0.97 (0.78, 1.21) | 0.80 |
|  | | | | Free cholesterol in IDL (mmol/l) | | | 0.96 (0.78, 1.18) | 0.70 |
|  | | | | Triglycerides in IDL (mmol/l) | | | 1.03 (0.85, 1.25) | 0.74 |
| **METABOLITE** | | | | | | | **HR (95% CI)** | **p-value** |
| **Large LDL** | | | | | | |  |  |
|  | | | Concentration of large LDL particles (mol/l) | | | | 0.99 (0.88, 1.11) | 0.83 |
|  | | | Total lipids in large LDL (mmol/l) | | | | 0.97 (0.77, 1.23) | 0.81 |
|  | | | Phospholipids in large LDL (mmol/l) | | | | 0.96 (0.75, 1.23) | 0.75 |
|  | | | Total cholesterol in large LDL (mmol/l) | | | | 0.97 (0.78, 1.20) | 0.75 |
|  | | | Cholesterol esters in large LDL (mmol/l) | | | | 0.97 (0.79, 1.18) | 0.73 |
|  | | | Free cholesterol in large LDL (mmol/l) | | | | 0.97 (0.78, 1.21) | 0.78 |
|  | | | Triglycerides in large LDL (mmol/l) | | | | 1.05 (0.86, 1.29) | 0.64 |
| **Medium LDL** | | | | | | |  |  |
|  | Concentration of medium LDL particles (mol/l) | | | | | | 0.99 (0.88, 1.11) | 0.83 |
|  | Total lipids in medium LDL (mmol/l) | | | | | | 0.98 (0.77, 1.23) | 0.84 |
|  | Phospholipids in medium LDL (mmol/l) | | | | | | 0.99 (0.76, 1.30) | 0.97 |
|  | | | | Total cholesterol in medium LDL (mmol/l) | | | 0.97 (0.79, 1.19) | 0.76 |
|  | | | | Cholesterol esters in medium LDL (mmol/l) | | | 0.96 (0.80, 1.16) | 0.70 |
|  | | | | Free cholesterol in medium LDL (mmol/l) | | | 1.00 (0.78, 1.30) | 0.98 |
|  | | | | Triglycerides in medium LDL (mmol/l) | | | 1.04 (0.85, 1.27) | 0.73 |
| **Small LDL** | | | | | | |  |  |
|  | | | | | Concentration of small LDL particles (mol/l) | | 0.99 (0.88, 1.12) | 0.87 |
|  | | | | | Total lipids in small LDL (mmol/l) | | 0.98 (0.79, 1.23) | 0.87 |
|  | | | | | Phospholipids in small LDL (mmol/l) | | 0.99 (0.75, 1.31) | 0.96 |
|  | | | | | Total cholesterol in small LDL (mmol/l) | | 0.98 (0.81, 1.18) | 0.83 |
|  | | | | | Cholesterol esters in small LDL (mmol/l) | | 0.98 (0.83, 1.16) | 0.79 |
|  | | | | | Free cholesterol in small LDL (mmol/l) | | 1.01 (0.80, 1.28) | 0.93 |
|  | | | | | Triglycerides in small LDL (mmol/l) | | 0.98 (0.83, 1.17) | 0.85 |
| **Very large HDL** | | | | | | |  |  |
|  | | Concentration of very large HDL particles (mol/l) | | | | | 1.04 (0.92, 1.17) | 0.55 |
|  | | Total lipids in very large HDL (mmol/l) | | | | | 1.07 (0.86, 1.34) | 0.54 |
|  | | Phospholipids in very large HDL (mmol/l) | | | | | 1.06 (0.86, 1.30) | 0.58 |
|  | | Total cholesterol in very large HDL (mmol/l) | | | | | 1.08 (0.87, 1.33) | 0.50 |
|  | | Cholesterol esters in very large HDL (mmol/l) | | | | | 1.08 (0.88, 1.32) | 0.47 |
| **METABOLITE** | | | | | | | **HR (95% CI)** | **p-value** |
| **Very large HDL** | | | | | | |  |  |
|  | | Free cholesterol in very large HDL (mmol/l) | | | | | 1.06 (0.87, 1.28) | 0.59 |
|  | | Triglycerides in very large HDL (mmol/l) | | | | | 0.94 (0.80, 1.11) | 0.47 |
| **Large HDL** | | | | | | |  |  |
|  | | Concentration of large HDL particles (mol/l) | | | | | 0.98 (0.87, 1.10) | 0.73 |
|  | | Total lipids in large HDL (mmol/l) | | | | | 0.97 (0.81, 1.16) | 0.74 |
|  | | Phospholipids in large HDL (mmol/l) | | | | | 0.96 (0.80, 1.15) | 0.65 |
|  | | Total cholesterol in large HDL (mmol/l) | | | | | 0.98 (0.83, 1.16) | 0.86 |
|  | | Cholesterol esters in large HDL (mmol/l) | | | | | 0.98 (0.83, 1.16) | 0.86 |
|  | | Free cholesterol in large HDL (mmol/l) | | | | | 0.98 (0.85, 1.14) | 0.84 |
|  | | Triglycerides in large HDL (mmol/l) | | | | | 0.95 (0.82, 1.10) | 0.48 |
| **Medium HDL** | | | | | | |  |  |
|  | | | Concentration of medium HDL particles (mol/l) | | | | 0.92 (0.82, 1.03) | 0.14 |
|  | | | Total lipids in medium HDL (mmol/l) | | | | 0.81 (0.61, 1.08) | 0.14 |
|  | | | Phospholipids in medium HDL (mmol/l) | | | | 0.86 (0.65, 1.13) | 0.28 |
|  | | | Total cholesterol in medium HDL (mmol/l) | | | | 0.82 (0.64, 1.05) | 0.11 |
|  | | | Cholesterol esters in medium HDL (mmol/l) | | | | 0.81 (0.63, 1.04) | 0.10 |
|  | | | Free cholesterol in medium HDL (mmol/l) | | | | 0.86 (0.70, 1.06) | 0.16 |
|  | | | Triglycerides in medium HDL (mmol/l) | | | | 0.84 (0.69, 1.02) | 0.08 |
| **Small HDL** | | | | | | |  |  |
|  | | | Concentration of small HDL particles (mol/l) | | | | 0.96 (0.85, 1.07) | 0.46 |
|  | | | Total lipids in small HDL (mmol/l) | | | | 0.87 (0.60, 1.25) | 0.45 |
|  | | | Phospholipids in small HDL (mmol/l) | | | | 0.85 (0.62, 1.16) | 0.31 |
|  | | | Total cholesterol in small HDL (mmol/l) | | | | 0.95 (0.71, 1.27) | 0.73 |
|  | | | Cholesterol esters in small HDL (mmol/l) | | | | 0.99 (0.77, 1.26) | 0.91 |
|  | | | Free cholesterol in small HDL (mmol/l) | | | | 0.88 (0.69, 1.13) | 0.33 |
|  | | | Triglycerides in small HDL (mmol/l) | | | | 1.01 (0.86, 1.19) | 0.86 |
| **Chylomicrons and extremely large VLDL ratios** | | | | | | |  |  |
|  | | Phospholipids to total lipids ratio in chylomicrons and extremely large VLDL (%) | | | | | 0.88 (0.75, 1.03) | 0.11 |
|  | | Total cholesterol to total lipids ratio in chylomicrons and extremely large VLDL (%) | | | | | 0.82 (0.72, 0.94) | **0.003** |
| **METABOLITE** | | | | | | | **HR (95% CI)** | **p-value** |
| **Chylomicrons and extremely large VLDL ratios** | | | | | | |  |  |
|  | | Cholesterol esters to total lipids ratio in chylomicrons and extremely large VLDL (%) | | | | | 0.89 (0.78, 1.02) | 0.09 |
|  | | Free cholesterol to total lipids ratio in chylomicrons and extremely large VLDL (%) | | | | | 0.83 (0.72, 0.95) | **0.01** |
|  | | Triglycerides to total lipids ratio in chylomicrons and extremely large VLDL (%) | | | | | 1.18 (1.05, 1.34) | **0.01** |
| **Very large VLDL ratios** | | | | | | |  |  |
|  | | | Phospholipids to total lipids ratio in very large VLDL (%) | | | | 0.94 (0.85, 1.04) | 0.26 |
|  | | | Total cholesterol to total lipids ratio in very large VLDL (%) | | | | 0.98 (0.87, 1.10) | 0.73 |
|  | | | Cholesterol esters to total lipids ratio in very large VLDL (%) | | | | 0.97 (0.87, 1.07) | 0.53 |
|  | | | Free cholesterol to total lipids ratio in very large VLDL (%) | | | | 1.00 (0.89, 1.12) | 0.99 |
|  | | | Triglycerides to total lipids ratio in very large VLDL (%) | | | | 0.95 (0.86, 1.05) | 0.28 |
| **Large VLDL ratios** | | | | | | |  |  |
|  | | | | Phospholipids to total lipids ratio in large VLDL (%) | | | 1.04 (0.91, 1.18) | 0.60 |
|  | | | | Total cholesterol to total lipids ratio in large VLDL (%) | | | 0.91 (0.79, 1.04) | 0.16 |
|  | | | | Cholesterol esters to total lipids ratio in large VLDL (% | | | 0.97 (0.86, 1.10) | 0.61 |
|  | | | | Free cholesterol to total lipids ratio in large VLDL (%) | | | 0.89 (0.79, 1.01) | 0.06 |
|  | | | | Triglycerides to total lipids ratio in large VLDL (%) | | | 1.07 (0.92, 1.26) | 0.39 |
| **Medium VLDL ratios** | | | | | | |  |  |
|  | | | | | Phospholipids to total lipids ratio in medium VLDL (%) | | 1.05 (0.93, 1.19) | 0.41 |
|  | | | | | Total cholesterol to total lipids ratio in medium VLDL (%) | | 0.95 (0.84, 1.08) | 0.44 |
|  | | | | | Cholesterol esters to total lipids ratio in medium VLDL (%) | | 0.97 (0.85, 1.09) | 0.57 |
|  | | | | | Free cholesterol to total lipids ratio in medium VLDL (%) | | 0.92 (0.80, 1.05) | 0.23 |
|  | | | | | Triglycerides to total lipids ratio in medium VLDL (%) | | 1.04 (0.89, 1.22) | 0.61 |
| **Small VLDL ratios** | | | | | | |  |  |
|  | | | Phospholipids to total lipids ratio in small VLDL (%) | | | | 0.99 (0.88, 1.12) | 0.90 |
|  | | | Total cholesterol to total lipids ratio in small VLDL (%) | | | | 1.00 (0.89, 1.12) | 0.99 |
|  | | | Cholesterol esters to total lipids ratio in small VLDL (%) | | | | 1.00 (0.89, 1.13) | 0.97 |
|  | | | | | Free cholesterol to total lipids ratio in small VLDL (%) | | 0.95 (0.84, 1.08) | 0.44 |
|  | | | | | Triglycerides to total lipids ratio in small VLDL (%) | | 0.99 (0.88, 1.11) | 0.81 |
| **Very small VLDL ratios** | | | | | | |  |  |
|  | | | Phospholipids to total lipids ratio in very small VLDL (%) | | | | 0.98 (0.87, 1.10) | 0.69 |
| **METABOLITE** | | | | | | | **HR (95% CI)** | **p-value** |
| **Very small VLDL ratios** | | | | | | |  |  |
|  | | | Total cholesterol to total lipids ratio in very small VLDL (%) | | | | 1.01 (0.89, 1.14) | 0.91 |
|  | | | Cholesterol esters to total lipids ratio in very small VLDL (%) | | | | 1.03 (0.91, 1.17) | 0.62 |
|  | | | Free cholesterol to total lipids ratio in very small VLDL (%) | | | | 0.93 (0.82, 1.04) | 0.19 |
|  | | | Triglycerides to total lipids ratio in very small VLDL (%) | | | | 0.98 (0.87, 1.10) | 0.71 |
| **IDL ratios** | | | | | | |  |  |
|  | | | | | Phospholipids to total lipids ratio in IDL (%) | | 0.93 (0.83, 1.06) | 0.28 |
|  | | | | | Total cholesterol to total lipids ratio in IDL (%) | | 0.96 (0.85, 1.08) | 0.50 |
|  | | | | | Cholesterol esters to total lipids ratio in IDL (%) | | 0.98 (0.87, 1.10) | 0.70 |
|  | | | | | Free cholesterol to total lipids ratio in IDL (%) | | 0.94 (0.84, 1.05) | 0.30 |
|  | | | | | Triglycerides to total lipids ratio in IDL (%) | | 1.03 (0.92, 1.16) | 0.57 |
| **Large LDL ratios** | | | | | | |  |  |
|  | | | | Phospholipids to total lipids ratio in large LDL (%) | | | 0.99 (0.87, 1.12) | 0.86 |
|  | | | | Total cholesterol to total lipids ratio in large LDL (%) | | | 0.95 (0.84, 1.08) | 0.46 |
|  | | | | Cholesterol esters to total lipids ratio in large LDL (%) | | | 0.95 (0.81, 1.10) | 0.46 |
|  | | | | Free cholesterol to total lipids ratio in large LDL (%) | | | 0.99 (0.88, 1.12) | 0.86 |
|  | | | | Triglycerides to total lipids ratio in large LDL (%) | | | 1.05 (0.93, 1.17) | 0.44 |
| **Medium LDL ratios** | | | | | | |  |  |
|  | | | | | | Phospholipids to total lipids ratio in medium LDL (%) | 1.03 (0.92, 1.15) | 0.59 |
|  | | | | | | Total cholesterol to total lipids ratio in medium LDL (%) | 0.96 (0.86, 1.08) | 0.53 |
|  | | | | | | Cholesterol esters to total lipids ratio in medium LDL (%) | 0.95 (0.84, 1.09) | 0.48 |
|  | | | | | | Free cholesterol to total lipids ratio in medium LDL (%) | 1.06 (0.93, 1.19) | 0.38 |
|  | | | | | | Triglycerides to total lipids ratio in medium LDL (%) | 1.04 (0.92, 1.16) | 0.56 |
| **Small LDL ratios** | | | | | | |  |  |
|  | | | | | Phospholipids to total lipids ratio in small LDL (%) | | 1.02 (0.91, 1.14) | 0.75 |
|  | | | | | Total cholesterol to total lipids ratio in small LDL (%) | | 0.98 (0.87, 1.10) | 0.72 |
|  | | | | | Cholesterol esters to total lipids ratio in small LDL (%) | | 0.97 (0.86, 1.11) | 0.70 |
|  | | | | | Free cholesterol to total lipids ratio in small LDL (%) | | 1.05 (0.93, 1.19) | 0.40 |
|  | | | | | Triglycerides to total lipids ratio in small LDL (%) | | 1.00 (0.89, 1.12) | 0.95 |
| **METABOLITE** | | | | | | | **HR (95% CI)** | **p-value** |
| **Very large HDL ratios** | | | | | | |  |  |
|  | | | | | Phospholipids to total lipids ratio in very large HDL (%) | | 1.00 (0.88, 1.13) | 0.97 |
|  | | | | | Total cholesterol to total lipids ratio in very large HDL (%) | | 1.02 (0.90, 1.16) | 0.72 |
|  | | | | | Cholesterol esters to total lipids ratio in very large HDL (%) | | 1.03 (0.90, 1.18) | 0.67 |
|  | | | | | Free cholesterol to total lipids ratio in very large HDL (%) | | 0.99 (0.88, 1.11) | 0.84 |
|  | | | | | Triglycerides to total lipids ratio in very large HDL (%) | | 0.93 (0.82, 1.04) | 0.21 |
| **Large HDL ratios** | | | | | | |  |  |
|  | | | | | Phospholipids to total lipids ratio in large HDL (%) | | 0.96 (0.86, 1.08) | 0.54 |
|  | | | | | Total cholesterol to total lipids ratio in large HDL (%) | | 1.05 (0.93, 1.18) | 0.45 |
|  | | | | | Cholesterol esters to total lipids ratio in large HDL (%) | | 1.06 (0.94, 1.19) | 0.34 |
|  | | | | | Free cholesterol to total lipids ratio in large HDL (%) | | 1.01 (0.90, 1.14) | 0.82 |
|  | | | | | Triglycerides to total lipids ratio in large HDL (%) | | 0.96 (0.86, 1.08) | 0.54 |
| **Medium HDL ratios** | | | | | | |  |  |
|  | | | | | | Phospholipids to total lipids ratio in medium HDL (%) | 1.17 (1.05, 1.31) | **0.01** |
|  | | | | | | Total cholesterol to total lipids ratio in medium HDL (%) | 0.93 (0.83, 1.04) | 0.18 |
|  | | | | | | Cholesterol esters to total lipids ratio in medium HDL (%) | 0.93 (0.83, 1.04) | 0.21 |
|  | | | | | | Free cholesterol to total lipids ratio in medium HDL (%) | 0.94 (0.84, 1.06) | 0.31 |
|  | | | | | | Triglycerides to total lipids ratio in medium HDL (%) | 0.97 (0.86, 1.09) | 0.57 |
| **Small HDL ratios** | | | | | | |  |  |
|  | | | | | Phospholipids to total lipids ratio in small HDL (%) | | 0.95 (0.85, 1.06) | 0.39 |
|  | | | | | Total cholesterol to total lipids ratio in small HDL (%) | | 1.02 (0.92, 1.14) | 0.67 |
|  | | | | | Cholesterol esters to total lipids ratio in small HDL (%) | | 1.03 (0.92, 1.15) | 0.62 |
|  | | | | | Free cholesterol to total lipids ratio in small HDL (%) | | 0.94 (0.84, 1.04) | 0.24 |
|  | | | | | Triglycerides to total lipids ratio in small HDL (%) | | 1.03 (0.92, 1.16) | 0.61 |
| **Lipoprotein particle sizes** | | | | | | |  |  |
|  | | | | Mean diameter for VLDL particles (nm) | | | 0.97 (0.86, 1.09) | 0.63 |
|  | | | | Mean diameter for LDL particles (nm) | | | 1.00 (0.89, 1.13) | 0.99 |
|  | | | | Mean diameter for HDL particles (nm) | | | 1.00 (0.89, 1.13) | 0.99 |

| **METABOLITE** | | | | | | | | **HR (95% CI)** | **p-value** |
| --- | --- | --- | --- | --- | --- | --- | --- | --- | --- |
| **Cholesterol related metabolites** | | | | | | | |  |  |
|  | | Serum total cholesterol (mmol/l) | | | | | | 0.96 (0.86, 1.08) | 0.51 |
|  | | Total cholesterol in VLDL (mmol/l) | | | | | | 0.98 (0.86, 1.10) | 0.68 |
|  | | Remnant cholesterol (non-HDL, non-LDL -cholesterol) (mmol/l) | | | | | | 0.98 (0.87, 1.10) | 0.73 |
|  | | Total cholesterol in LDL (mmol/l) | | | | | | 0.98 (0.87, 1.11) | 0.76 |
|  | | Total cholesterol in HDL (mmol/l) | | | | | | 0.96 (0.86, 1.08) | 0.54 |
|  | | Total cholesterol in HDL2 (mmol/l) | | | | | | 0.96 (0.85, 1.07) | 0.45 |
|  | | Total cholesterol in HDL3 (mmol/l) | | | | | | 1.04 (0.93, 1.17) | 0.45 |
|  | | Esterified cholesterol (mmol/l) | | | | | | 0.97 (0.86, 1.09) | 0.58 |
|  | | Free cholesterol (mmol/l) | | | | | | 0.95 (0.84, 1.06) | 0.36 |
| **Lipids related metabolites** | | | | | | | |  |  |
|  | Serum total triglycerides (mmol/l) | | | | | | | 0.98 (0.87, 1.10) | 0.70 |
|  | | | Triglycerides in VLDL (mmol/l) | | | | | 0.98 (0.87, 1.10) | 0.70 |
|  | | | Triglycerides in LDL (mmol/l) | | | | | 1.02 (0.91, 1.15) | 0.75 |
|  | | | Triglycerides in HDL (mmol/l) | | | | | 0.93 (0.83, 1.05) | 0.25 |
|  | | | Diacylglycerol (mmol/l) | | | | | 0.97 (0.87, 1.10) | 0.67 |
|  | | | Ratio of diacylglycerol to triglycerides | | | | | 0.98 (0.87, 1.10) | 0.69 |
|  | | | Total phosphoglycerides (mmol/l) | | | | | 0.94 (0.83, 1.06) | 0.29 |
|  | | | Ratio of triglycerides to phosphoglycerides | | | | | 0.98 (0.88, 1.11) | 0.79 |
|  | | | Phosphatidylcholine and other cholines (mmol/l) | | | | | 0.94 (0.83, 1.06) | 0.28 |
|  | | | Sphingomyelins (mmol/l) | | | | | 0.91 (0.81, 1.01) | 0.09 |
|  | | | Total cholines (mmol/l) | | | | | 0.93 (0.82, 1.04) | 0.21 |
| **Fatty acids related metabolites** | | | | | | | |  |  |
|  | | Total fatty acids (mmol/l) | | | | | | 0.96 (0.86, 1.08) | 0.54 |
|  | | Estimated description of fatty acid chain length, not actual carbon number | | | | | | 0.98 (0.88, 1.10) | 0.76 |
|  | | Unsaturation degree | | | | | | 0.93 (0.83, 1.05) | 0.24 |
|  | | 22:6, docosahexaenoic acid (mmol/l) | | | | | | 0.95 (0.85, 1.07) | 0.41 |
|  | | 18:2, linoleic acid (mmol/l) | | | | | | 0.92 (0.82, 1.03) | 0.16 |
|  | | Conjugated linoleic acid (mmol/l) | | | | | | 0.98 (0.88, 1.10) | 0.76 |
|  | | Omega-3 fatty acids (mmol/l) | | | | | | 0.97 (0.86, 1.08) | 0.58 |
| **METABOLITE** | | | | | | | | **HR (95% CI)** | **p-value** |
| **Fatty acids related metabolites** | | | | | | | |  |  |
|  | | Omega-6 fatty acids (mmol/l) | | | | | | 0.91 (0.81, 1.02) | 0.11 |
|  | | Polyunsaturated fatty acids (mmol/l) | | | | | | 0.92 (0.82, 1.03) | 0.14 |
|  | | Monounsaturated fatty acids; 16:1, 18:1 (mmol/l) | | | | | | 0.99 (0.88, 1.11) | 0.84 |
|  | | Saturated fatty acids (mmol/l) | | | | | | 0.99 (0.88, 1.11) | 0.81 |
|  | | Ratio of 22:6 docosahexaenoic acid to total fatty acids (%) | | | | | | 0.97 (0.86, 1.09) | 0.58 |
|  | | Ratio of 18:2 linoleic acid to total fatty acids (%) | | | | | | 0.94 (0.84, 1.05) | 0.26 |
|  | | Ratio of conjugated linoleic acid to total fatty acids (%) | | | | | | 0.99 (0.88, 1.12) | 0.89 |
|  | | Ratio of omega-3 fatty acids to total fatty acids (%) | | | | | | 0.99 (0.88, 1.10) | 0.82 |
|  | | Ratio of omega-6 fatty acids to total fatty acids (%) | | | | | | 0.92 (0.82, 1.02) | 0.12 |
|  | | Ratio of polyunsaturated fatty acids to total fatty acids (%) | | | | | | 0.92 (0.82, 1.03) | 0.14 |
|  | | Ratio of monounsaturated fatty acids to total fatty acids (%) | | | | | | 1.03 (0.91, 1.15) | 0.66 |
|  | | Ratio of saturated fatty acids to total fatty acids (%) | | | | | | 1.07 (0.96, 1.20) | 0.20 |
| **Apoliporproteins related metabolites** | | | | | | | |  |  |
|  | | Apolipoprotein a-i (g/l) | | | | | | 0.94 (0.84, 1.06) | 0.30 |
|  | | Apolipoprotein b (g/l) | | | | | | 0.99 (0.88, 1.11) | 0.82 |
|  | | Ratio of apolipoprotein b to apolipoprotein a-i | | | | | | 1.02 (0.91, 1.15) | 0.70 |
| **Glycolisis related metabolites** | | | | | | | |  |  |
|  | | Glucose (mmol/l) | | | | | | 1.23 (1.11, 1.35) | **0.00004*** |
|  | | | | Lactate (mmol/l) | | | | 0.96 (0.86, 1.07) | 0.51 |
|  | | | | Pyruvate (mmol/l) | | | | 1.02 (0.92, 1.14) | 0.66 |
|  | | | | Citrate (mmol/l) | | | | 1.05 (0.94, 1.18) | 0.37 |
|  | | | | Glycerol (mmol/l) | | | | 1.00 (0.90, 1.11) | 0.97 |
| **Amino acids** | | | | | | | |  |  |
|  | | | | | Alanine (mmol/l) | | | 0.92 (0.82, 1.02) | 0.12 |
|  | | | | | Glutamine (mmol/l) | | | 0.98 (0.87, 1.11) | 0.74 |
|  | | | | | Glycine (mmol/l) | | | 0.95 (0.85, 1.07) | 0.41 |
|  | | | | | Histidine (mmol/l) | | | 0.99 (0.88, 1.10) | 0.81 |
|  | | | | | Isoleucine (mmol/l) | | | 1.01 (0.90, 1.14) | 0.88 |
|  | | | | | Leucine (mmol/l) | | | 0.97 (0.86, 1.09) | 0.61 |
| **METABOLITE** | | | | | | | | **HR (95% CI)** | **p-value** |
| **Amino acids** | | | | | | | |  |  |
|  | | | | | Valine (mmol/l) | | | 1.01 (0.89, 1.14) | 0.88 |
|  | | | | | Phenylalanine (mmol/l) | | | 1.00 (0.90, 1.13) | 0.93 |
|  | | | | | Tyrosine (mmol/l) | | | 0.98 (0.87, 1.09) | 0.67 |
| **Ketone bodies** | | | | | | | |  |  |
|  | | | | | Acetate (mmol/l) | | | 0.95 (0.85, 1.07) | 0.43 |
|  | | | | | Acetoacetate (mmol/l) | | | 1.10 (0.99, 1.22) | 0.09 |
|  | | | | | β-hydroxybutyrate (mmol/l) | | | 1.11 (0.99, 1.23) | 0.06 |
| **Fluid balance** | | | | | | | |  |  |
|  | | | | | | | Creatinine (mmol/l) | 0.92 (0.80, 1.05) | 0.22 |
|  | | | | | | | Albumin (signal area) | 0.91 (0.82, 1.01) | 0.09 |
| **Inflammation** | | | | | | | |  |  |
|  | | | | | | Glycoprotein acetyls, mainly a1-acid glycoprotein (mmol/l) | | 0.94 (0.84, 1.06) | 0.32 |

VLDL: very low density lipoproteins, IDL: intermediate-density lipoproteins, LDL: low density lipoproteins, HDL: high density lipoprotein.

Data are M (SD).

Cox proportional hazard regression adjusted for age, sex, education and ethnicity.

* Metabolites significantly associated at p<0.0002 (Bonferroni correction for multiple testing).

**Table S5. Elastic net penalized Cox regression with repeated nested cross-validation for incident dementia: results of 100 repetitions.**

| **Repetition** | **α*** | **λ*** | **c-statistic best model^†^** | **c-statistic age model^‡^** | **p-value^§^** |
| --- | --- | --- | --- | --- | --- |
| 1 | 1 | 0.00617437 | 0.707 | 0.710 | 0.71 |
| 2 | 0.9 | 0.00617437 | 0.760 | 0.749 | **0.01** |
| 3 | 1 | 0.00617437 | 0.748 | 0.750 | 0.67 |
| 4 | 1 | 0.00617437 | 0.724 | 0.715 | **0.007** |
| 5 | 1 | 0.00467068 | 0.746 | 0.755 | 0.87 |
| 6 | 1 | 0.00816215 | 0.713 | 0.716 | 0.67 |
| 7 | 1 | 0.00167856 | 0.767 | 0.779 | 0.86 |
| 8 | 1 | 0.00562585 | 0.763 | 0.767 | 0.78 |
| 9 | 1 | 0.00743705 | 0.718 | 0.715 | 0.19 |
| 10 | 1 | 0.00677636 | 0.747 | 0.741 | **0.04** |
| 11 | 0.7 | 0.00562585 | 0.742 | 0.754 | 0.95 |
| 12 | 1 | 0.00425575 | 0.736 | 0.741 | 0.79 |
| 13 | 1 | 0.00512607 | 0.703 | 0.707 | 0.67 |
| 14 | 1 | 0.00512607 | 0.770 | 0.775 | 0.78 |
| 15 | 1 | 0.00467068 | 0.775 | 0.779 | 0.80 |
| 16 | 1 | 0.00512607 | 0.775 | 0.765 | **0.02** |
| 17 | 0.4 | 0.01184188 | 0.752 | 0.752 | 0.45 |
| 18 | 0.7 | 0.01299645 | 0.742 | 0.738 | **0.007** |
| 19 | 1 | 0.0035332 | 0.754 | 0.759 | 0.79 |
| 20 | 1 | 0.00562585 | 0.749 | 0.760 | 0.96 |
| 21 | 1 | 0.00562585 | 0.713 | 0.705 | 0.13 |
| 22 | 1 | 0.00816215 | 0.703 | 0.696 | **0.02** |
| 23 | 1 | 0.00425575 | 0.779 | 0.763 | **0.001** |
| 24 | 1 | 0.00562585 | 0.696 | 0.688 | 0.08 |
| 25 | 1 | 0.00677636 | 0.734 | 0.734 | 0.51 |
| 26 | 1 | 0.00617437 | 0.750 | 0.756 | 0.82 |
| 27 | 0.2 | 0.01078988 | 0.730 | 0.738 | 0.83 |
| 28 | 0.8 | 0.01078988 | 0.728 | 0.727 | 0.28 |
| 29 | 1 | 0.00895795 | 0.775 | 0.775 | 0.53 |
| 30 | 1 | 0.00617437 | 0.746 | 0.736 | **0.009** |
| 31 | 0.7 | 0.00743705 | 0.744 | 0.742 | 0.34 |
| 32 | 1 | 0.00743705 | 0.718 | 0.718 | 0.59 |
| 33 | 0.6 | 0.00895795 | 0.779 | 0.777 | 0.34 |
| 34 | 0.5 | 0.00895795 | 0.728 | 0.723 | 0.25 |
| 35 | 1 | 0.00743705 | 0.713 | 0.707 | 0.08 |
| 36 | 1 | 0.00512607 | 0.735 | 0.729 | 0.17 |
| 37 | 1 | 0.00743705 | 0.750 | 0.752 | 0.64 |
| 38 | 1 | 0.00617437 | 0.718 | 0.711 | **0.04** |
| 39 | 1 | 0.00512607 | 0.749 | 0.759 | 0.92 |
| 40 | 1 | 0.00617437 | 0.778 | 0.779 | 0.55 |
| 41 | 0.2 | 0.01184188 | 0.754 | 0.757 | 0.65 |
| 42 | 0 | 0.01299645 | 0.757 | 0.763 | 0.67 |
| 43 | 1 | 0.00743705 | 0.742 | 0.742 | 0.54 |
| **Repetition** | **α*** | **λ*** | **C-statistic best model^†^** | **C-statistic age model^‡^** | **p-value^§^** |
| 44 | 1 | 0.00512607 | 0.679 | 0.671 | 0.06 |
| 45 | 1 | 0.00562585 | 0.724 | 0.725 | 0.60 |
| 46 | 0.4 | 0.01078988 | 0.705 | 0.695 | 0.06 |
| 47 | 1 | 0.00617437 | 0.711 | 0.713 | 0.60 |
| 48 | 0.7 | 0.00512607 | 0.751 | 0.754 | 0.66 |
| 49 | 1 | 0.0035332 | 0.749 | 0.759 | 0.86 |
| 50 | 1 | 0.00562585 | 0.745 | 0.734 | **0.01** |
| 51 | 0.2 | 0.01299645 | 0.788 | 0.792 | 0.71 |
| 52 | 0.5 | 0.00816215 | 0.723 | 0.723 | 0.49 |
| 53 | 1 | 0.00467068 | 0.697 | 0.697 | 0.53 |
| 54 | 1 | 0.00562585 | 0.733 | 0.729 | 0.26 |
| 55 | 0.4 | 0.01299645 | 0.726 | 0.717 | 0.10 |
| 56 | 1 | 0.00617437 | 0.777 | 0.775 | 0.31 |
| 57 | 1 | 0.00467068 | 0.747 | 0.731 | **0.0006** |
| 58 | 1 | 0.00425575 | 0.785 | 0.787 | 0.63 |
| 59 | 1 | 0.00293332 | 0.737 | 0.724 | 0.13 |
| 60 | 0.5 | 0.00983134 | 0.753 | 0.753 | 0.50 |
| 61 | 0.3 | 0.01299645 | 0.742 | 0.732 | 0.06 |
| 62 | 1 | 0.00512607 | 0.766 | 0.770 | 0.71 |
| 63 | 0.3 | 0.01299645 | 0.737 | 0.729 | 0.08 |
| 64 | 1 | 0.00512607 | 0.793 | 0.789 | 0.23 |
| 65 | 1 | 0.00425575 | 0.734 | 0.733 | 0.44 |
| 66 | 1 | 0.00425575 | 0.732 | 0.745 | 0.95 |
| 67 | 0.5 | 0.00983134 | 0.755 | 0.743 | **0.004** |
| 68 | 0.9 | 0.00677636 | 0.701 | 0.705 | 0.70 |
| 69 | 0.8 | 0.00816215 | 0.729 | 0.725 | 0.23 |
| 70 | 0.4 | 0.01184188 | 0.759 | 0.763 | 0.79 |
| 71 | 0.3 | 0.01299645 | 0.734 | 0.726 | 0.05 |
| 72 | 0.7 | 0.00467068 | 0.758 | 0.749 | 0.08 |
| 73 | 0 | 0.01299645 | 0.741 | 0.754 | 0.84 |
| 74 | 0.8 | 0.00816215 | 0.735 | 0.726 | **0.02** |
| 75 | 0.3 | 0.01184188 | 0.713 | 0.716 | 0.70 |
| 76 | 1 | 0.00512607 | 0.722 | 0.719 | 0.32 |
| 77 | 1 | 0.00467068 | 0.767 | 0.778 | 0.94 |
| 78 | 0.8 | 0.00562585 | 0.708 | 0.718 | 0.91 |
| 79 | 1 | 0.00425575 | 0.742 | 0.742 | 0.52 |
| 80 | 1 | 0.00512607 | 0.724 | 0.726 | 0.64 |
| 81 | 1 | 0.00743705 | 0.757 | 0.755 | 0.36 |
| 82 | 1 | 0.00387768 | 0.782 | 0.779 | 0.31 |
| 83 | 0.8 | 0.00562585 | 0.721 | 0.724 | 0.66 |
| 84 | 1 | 0.00467068 | 0.764 | 0.764 | 0.46 |
| 85 | 1 | 0.00467068 | 0.768 | 0.783 | 0.98 |
| 86 | 1 | 0.00512607 | 0.696 | 0.701 | 0.72 |
| 87 | 1 | 0.00816215 | 0.778 | 0.779 | 0.57 |
| 88 | 1 | 0.00743705 | 0.758 | 0.761 | 0.76 |
| **Repetition** | **α*** | **λ*** | **C-statistic best model^†^** | **C-statistic age model^‡^** | **p-value^§^** |
| 89 | 1 | 0.00512607 | 0.736 | 0.733 | 0.32 |
| 90 | 0.6 | 0.01299645 | 0.696 | 0.694 | 0.30 |
| 91 | 1 | 0.00677636 | 0.724 | 0.714 | **0.008** |
| 92 | 0.4 | 0.01078988 | 0.741 | 0.735 | 0.17 |
| 93 | 1 | 0.00983134 | 0.730 | 0.728 | 0.31 |
| 94 | 0.7 | 0.00677636 | 0.762 | 0.751 | **0.03** |
| 95 | 0.4 | 0.01299645 | 0.683 | 0.675 | 0.11 |
| 96 | 0.5 | 0.00743705 | 0.735 | 0.722 | **0.04** |
| 97 | 0.9 | 0.00387768 | 0.762 | 0.775 | 0.93 |
| 98 | 0.6 | 0.00816215 | 0.724 | 0.724 | 0.50 |
| 99 | 0.8 | 0.00677636 | 0.708 | 0.713 | 0.74 |
| 100 | 0 | 0.01299645 | 0.686 | 0.671 | 0.15 |

^*^ These are hyperparameters, allowing selection of the model with the lowest partial likelihood deviance in the inner loop; α ranges from 0 to 1 with a value of 0 indicating that all predictors are retained in the model, λ controls the shrinkage of the beta coefficient.

^†^ c-statistic, in the validation fold of the outer loop, of the best model (lowest partial likelihood deviance in the training folds of the outer loop).

^‡^ c-statistic of the age-only model in the validation fold of the best outer loop model.

^§^ *p*-value for difference in c-statistic between the best model and the age-only model.

**Table S6.** **The beta coefficients from Cox regression used in the calculation of risk scores^*^.**

| **Predictors** | **Age only** | **Risk score 1** | **Risk score 2** | **Risk score 3** | **Risk score 4** | **Risk score 5** |
| --- | --- | --- | --- | --- | --- | --- |
| Age (years) | 1.112 | 1.109 | 1.095 | 1.099 | 1.084 | 1.079 |
| Glucose (mmol/l) | - | 0.220 | 0.208 | 0.214 | 0.222 | 0.252 |
| Phospholipids to total lipids ratio in medium HDL (%) | - | - | 0.132 | 0.142 | 0.169 | 0.353 |
| Creatinine (mmol/l) | - | - | - | - 0.133 | - 0.134 | - 0.106 |
| Triglycerides to total lipids ratio in very large VLDL (%) | - | - | - | - | 0.179 | 0.022 |
| Phospholipids to total lipids ratio in medium VLDL (%) | - | - | - | - | - | - 0.038 |
| Alanine (mmol/l) | - | - | - | - | - | - 0.109 |
| β-hydroxybutyrate (mmol/l) | - | - | - | - | - | - 0.035 |
| Free cholesterol to total lipids ratio in small HDL (%) | - | - | - | - | - | - 0.192 |
| Citrate (mmol/l) | - | - | - | - | - | 0.107 |
| Free cholesterol to total lipids ratio in very large VLDL (%) | - | - | - | - | - | 0.017 |
| Free cholesterol to total lipids ratio in large HDL (%) | - | - | - | - | - | 0.079 |
| Triglycerides to total lipids ratio in medium HDL (%) | - | - | - | - | - | - 0.188 |
| Phospholipids to total lipids ratio in small HDL (%) | - | - | - | - | - | 0.013 |
| Sphingomyelins (mmol/l) | - | - | - | - | - | -0.078 |
| Albumin (signal area) | - | - | - | - | - | - 0.004 |

VLDL: very low density lipoproteins, HDL: high density lipoprotein.

^*^The risk scores 1 to 5 include predictors identified in 100%, ≥90%, ≥60%, ≥50%, or at least once in the selected models from 100 repetitions of the nested elastic net penalized Cox regression.

**Table S7.**  **The contribution of metabolites, considered separately, to the predictive accuracy of risk score 5 (N=5374).**

|  | | **HR (95% Confidence Interval)** | **R^2^ (95% Confidence Interval)** | **AIC** | **Δ AIC** | **Sensitivity**  **%** | **Specificity**  **%** | **c-statistic**  **(95% Confidence Interval)** | ***p*-value^*^** |
| --- | --- | --- | --- | --- | --- | --- | --- | --- | --- |
| **Risk score 5**^†^ | | 3.26 (2.87, 3.71) | 0.582 (0.511, 0.649) | 5147.7 | Ref. | 74.0 | 72.0 | 0.796 (0.774, 0.819) | Ref. |
| **Risk score 5 after excluding** | |  |  |  |  |  |  |  |  |
|  | Glucose | 3.15 (2.77, 3.58) | 0.558 (0.483, 0.623) | 5169.5 | 21.8 | 73.1 | 74.2 | 0.789 (0.767, 0.812) | **0.01** |
|  | Phospholipids to total lipids ratio in medium HDL | 3.19 (2.81, 3.63) | 0.567 (0.494, 0.637) | 5161.2 | 13.5 | 83.4 | 63.8 | 0.792 (0.770, 0.814) | 0.08 |
|  | Creatinine | 3.25 (2.86, 3.70) | 0.579 (0.504, 0.651) | 5150.6 | 2.9 | 79.0 | 67.4 | 0.796 (0.773, 0.818) | 0.66 |
|  | Triglycerides to total lipids ratio in very large VLDL | 3.26 (2.87, 3.71) | 0.581 (0.509, 0.653) | 5147.8 | 0.1 | 84.9 | 61.3 | 0.796 (0.774, 0.819) | 0.73 |
|  | Phospholipids to total lipids ratio in medium VLDL | 3.27 (2.87, 3.71) | 0.581 (0.511, 0.649) | 5147.9 | 0.2 | 72.6 | 73.4 | 0.796 (0.774, 0.818) | 0.56 |
|  | Alanine | 3.25 (2.86, 3.70) | 0.580 (0.500, 0.650) | 5149.9 | 2.2 | 83.9 | 62.7 | 0.794 (0.772, 0.817) | 0.07 |
|  | β-hydroxybutyrate | 3.26 (2.87, 3.71) | 0.582 (0.509, 0.657) | 5148.0 | 0.3 | 84.8 | 61.2 | 0.796 (0.773, 0.818) | 0.32 |
|  | Free cholesterol to total lipids ratio in small HDL | 3.24 (2.85, 3.68) | 0.579 (0.501, 0.653) | 5150.3 | 2.6 | 76.2 | 70.6 | 0.795 (0.773, 0.818) | 0.49 |
|  | Citrate | 3.25 (2.86, 3.70) | 0.579 (0.506, 0.649) | 5150.2 | 2.5 | 73.4 | 72.5 | 0.795 (0.773, 0.818) | 0.50 |
|  | Free cholesterol to total lipids ratio in very large VLDL | 3.26 (2.87, 3.71) | 0.582 (0.507, 0.652) | 5147.8 | 0.1 | 74.1 | 72.0 | 0.796 (0.774, 0.819) | 0.50 |
|  | Free cholesterol to total lipids ratio in large HDL | 3.26 (2.87, 3.71) | 0.581 (0.506, 0.653) | 5148.5 | 0.8 | 84.0 | 62.1 | 0.796 (0.774, 0.819) | 0.84 |
|  | Triglycerides to total lipids ratio in medium HDL | 3.24 (2.85, 3.68) | 0.580 (0.506, 0.652) | 5149.9 | 2.2 | 74.8 | 71.0 | 0.795 (0.773, 0.818) | 0.25 |
|  | Phospholipids to total lipids ratio in small HDL | 3.26 (2.87, 3.71) | 0.582 (0.501, 0.653) | 5147.8 | 0.1 | 74.1 | 71.9 | 0.796 (0.774, 0.819) | 0.74 |
|  | Sphingomyelins | 3.26 (2.87, 3.71) | 0.580 (0.505, 0.651) | 5149.1 | 1.4 | 73.6 | 72.5 | 0.796 (0.773, 0.818) | 0.71 |
|  | Albumin | 3.26 (2.87, 3.71) | 0.581 (0.504, 0.651) | 5147.7 | 0 | 74.0 | 72.0 | 0.796 (0.774, 0.819) | 0.92 |

R²=Royston’s R², AIC: Akaike information criterion, c-statistic=Harrell’s C-index, VLDL=very low density lipoproteins, HDL=high density lipoprotein

^*^*p*-value for difference in c-statistic using age-only model as reference.

^†^  Risk score 5 includes age, glucose, phospholipids to total lipids ratio in medium HDL (%), creatinine (mmol/l), triglycerides to total lipids ratio in very large VLDL (%), phospholipids to total lipids ratio in medium VLDL (%), alanine (mmol/l), β-hydroxybutyrate (mmol/l), free cholesterol to total lipids ratio in small HDL (%), citrate (mmol/l), free cholesterol to total lipids ratio in very large VLDL (%), free cholesterol to total lipids ratio in large HDL (%), triglycerides to total lipids ratio in medium HDL (%), phospholipids to total lipids ratio in small HDL (%), sphingomyelins (mmol/l) and albumin (signal area).

**Table S8. Predictive performance of risk scores for incident dementia; all models included ApoE (N=4494).**

| **Risk scores** | **HR (95% Confidence Interval)** | **R^2^ (95% Confidence Interval)** | **AIC** | **Δ AIC** | **Sensitivity**  **%** | **Specificity**  **%** | **C-Statistic**  **(95% Confidence Interval)** | ***p*-value*** |
| --- | --- | --- | --- | --- | --- | --- | --- | --- |
| Age & ApoE model | 3.08 (2.70, 3.51) | 0.581 (0.493, 0.665) | 4111.0 | Ref. | 81.7 | 64.9 | 0.791 (0.766, 0.817) | Ref. |
| Risk score 1^†^ | 3.16 (2.77, 3.60) | 0.597 (0.513, 0.681) | 4098.6 | - 12.4 | 88.5 | 57.1 | 0.797 (0.772, 0.822) | 0.05 |
| Risk score 2^‡^ | 3.17 (2.78, 3.61) | 0.600 (0.514, 0.679) | 4095.9 | - 15.1 | 86.4 | 59.2 | 0.797 (0.772, 0.822) | 0.08 |
| Risk score 3^§^ | 3.21 (2.82, 3.66) | 0.613 (0.532, 0.688) | 4086.0 | - 25.0 | 84.4 | 63.1 | 0.801 (0.776, 0.826) | **0.02** |
| Risk score 4^¶^ | 3.23 (2.84, 3.68) | 0.623 (0.539, 0.698) | 4077.6 | - 33.4 | 79.5 | 66.3 | 0.803 (0.778, 0.829) | **0.02** |
| Risk score 5^#^ | 3.31 (2.91, 3.76) | 0.636 (0.553, 0.713) | 4066.4 | - 44.6 | 81.6 | 64.1 | 0.808 (0.783, 0.833) | **0.001** |

ApoE=Apolipoprotein E, R²=Royston’s R², AIC= Akaike information criterion, C-Statistic=Harrell’s C-index

^*^*p* values for difference in C-statistic using age & ApoE model as reference.

^†^ Risk score 1 includes age, ApoE and glucose.

^‡^ Risk score 2 includes age, ApoE, glucose and phospholipids to total lipids ratio in medium HDL (%).

^§^ Risk score 3 includes age, ApoE, glucose, phospholipids to total lipids ratio in medium HDL (%) and creatinine (mmol/l).

^¶^ Risk score 4 includes age, ApoE, glucose, phospholipids to total lipids ratio in medium HDL (%), creatinine (mmol/l) and triglycerides to total lipids ratio in very large VLDL (%).

^#^ Risk score 5 includes age, ApoE, glucose, phospholipids to total lipids ratio in medium HDL (%), creatinine (mmol/l), triglycerides to total lipids ratio in very large VLDL (%), phospholipids to total lipids ratio in medium VLDL (%), alanine (mmol/l), β-hydroxybutyrate (mmol/l), free cholesterol to total lipids ratio in small HDL (%), citrate (mmol/l), free cholesterol to total lipids ratio in very large VLDL (%), free cholesterol to total lipids ratio in large HDL (%), triglycerides to total lipids ratio in medium HDL (%), phospholipids to total lipids ratio in small HDL (%), sphingomyelins (mmol/l) and albumin (signal area).

**Table S9. Comparison of the best risk score with metabolites previously identified using data from the Whitehall II cohort study (N=5374).**

| **Risk scores** | **HR (95% Confidence Interval)** | **R^2^ (95% Confidence Interval)** | **AIC** | **Δ AIC** | **Sensitivity**  **%** | **Specificity**  **%** | **C-Statistic**  **(95% Confidence Interval)** | ***p*-value*** | ***p*-value**^†^ |
| --- | --- | --- | --- | --- | --- | --- | --- | --- | --- |
| Age-only model | 3.04 (2.66, 3.47) | 0.525 (0.450, 0.593) | 5198.2 | Ref. | 75.5 | 70.3 | 0.780 (0.757, 0.802) | Ref. | <0.001 |
| **Risk scores without age** |  |  |  |  |  |  |  |  |  |
| Risk score 5 excluding age^‡^ | 1.54 (1.40, 1.70) | 0.130 (0.078, 0.185) | 5468.6 | 270.4 | 53.2 | 65.3 | 0.641 (0.612, 0.670) | <0.001 | <0.001 |
| Risk score Tynkkynen et al. ^§^ | 1.32 (1.19, 1.47) | 0.048 (0.016, 0.091) | 5513.4 | 315.2 | 51.3 | 63.6 | 0.573 (0.541, 0.606) | <0.001 | <0.001 |
| Risk score van der Lee et al. ^¶^ | 1.33 (1.20, 1.47) | 0.051 (0.021, 0.09) | 5511.1 | 312.9 | 55.8 | 57.0 | 0.587 (0.556, 0.617) | <0.001 | <0.001 |
| **Risk scores including age** |  |  |  |  |  |  |  |  |  |
| Risk score 5^#^ | 3.26 (2.87, 3.71) | 0.582 (0.511, 0.649) | 5147.7 | - 50.5 | 74.0 | 72.0 | 0.796 (0.774, 0.819) | <0.001 | Ref. |
| Risk score Tynkkynen + age | 3.08 (2.70, 3.52) | 0.537 (0.466, 0.610) | 5187.9 | - 10.3 | 78.9 | 68.0 | 0.784 (0.761, 0.806) | 0.14 | 0.004 |
| Risk score van der Lee + age | 3.05 (2.68, 3.48) | 0.531 (0.454, 0.601) | 5193.2 | - 5.0 | 77.4 | 67.8 | 0.782 (0.759, 0.804) | 0.26 | 0.001 |

R²=Royston’s R², AIC: Akaike information criterion, C-Statistic=Harrell’s C-index

^*^*p* values for difference in C-statistic using age-only model as reference.

^†^*p* values for difference in C-statistic using model 5 as reference.

^‡^ Risk score includes glucose, phospholipids to total lipids ratio in medium HDL (%), creatinine (mmol/l), triglycerides to total lipids ratio in very large VLDL (%), phospholipids to total lipids ratio in medium VLDL (%), alanine (mmol/l), β-hydroxybutyrate (mmol/l), free cholesterol to total lipids ratio in small HDL (%), citrate (mmol/l), free cholesterol to total lipids ratio in very large VLDL (%), free cholesterol to total lipids ratio in large HDL (%), triglycerides to total lipids ratio in medium HDL (%), phospholipids to total lipids ratio in small HDL (%), sphingomyelins (mmol/l) and albumin (signal area).

^§^ Risk score includes creatinine (mmol/l), ratio of saturated fatty acids to total fatty acids (%), isoleucine (mmol/l), leucine (mmol/l), valine (mmol/l), cholesterol esters to total lipids ratio in large HDL (%), total cholesterol in small VLDL (mmol/l), total cholesterol to total lipids ratio in very large VLDL (%), triglycerides to total lipids ratio in chylomicrons and extremely large VLDL (%), phospholipids to total lipids ratio in large HDL (%).

^¶^ Risk score includes total cholesterol in medium HDL (mmol/l), cholesterol esters in medium HDL (mmol/l), phospholipids in medium HDL (mmol/l), free cholesterol in small HDL (mmol/l), glutamine (mmol/l) and 22:6, docosahexaenoic acid (mmol/l).

^#^ Risk score includes age, glucose, phospholipids to total lipids ratio in medium HDL (%), creatinine (mmol/l), triglycerides to total lipids ratio in very large VLDL (%), phospholipids to total lipids ratio in medium VLDL (%), alanine (mmol/l), β-hydroxybutyrate (mmol/l), free cholesterol to total lipids ratio in small HDL (%), citrate (mmol/l), free cholesterol to total lipids ratio in very large VLDL (%), free cholesterol to total lipids ratio in large HDL (%), triglycerides to total lipids ratio in medium HDL (%), phospholipids to total lipids ratio in small HDL (%), sphingomyelins (mmol/l) and albumin (signal area).

**Figure S1. Flow chart of sample selection.**

**Whitehall II Study, 1985-1988**

N=10308

**Excluded, 1985 to 1997**

- Deaths, N=306

- Lost to follow-up, N= 2132

**Participants at baseline of this study, 1997-1999**

N=7870

**Missing data**

- No participation in the clinical examination, N=1333

- Metabolite values under the limit of detection, N=1093

- Outlier values in metabolite concentrations, N=70

- Missing ApoE genotype, N=880

**ApoE analyses**

Participants included, N=4494

End of follow-up 31^st^ March, 2019

**Main analyses**

Participants in the analyses, N=5374

End of follow-up 31^st^ March, 2019
